# Chromatin 3D interaction analysis of the *STARD10* locus unveils *FCHSD2* as a regulator of insulin secretion

**DOI:** 10.1016/j.celrep.2021.108703

**Published:** 2021-02-02

**Authors:** Ming Hu, Inês Cebola, Gaelle Carrat, Shuying Jiang, Sameena Nawaz, Amna Khamis, Mickaël Canouil, Philippe Froguel, Anke Schulte, Michele Solimena, Mark Ibberson, Piero Marchetti, Fabian L. Cardenas-Diaz, Paul J. Gadue, Benoit Hastoy, Leonardo Alemeida-Souza, Harvey McMahon, Guy A. Rutter

**Affiliations:** 1 Section of Cell Biology and Functional Genomics, Department of Medicine, Imperial College London, Hammersmith Hospital, Du Cane Road, London W12 0NN, UK; 2 Section of Genetics and Genomics, Department of Metabolism, Digestion, and Reproduction, Imperial College London, Hammersmith Hospital, Du Cane Road, London W12 0NN, UK; 3 Oxford Centre for Diabetes Endocrinology and Metabolism, University of Oxford, Churchill Hospital, Headington, Oxford OX3 7LE, UK; 4 Université de Lille, CNRS, CHU Lille, Institut Pasteur de Lille, UMR 8199 - EGID, 59000 Lille, France; 5 Sanofi-Aventis Deutschland GmbH, 65926 Frankfurt am Main, Germany; 6 Paul Langerhans Institute of the Helmholtz Center Munich at the University Hospital and Faculty of Medicine, TU Dresden, 01307 Dresden, Germany; 7 Vital-IT Group, SIB Swiss Institute of Bioinformatics, 1015 Lausanne, Switzerland; 8 Department of Endocrinology and Metabolism, University of Pisa, 56126 Pisa, Italy; 9 Department of Pathology and Laboratory Medicine, University of Pennsylvania, Philadelphia, PA, USA; 10 Centre for Cellular and Molecular Therapeutics, Children’s Hospital of Philadelphia, Philadelphia, PA, USA; 11 HiLIFE Institute of Biotechnology & Faculty of Biological and Environmental Sciences, University of Helsinki, Helsinki, Finland; 12 MRC MRC Laboratory of Molecular Biology, Francis Crick Avenue, Cambridge CB2 0QH, UK; 13 Lee Kong Chian School of Medicine, Nan Yang Technological University, Singapore, Singapore

**Keywords:** type 2 diabetes, T2D, GWAS, genetic variant, enhancer cluster, chromatin structure, gene regulation, *STARD10*, *FCHSD2*, insulin secretion

## Abstract

Using chromatin conformation capture, we show that an enhancer cluster in the *STARD10* type 2 diabetes (T2D) locus forms a defined 3-dimensional (3D) chromatin domain. A 4.1-kb region within this locus, carrying 5 T2D-associated variants, physically interacts with CTCF-binding regions and with an enhancer possessing strong transcriptional activity. Analysis of human islet 3D chromatin interaction maps identifies the *FCHSD2* gene as an additional target of the enhancer cluster. CRISPR-Cas9-mediated deletion of the variant region, or of the associated enhancer, from human pancreas-derived EndoC-βH1 cells impairs glucose-stimulated insulin secretion. Expression of both *STARD10* and *FCHSD2* is reduced in cells harboring CRISPR deletions, and lower expression of *STARD10* and *FCHSD2* is associated, the latter nominally, with the possession of risk variant alleles in human islets. Finally, CRISPR-Cas9-mediated loss of *STARD10* or *FCHSD2,* but not *ARAP1,* impairs regulated insulin secretion. Thus, multiple genes at the *STARD10* locus influence β cell function.

## Highlights

• Type 2 risk variants at *STARD10* reside in CTCF-flanked enhancer clusters
• Loss of the risk variant-bearing region inhibits regulated insulin secretion
• 3D chromatin interaction maps reveal *FCHSD2* as target of the enhancer cluster
• Deletion of *STARD10* or *FCHSD2* from EndoC-βH1 β cells inhibits insulin secretion

## Graphical Abstract

**Figure:**
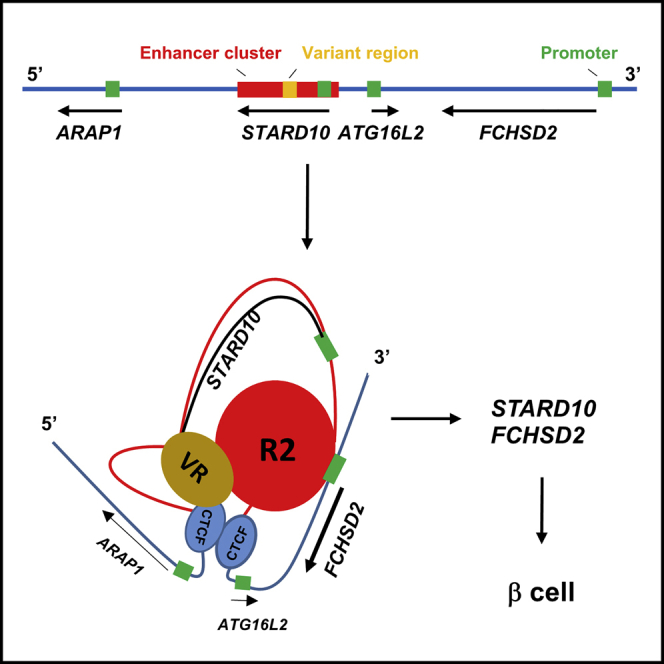

## Teaser

In this article, Hu et al. show the importance, for gene regulation, of a genomic region harboring GWAS variants that affect type 2 diabetes risk at the *STARD10* locus. They also identify FCHSD2 as a likely mediator of the effects of these variants on insulin secretion.

## Introduction

Genome-wide association studies (GWAS) have identified >400 genetic signals across >200 loci that associate with type 2 diabetes (T2D) risk (Mahajan et al., 2014, 2018; Morris et al., 2012; Scott et al., 2017; Spracklen et al., 2020; Voight et al., 2010). Data from these and other studies indicate that islet dysfunction plays a major role in T2D genetic risk. However, most associated genetic variants lie in intergenic or intronic regions of the genome, but only a minority affect protein sequences (Fuchsberger et al., 2016).

One plausible mechanism by which genetic variants contribute to T2D risk is by affecting functional noncoding sequences. Consisting of short DNA regions and located at varying distances from promoter sequences, enhancers are *cis*-regulatory elements that promote the expression of target genes due to their co-occupancy by tissue-enriched transcription factors and coactivators. T2D GWAS variants are enriched within pancreatic islet enhancer clusters, also called clusters of open regulatory elements (COREs), stretch enhancers, super-enhancers and, more recently, enhancer hubs (Gaulton et al., 2010; Miguel-Escalada et al., 2019; Parker et al., 2013; Pasquali et al., 2014). Enhancer clusters often control temporal and cell-specific functions and define cell identity (Gosselin et al., 2014; Huang et al., 2016; Whyte et al., 2013). Thus, genetic variants in islet enhancer clusters may contribute to diabetes risk by perturbing islet transcriptional networks. Consequently, in addition to the identification of causal variants, functional characterization of enhancer-target gene(s) interactions, and of their effect(s) on β cell function, are required to fully understand the genetic influence of T2D pathogenesis.

Enhancers interact with target gene(s) to regulate their expression, an effect achieved through chromatin looping, often mediated by the highly conserved architectural protein CTCF (CCCTC-binding factor) (Bonev and Cavalli, 2016; Williams and Flavell, 2008). CTCF contains a DNA-binding domain that recognizes a non-palindromic motif. Highlighting the relevance of CTCF sites in chromatin architecture and gene regulation, deletion, or inversion of CTCF-binding sites (CBSs) can affect chromatin looping and cause dysregulated gene expression (Guo et al., 2015; Mandegar et al., 2016; de Wit et al., 2015).

In our recent studies (Carrat et al., 2017), we used functional GWAS (fGWAS) (Pickrell, 2014) to fine map a diabetes-associated credible set in the *STARD10* (StAR-related lipid transfer protein 10) T2D GWAS locus, in which the risk haplotype has a global frequency of 86%. The identified credible set is composed of 8 variants, 5 of which displayed a posterior probability >0.05, in intron 2 of the *STARD10* gene. One of these (indel rs140130268), which possessed the highest probability, is located at the edge of a region of open chromatin (assay for transposase-accessible chromatin using sequencing [ATAC-seq]). Whether and how these variants affect the expression of local or remotely located genes in human β cells were not, however, examined in our earlier report.

In the present study, we have used human EndoC-βH1 cells, which recapitulate many of the functional properties of native human β cells (Ravassard et al., 2011), and deployed chromatin interaction analyses and β-cell tailored clustered regularly interspaced short palindromic repeats (CRISPR)-endonuclease from *Streptococcus pyogenes* (Cas9) genome editing to explore this question. We show that the variant region (VR) is required for normal glucose-stimulated insulin secretion and identify the enhancer regions with which it interacts physically. We also demonstrate direct roles for *STARD10* in human-derived β cell function. Finally, we provide genetic and functional evidence of a role for a previously unimplicated nearby gene, *FCHSD2* (FCH and double SH3 domains protein 2), encoding a regulator of membrane trafficking and endocytosis (Almeida-Souza et al., 2018), in variant action.

## Results

### Chromatin landscape at the *STARD10* locus

**Figure 1:**
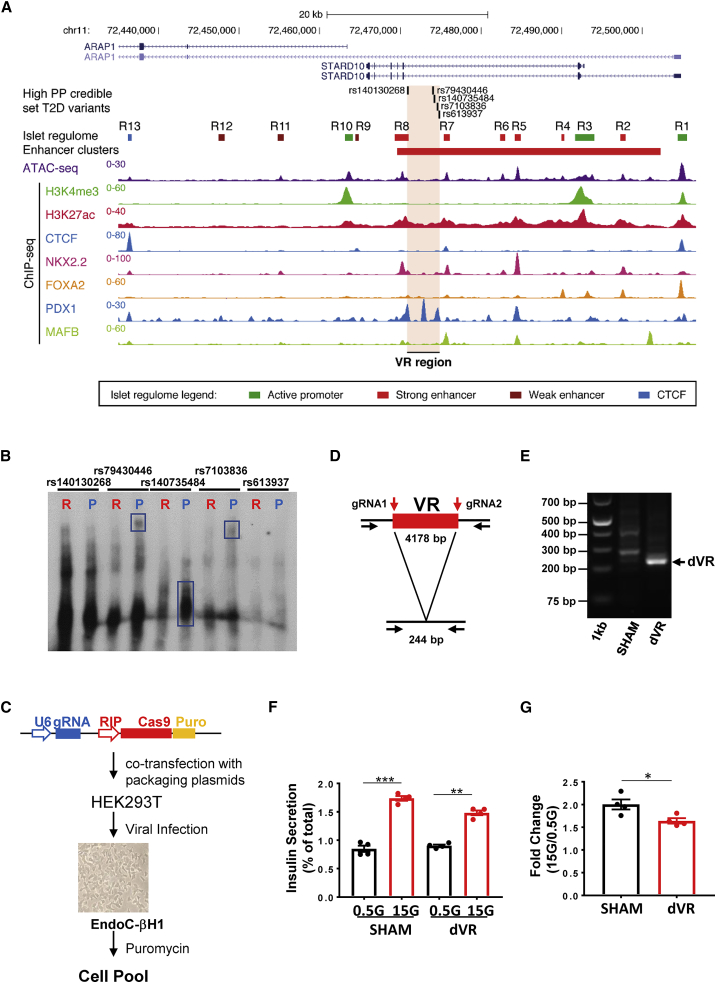
Variant region (VR) in local chromatin structure and β cell function (A) Epigenomic map of *STARD10* locus in human islets. The open chromatin regions identified by ATAC-seq were as R for regulatory region. Enhancer cluster: solid red bar. (B) Electrophoretic mobility shift assay (EMSA). R, risk allele; P, protective allele. n = 2. (C) Diagram of CRISPR-Cas9-mediated genome editing with a β cell-tailored vector via lentiviral approach. Lentiviruses were generated in HEK293T cells, titrated, and used to infect EndoC-βH1 cells. Puromycin was used to select viral resistant cells and generate a cell pool. (D) Strategy of VR deletion. Two gRNAs were designed to flank the VR region and generate a 4,178-bp deletion. (E) Electrophoresis of PCR products amplified from SHAM and VR-deleted (dVR) genomic DNA. Note that the bands in the SHAM lane (~300–400 bp) were non-specific products. (F) Representative data of glucose-stimulated insulin secretion (GSIS) assay. The experiment was performed in duplicate (n = 2) with insulin measurement in duplicate. (G) Fold change of secreted insulin. Data are normalized to insulin secretion at basal level (0.5 mM). The experiments were repeated 4 times (n = 4).

We investigated regulatory regions at the T2D GWAS locus close to *STARD10* by overlaying multiple human islet epigenomic datasets: ATAC-seq, histone marks associated with active chromatin (i.e., H3K27ac), and chromatin immunoprecipitation sequencing (ChIP-seq) for key islet transcription factors (TFs) (Miguel-Escalada et al., 2019; Pasquali et al., 2014). This analysis revealed multiple regulatory elements (R1–R13) that are active in human islets, including a cluster of 6 enhancers (Figure 1A). Several of these were bound by islet-enriched TFs such as NKX2.2, FOXA2, and MAFB, and are thus likely to contribute to an islet-specific gene expression signature. We also detected two binding sites for the chromatin architectural factor CTCF flanking the enhancer cluster, which may be involved in the creation of a distinct chromatin domain and mediate long-range looping with distal target genes (Figure 1A).

### Credible set variants exhibit differential transcription factor binding and transcriptional activity

We next turned our attention to the five variants in the credible set with the greatest causal probability, as defined previously by fine mapping and fGWAS analysis (Carrat et al., 2017). These variants span a 4.1-kb interval and include 2 deletions (indels rs140130268 and rs140735484) and 3 single nucleotide polymorphisms (SNPs; rs79430446, rs7103836, and rs613937). Of these, indel rs140130268 displayed the highest posterior probability and showed allele-specific transcriptional activity in β cells (Carrat et al., 2017). Detailed epigenomic analysis (Figure 1A) mapped these variants to the center of an enhancer cluster defined by strong H3K27ac enrichment in islet chromatin (Pasquali et al., 2014), although none of them resided within a previously mapped open chromatin region. We note, however, that the risk haplotype in this particular locus, which associates with lowered regulatory activity (Carrat et al., 2017), is present in 86% of the human population. Thus, it is possible that the existing regulatory maps do not represent the epigenomic landscape of carriers of the non-risk haplotype.

A likely mechanism by which the risk haplotype could confer reduced local chromatin accessibility is via the alteration of TF recognition sequences. To test this hypothesis, we performed TF motif analysis (Khan et al., 2018) on these variants, which suggested that four of the five variants may affect TF binding to this enhancer cluster (Table S1). To further explore this possibility, we assayed allele-specific TF binding by electrophoretic mobility shift assays (EMSAs), with oligonucleotides carrying either risk or protective variants. While differences were modest between risk and protective alleles for rs140130268, we observed marked differences in DNA-protein complex mobility between risk and protective alleles for rs79430446, rs140735484, and rs7103836 (Figure 1B). These results therefore point to a potential regulatory function of the variants in this credible set.

### Deletion of the VR from the EndoC-βH1 genome reduces insulin secretion

These 5 risk-bearing variants are located in a region between enhancers R7 and R8 (Figure 1A). This variant-containing region represents an area of open chromatin, as characterized by H3K27ac status and the binding of PDX1 (Figure 1A). To assess its importance, we deleted the entire 4.1-kb VR in EndoC-βH1 cells, which are homozygous for the risk haplotype, using CRISPR-Cas9-mediated genome editing (Figure 1C). To this end, we designed 2 guide RNAs (gRNAs) flanking the 4.1-kb genomic region that contains the T2D variants (Figure 1D). CRISPR-Cas9 genome editing was then performed in the glucose-responsive human β cell line EndoC-βH1 (Ravassard et al., 2011), delivering the gRNAs and the Cas9 gene under the control of the rat insulin promoter (RIP), and a puromycin resistance cassette. Deletion of the VR was confirmed by PCR (Figure 1E) and Sanger sequencing (Figure S2A). To control for possible non-specific effects of genome deletion and off-target CRISPR-Cas9 editing, we set up 2 controls, deleting regions of similar length in (1) an intergenic region between the *RAB6A* and *MRPL48* genes (Figures S1A and S2B) (this region is ~100 kb 3′ end of the *FCHSD2* promoter region) and (2) the β-globin locus (*HBB* gene) (Figures S1B and S2C). In addition, we used two scrambled gRNAs (Sc-gRNAs) that do not bind to the human genome as an additional control. gRNAs were designed using the Massachusetts Institute of Technology Genetic Perturbation Platform (MIT GPP) sgRNA designer platform (https://portals.broadinstitute.org/gpp/public/analysis-tools/sgrna-design) to minimize possible off-target sites.

Quantification by qPCR revealed that the remaining wild-type allele in dVR cells represented ~48% of the total. In addition, we detected DNA inversion after editing, corresponding to ~4.7% of the remaining wild-type alleles. Hence, the overall deletion efficiency was ~43% (Figures S3A–S3C). The deletion efficiencies for the intergenic region and the *HBB* region were 57.3% and 73.1%, respectively (Figures S3D–S3I).

To determine whether loss of the T2D variants may affect β cell function, we assayed glucose-stimulated insulin secretion (GSIS) in the presence (SHAM) or absence (dVR) of the VR. dVR cells displayed a small but significant reduction in GSIS (fold change: SHAM: 2.00 ± 0.11 versus dVR: 1.64 ± 0.06; p = 0.0267) (Figures 1F and 1G). In contrast, stimulation with neither glucose nor glucose plus the phosphodiesterase inhibitor isobutylmethylxanthine (IBMX) were altered in cell lines generated using Sc-RNAs or deleted for the *RAB6A-MRPL48* or *HBB* regions (Figures S3J and S3K).

### The T2D credible set variants interact with active islet regulatory elements

**Figure 2:**
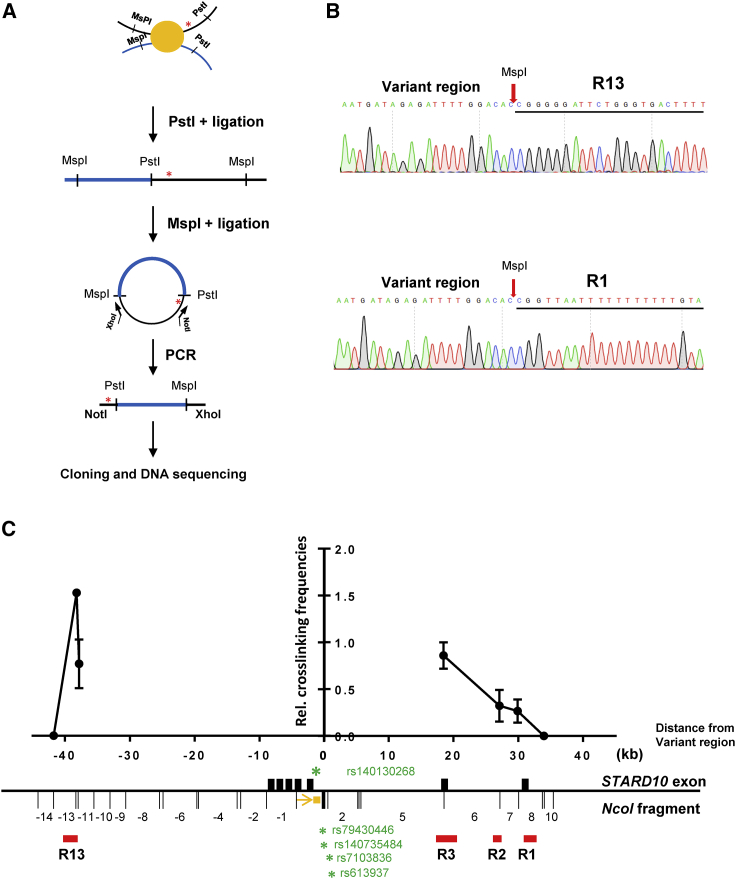
Identification and confirmation of genomic regions that physically interact with VR (A) Diagram of conventional 4C assay. (B) Representative DNA sequencing results showing ligation products between VR and interactive regions (R13 and R1 regions). (C) Representative 3C-qPCR data for chromatin interactions at *STARD10* locus. Viewpoint: VR region; black bar: *STARD10* exons; red bar: regulatory regions; orange box: qPCR probe; orange arrow: qPCR constant primer; green stars: credible set genetic variants. The numbering of *NcoI* fragments is given relative to the viewpoint. The leftmost dot corresponds to the 5′ end of the −13 DNA fragment. Note that the bait fragment contains rs140130268 only. rs79430446 and rs140735484 are in *Nco*I fragment 1; rs7103836 and rs613937 are in *NcoI* fragment 2; R13 is in *NcoI* fragment −13 and −12, while R1 is in *NcoI* fragment +8. Data were normalized to a *CXCL12* loading control; n = 3.

The 4.1-kb VR that encompasses the 5 T2D credible set variants lies between 2 active enhancers (Figure 1A), but it does not overlap with any annotated islet regulatory element. Since the above experiments demonstrated that this region is involved in the regulation of insulin secretion (Figures 1F and 1G), we hypothesized that it may contribute to β cell function by affecting chromatin topology and/or gene expression. To determine which genomic region(s) the VR may interact with, we performed circular chromosome conformation capture (4C) analysis (Göndör et al., 2008) (Figure 2A). Out of a total of 56 clones obtained after restriction enzyme digestion (PstI and *Msp*I) and DNA ligation, we detected 4 clones containing a DNA fragment within region R13, which corresponds to a strong CTCF site in human islets (Figure 2B). Moreover, 5 sequenced fragments mapped to a region 1.3 kb upstream of the R1 region, which contains 1 of the 2 promoters of *STARD10* that are active in human islets and is bound by CTCF (Figure 2B). The remaining fragments corresponded to DNA regions in the vicinity of the viewpoint, likely reflecting local chromatin collisions (Hagège et al., 2007).

To validate the 4C results above, we performed a 3C analysis (Figure 2C). Taking the T2D credible set variants region as a viewpoint, we detected higher interaction frequencies of the VR with both the CTCF site R13 and the 2 promoters of *STARD10* (R3 and R1). These results demonstrate that the T2D credible set variants region in *STARD10* undergo cis-interactions with human islet regulatory elements, including CTCF anchor points and the 2 promoters of *STARD10*.

### Identification of CBSs at the *STARD10* locus

**Figure 3:**
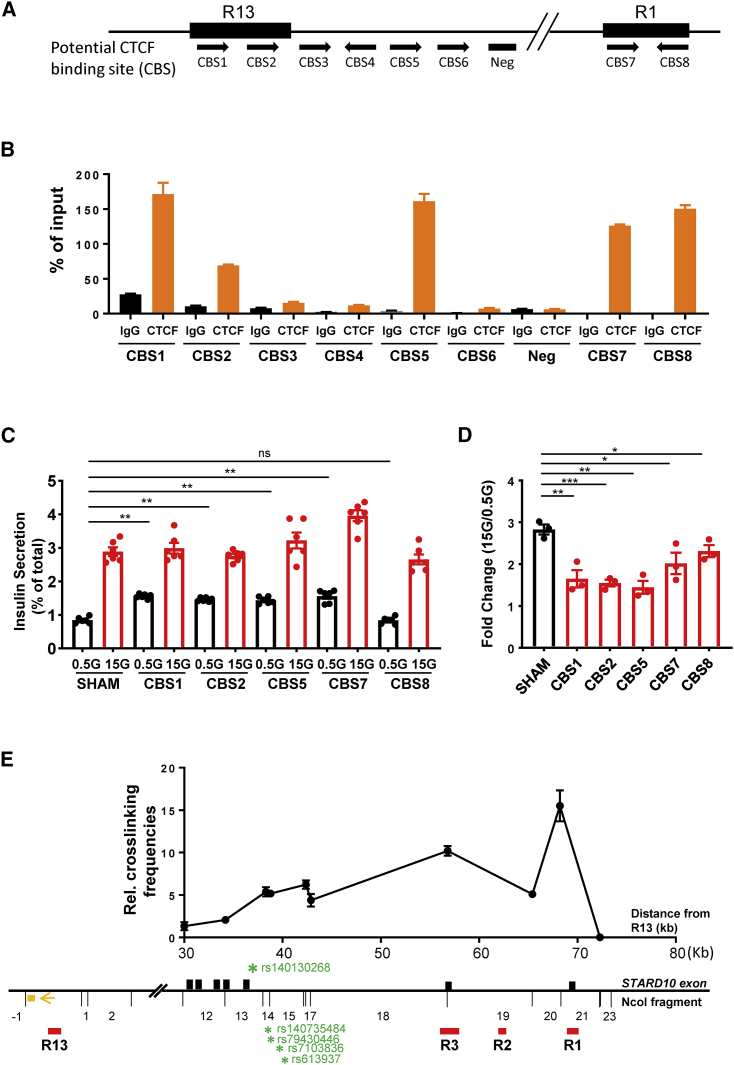
CTCF binding sites (CBSs) at R13 and R1 regions (A) Diagram of potential CBS within and surrounding R13 and R1 regions. Black arrows: binding orientation of CTCF. (B) Representative data of ChIP-qPCR analysis for CTCF binding at predicted binding sites; n = 3. (C) Representative data of GSIS assay; n = 3. (D) Fold change of secreted insulin. Data are normalized to insulin secretion at basal level (0.5 mM glucose); n = 3. (E) Representative 3C-qPCR data of chromatin interactions at *STARD10* locus. The numbering of *NcoI* DNA fragments is given relative to the viewpoint. Viewpoint: R13 region; black bars: *STARD10* exon; red bars: regulatory region; orange box: qPCR probe; orange arrow: qPCR constant primer; green stars: credible set genetic variants. Note that R1 is in the *NcoI* fragment +21. Data were normalized to a *CXCL12* loading control; n = 3.

Inspection of human islet ChIP-seq datasets, together with *in silico* TF binding motif analysis (Figures 1A and S4A), revealed that both R1 and R13 contain binding sites for critical islet TFs, including NKX2.2 and FOXA2, indicating their potential role in the regulation of islet gene expression. Of note, the two regions also showed enrichment for CTCF by ChIP-seq in human islets (Figure 1A). Using the ChIP-qPCR assay in EndoC-βH1 cells, we detected the binding of CTCF to five of the eight potential CTCF binding motifs (Figures 3A and 3B). More important, we detected CTCF enrichment at convergent CTCF-binding motif sequences, a configuration that has been previously shown to be involved in chromatin looping (de Wit et al., 2015).

### Mutation of CBSs leads to impaired insulin secretion

Higher-order chromatin structure is required for the regulation of cell-specific transcriptional activity (de Wit et al., 2015; Guo et al., 2015; Lupiáñez et al., 2015). Given the structural features of the *STARD10* locus described above, we investigated whether loss of key architectural elements in the *STARD10* locus could lead to β cell function impairment. Using CRISPR-Cas9, we mutated these identified CBSs individually in EndoC-βH1 cells. ChIP-qPCR for CTCF confirmed the significant loss of CTCF-binding ability at designated binding sites after CRISPR targeting (Figure S4B). In assays of GSIS, we observed that CRISPR-Cas9-mediated targeting of 4 of the 5 CBSs in the region (CBS1, CBS2, CBS5, and CBS7) led to increased basal insulin secretion (at 0.5 mM glucose) and lowered the fold change in secretion at high (0.5 versus 15 mM) glucose (Figures 3C and 3D). Similar results were observed for insulin secretion stimulated by cyclic AMP (cAMP)-raising reagents such as IBMX and forskolin, as well as in response to cell depolarization with KCl (Figures S4C and S4D). Furthermore, we found that the expression patterns of *STARD10* and of nearby genes was significantly altered in CBS mutated cells. qRT-PCR analyses (Figure S4E) revealed that *STARD10*, *ATG16L2*, and *FCHSD2* were the most downregulated in CBS mutant cells, while *ARAP1*, the gene that resides nearest the T2D-associated credible set, was unaffected.

These results demonstrate that CTCF binding, through its likely impact on chromatin structure organization at the *STARD10* locus, is necessary to maintain normal β cell function.

### R13 and R1 regions form chromatin loops via CBSs

CTCF, together with the Cohesin complex, plays an important role in the formation of higher-order chromatin structures and may act as an insulator or boundary between *cis*-regulatory elements and their target genes (Bell et al., 1999; Lupiáñez et al., 2015; Merkenschlager and Odom, 2013). As shown in Figure 2C, the region containing the T2D credible set in *STARD10* interacts with both R1 and R13 (and both of the latter contain bona fide CTCF convergent binding sites) (Figure 3A). We therefore hypothesized that the two regions may interact with each other, via the formation of a CTCF-CTCF loop, to establish a restricted chromatin domain. To test this hypothesis, we performed 3C analysis in EndoC-βH1 cells and explored the interaction frequency between R1 and R13. Taking R13 as the viewpoint, we detected interaction frequencies above background level across the entire *STARD10* locus (Figure 3E), particularly with the 2 promoters of *STARD10* (R3 and R1), but also with the T2D credible set region, as observed previously (Figure 2C). These observations confirm that the T2D VR interacts with a distal CTCF site in β cells and demonstrate that R13 and R1 are also associated through chromatin looping.

### Screen of annotated genomic features reveals a functional islet enhancer that regulates basal insulin secretion

Since the T2D variants are deeply embedded within the region of active enhancers (Figure 1A), we hypothesized that the causal variants may exert their effect(s) by altering the activities of these enhancers. To explore this possibility, we sought first to understand the roles of the enhancer cluster in the control of β cell gene expression and function.

**Figure 4:**
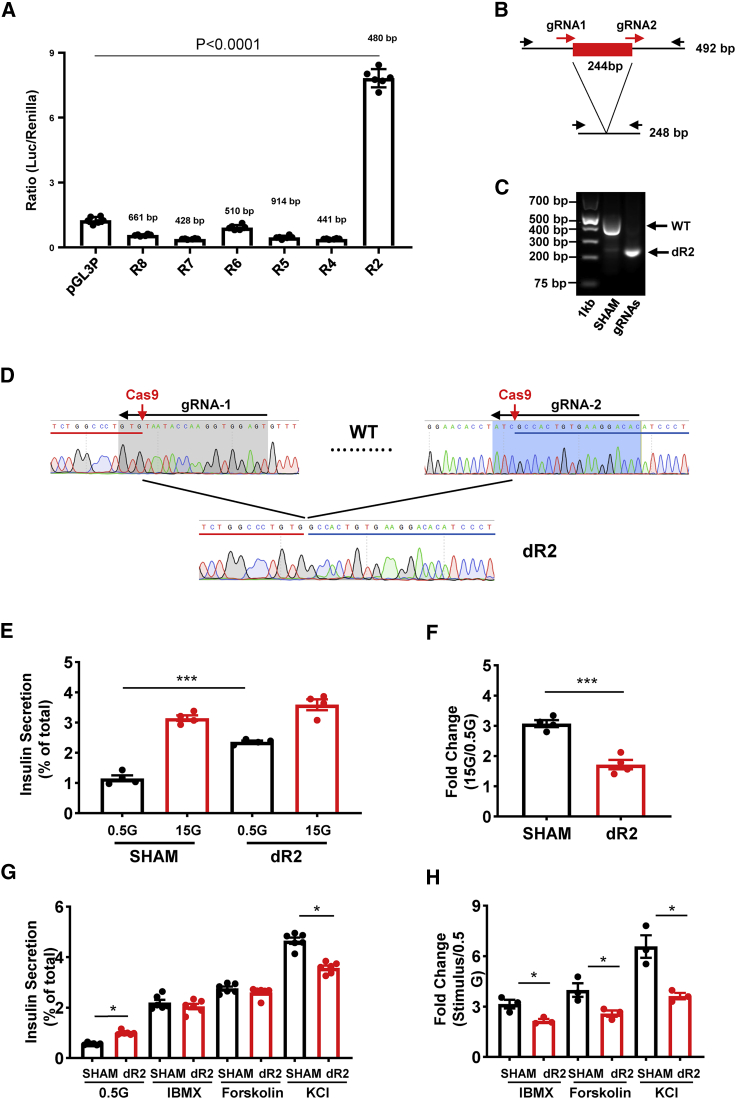
Role of enhancer R2 in insulin secretion (A) Promoter-luciferase assay in EndoC-βH1 cells; n = 3. (B) Strategy of R2 deletion by CRISPR-Cas9 genome editing. Two gRNAs were designed to delete 244 bp of the core R2 region. (C) Gel electrophoresis of PCR products amplified from genomic DNAs isolated from control and R2-deleted (dR2) cells. (D) Sanger sequencing confirmation of R2 deletion. Red and blue bars represent the 5′ and 3′ ends of the DNA sequence flanking the R2 region. (E) Representative data of GSIS assay; n = 2 (F) Fold change of secreted insulin. Data are normalized to insulin secretion at basal level (0.5 mM glucose); n = 4. (G) Representative data of insulin secretion stimulated by other stimuli; n = 3 (H) Fold change of secreted insulin. Data are normalized to insulin secretion at basal level (0.5 mM glucose); n = 3.

Regulome analyses of human islet samples, including ATAC-seq and ChIP-seq for H3K27ac, revealed 6 active enhancers showing islet TF binding (Figure 1A). We thus tested these regions by luciferase reporter assay in EndoC-βH1 cells, which revealed that R2 had a 6.25-fold activity increase compared with control (p < 0.0001) (Figure 4A). Other putative enhancers displayed negligible activity in this assay.

**Figure 5:**
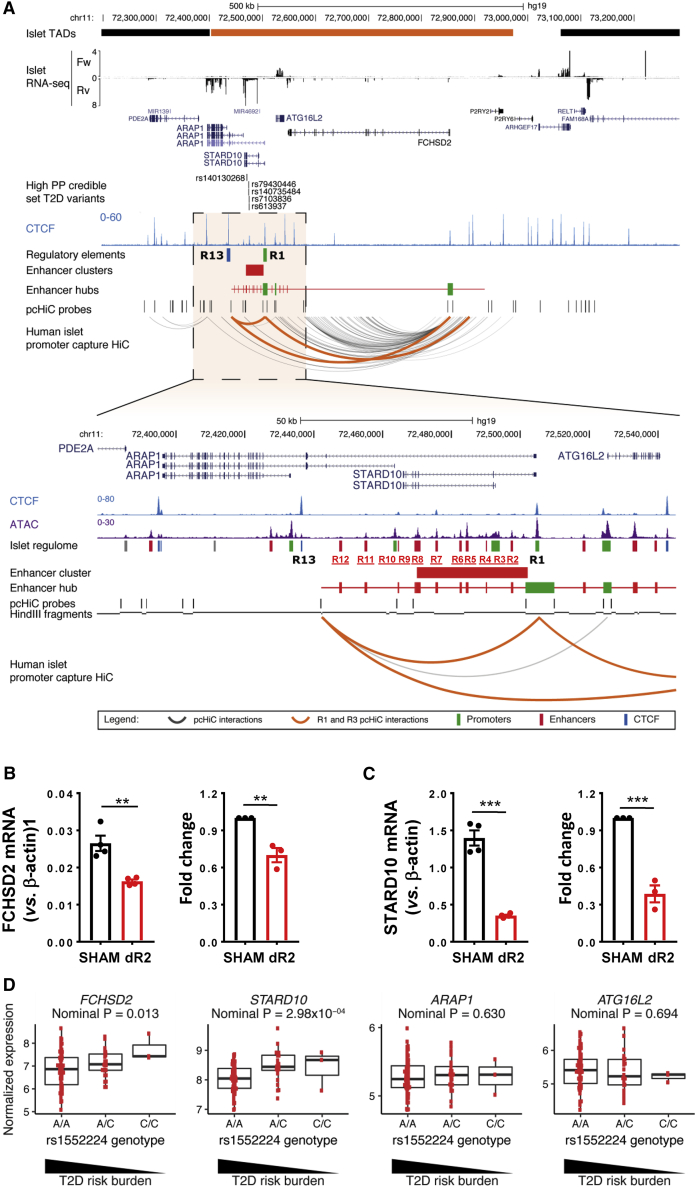
Target genes regulated by the enhancer cluster (A) Human islet promoter capture Hi-C (pcHi-C) map at *STARD10* locus and surrounding region. Orange interactions in the pcHi-C track depict interactions mediated by the CTCF sites that flank the enhancer cluster (R1 and R13). All other significant interactions are shown in dark gray. Enhancer hubs track shows all of the enhancers (red) and promoters (green) contained within the *STARD10* hub. Orange bar in islet topologically associating domains (TADs) track highlights the TAD encompassing *STARD10* and surrounding genes (*ARAP1*, *ATG16L2*, and *FCHSD2*). (B and C) Taqman qRT-PCR analysis of gene expression in SHAM control and R2-deleted (dR2) cells. (B) Representative data and fold change of *FCHSD2* gene; n = 3. (C) Representative data and fold change of *STARD10* gene; n = 3. (D) eQTL analysis of human islet samples. y axis represents normalized intensities (using robust multi-array average [RMA] method) from the Affymetrix Human Genome U133 Plus 2.0 Array; 203 total human samples.

The R2 enhancer contains several recognition sequences for the binding of islet-associated TFs, such as FOXA2 and PAX4, suggesting a critical role in the regulation of nearby β cell genes (Figure S5A). To assess the role of this enhancer in β cell function, we deleted the core region of R2 (244 bp) from EndoC-βH1 cells using CRISPR-Cas9 (Figure 4B), achieving an ~85% loss of targeted alleles (Figures 4C and 4D and S5B–S5D). GSIS was significantly impaired in edited versus sham-treated cells (p = 0.0004) (Figures 4E and 4F), largely due to increased basal insulin secretion (0.5 mM glucose) (p = 0.0004). The stimulation of insulin secretion was even more sharply reduced in R2-deleted cells in response to cAMP-raising agents or after depolarization with KCl (Figures 5G and 5H).

### Enhancer cluster regulates *FCHSD2* gene expression through chromatin looping

Individual enhancers can regulate multiple genes within the same cellular population, forming distinct 3-dimensional (3D) chromatin regulatory domains (“enhancer hubs”) (Miguel-Escalada et al., 2019; Oudelaar et al., 2018). We therefore postulated that the enhancer cluster may be part of a broader 3D chromatin domain in human islets.

To assess this possibility, we queried the recently published genome-wide map of human islet 3D chromatin interactions (promoter-capture HiC [pcHi-C]) (Miguel-Escalada et al., 2019). This analysis revealed that *STARD10* resides within an islet enhancer hub, together with *ARAP1*, *ATG16L2*, and the distal gene *FCHSD2*, located ~400 kb downstream of the *STARD10* enhancer cluster (Figure 5A). These four hub genes are expressed at relatively high to medium levels in human islets (see RNA-seq track in Figures 5A, and Figures S6A and S6B). We note, however, that *ARAP1* has three annotated promoters and one of them is included in the hub (Miguel-Escalada et al., 2019) (Figures S6A and S6B). The P1 promoter drives the expression of the longest isoform of *ARAP1* and is shared with *STARD10*. Examination of human islet RNA-seq revealed that the longer *ARAP1* transcript isoform from both P1 and P2 promoters displays much lower expression in human islets than the short isoform (Figure S4B). Thus, most *ARAP1* transcription is driven by promoter P3 (Figure S6B), which resides outside the *STARD10* islet enhancer hub.

To identify the gene(s) that are regulated by the nearby enhancer cluster, we carried out gene expression profiling in R2-deleted (dR2) cells. This revealed significant downregulation of only *FCHSD2* (p = 0.0066) and *STARD10* (p = 0.0008) (Figures 5B and 5C). In contrast, *ATG16L2* and *ARAP1*, the gene that was closest in linear distance to the enhancer cluster, were not affected by R2 deletion (Figures S6C and S6D). The latter observation is in line with our previous analysis of islet expression quantitative trait loci (eQTL), which did not reveal any association between the T2D variants in this locus and the expression of *ARAP1* (Carrat et al., 2017). Further analysis of the pcHi-C dataset confirmed that regions R1 and R13 interact in human islet chromatin (Figure 5A), consistent with our 3C analysis in EndoC-βH1 cells (Figure 3E). Moreover, the pcHi-C dataset revealed that the 2 CTCF binding sites that flank the *STARD10* enhancer cluster (R1 and R13) undergo long-range interactions with the promoter region of *FCHSD2* (Figure 5A).

### Human islet eQTL

To gain insight into the relevance of our findings in the context of human islet physiology and diabetes risk, we analyzed the expression of *FCHSD2* in a cohort of 103 subjects who provided pancreatic samples after partial pancreatectomy and laser capture microdissection analysis (IMIDIA consortium; 47 non-diabetic, 56 T2D) (Solimena et al., 2018; Marchetti et al., 2018). We observed lower *FCHSD2* expression in carriers of the risk alleles of variants (rs75896506, rs11603334, and rs1552224; nominal p = 0.013), which are in high linkage disequilibrium (LD) with the high posterior probability variant rs140130268 (EUR R^2^ = 0.89) (Figure 5D; Table S2). Consistent with earlier reports (Carrat et al., 2017; Miguel-Escalada et al., 2019), the risk variants also associated with lower *STARD10* levels in this cohort (nominal p = 2.98 × 10^−4^) (Figure 5D; Table S2). In contrast, no eQTL was found for *FCSHD2* in organ donor (OD)-obtained samples from the same cohort (p = 0.89), while the signal for *STARD10* remained nominally significant (p = 2.19 × 10^−3^). These results suggest that the T2D-associated variants in the *STARD10* enhancer hub selectively associate with differential expression of *STARD10* and *FCHSD2*.

### Deletion of *STARD10* and *FCHSD2*, but not *ARAP1*, affect regulated insulin secretion in a human β cell line

**Figure 6:**
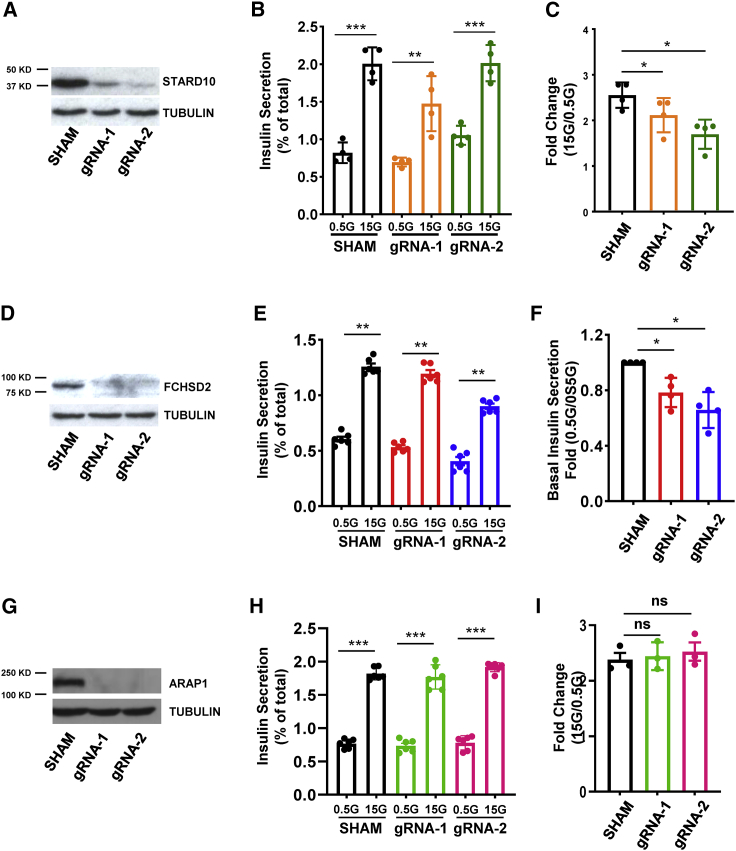
Effect of CRISPR/Cas9-mediated knockout of *STARD10*, *FCHSD2*, or *ARAP1* on basal and regulated insulin secretion (A–C) Effect of *STARD10* knockout. (A) Western blot assay. (B) Representative data of GSIS assay; n = 2. (C) Fold change of secreted insulin. Data are normalized to insulin secretion at basal level (0.5 mM glucose); n = 4. (D–F) Effect of *FCHSD2* knockout. (D) Western blot assay. (E) Representative data of GSIS assay; n = 3. (F) Fold change of secreted insulin at basal level. Data are normalized to basal insulin secretion (0.5 mM versus 0.5 mM); n = 4. (G–I) Effect of *ARAP1* knockout. (G) Western blot assay. (H) Representative data of GSIS assay; n = 3. (I) Fold change of secreted insulin. Data are normalized to insulin secretion at basal level (0.5 mM glucose); n = 3.

We have previously demonstrated the importance of the *STARD10* gene but not *ARAP1* in regulating insulin processing and secretion in the mouse (Carrat et al., 2017). The findings above suggest that *FCHSD2* may also play a role in the control of insulin secretion. To further explore the potential roles of these genes in controlling insulin secretion in the human setting, we deployed CRISPR-Cas9 gene editing in EndoC-βH1 cells to generate frameshift mutations in exon 2 of *STARD10*, exon 1 of *FCHSD2*, and exon 3 of *ARAP1* (Figures S7A–S7C). Western blot analysis confirmed the expected loss of protein expression with 80%–90% efficiency for STARD10 (Figure 6A), >95% for both FCHSD2 (Figure 6D) and ARAP1 (Figure 6G).

For STARD10 null EndoC-βH1 cells, we observed a significant reduction in glucose-stimulated insulin secretion in comparison with control (gRNA1: p = 0.007 and gRNA2: p = 0.007) (Figures 6B and 6C), which is in agreement with our previous observations in mouse models (Carrat et al., 2017). Insulin secretion at low (0.5 mM) glucose was not affected by STARD10 loss. However, FCHSD2 null cells showed a relatively mild reduction in insulin secretion at both basal (gRNA1: p = 0.0265 and gRNA2: p = 0.013) (Figures 6E and 6F) and high glucose conditions (gRNA1: p = 0.0043 and gRNA2: p = 0.028) (Figure S7D), although no significant changes were observed when comparing the high and low glucose conditions (gRNA1: p = 0.97 and gRNA2: p = 0.75) (Figure S7E). Furthermore, *ARAP1* null cells, in agreement with previous observations in a mouse model (Carrat et al., 2017), did not show any significant changes in GSIS (gRNA1: p = 0.52 and gRNA2: p = 0.75) (Figures 6H and 6I).

FCHSD2 has been shown to regulate F-actin polymerization, suggesting it could be involved in insulin exocytosis (Cao et al., 2013). To identify any impact of *FCHSD2* loss of function on late events in insulin secretion, we next explored the regulation of secretion in response to depolarization with KCl in two further lines (KO1 and KO2) deleted for *FCHSD2,* compared to control lines generated using either Sc-gRNA or a gRNA that induces double-strand DNA breaks (Figure S8A). While no differences were apparent between the control lines (Figures S8B and S8C), both of the FCHSD2 null lines demonstrated the expected lowering in both basal (0.5 mM glucose) and stimulated (15 mM glucose)-induced secretion, but no change in the fold stimulation of secretion prompted by high glucose (15 versus 0.5 mM glucose) or by KCl (Figures S8D and S8E).

We further explored regulated exocytosis in *FCHSD2*-KO lines using sensitive electrophysiological measurements of cell membrane capacitance (Figures S9A–S9E). FCHSD2 loss of function had no impact on cell size or on calcium current densities at 0 mV (Figures S9A and S9B). The cells were subjected to 10 depolarizations (pulses) from −70 to 0 mV (Figure S9C). The amplitude of exocytosis in *FCHSD2*-KO lines reached at pulse 10 was not significantly different between the lines (SHAM versus KO1 p_adjusted_ = 0.108, SHAM versus KO2 p_adjusted_ = 0.851) (Figure S9D). The cumulative increases in membrane capacitance were 10.89 ± 7.22 fF.pF^−1^ (n = 11) and 21.57 ± 3.56 fF.pF^−1^ (n = 13) for control lines and 27.29 ± 12.28 fF.pF^−1^ (n = 9) and 13.36 ± 7.48 fF.pF^−1^ (n = 12) for KO1 and KO2 lines, respectively. As observed previously (Hastoy et al., 2018), the increase in membrane capacitance was biphasic, with the majority of exocytosis elicited during the first two pulses (Figure S9E). No significant differences were observed in the increment triggered by the first pulse control and *FCHSD2*-KO lines (SHAM versus KO1 p_adjusted_ = 0.108, SHAM versus KO2 p_adjusted_ = 0.326). Thus, the increments equated to 3.36 ± 2.36 fF.pF^−1^ (n = 11) and 10.44 ± 7.04 fF.pF^−1^ (n = 13) for SHAM and Sc-gRNA lines, respectively, and to 12.10 ± 8.86 fF.pF^−1^ (n = 9) and 8.28 ± 7.21 fF.pF^−1^ (n = 12) for *FCHSD2*-KO1 and *FCHSD2*-KO2 lines, respectively. These findings argue against a direct role for FCHSD2 in controlling distal events in Ca^2+^-regulated secretory granule exocytosis.

### Deletion of the VR alters 3D chromatin structure and downregulates the expression of *STARD10* and *FCHSD2* genes

**Figure 7:**
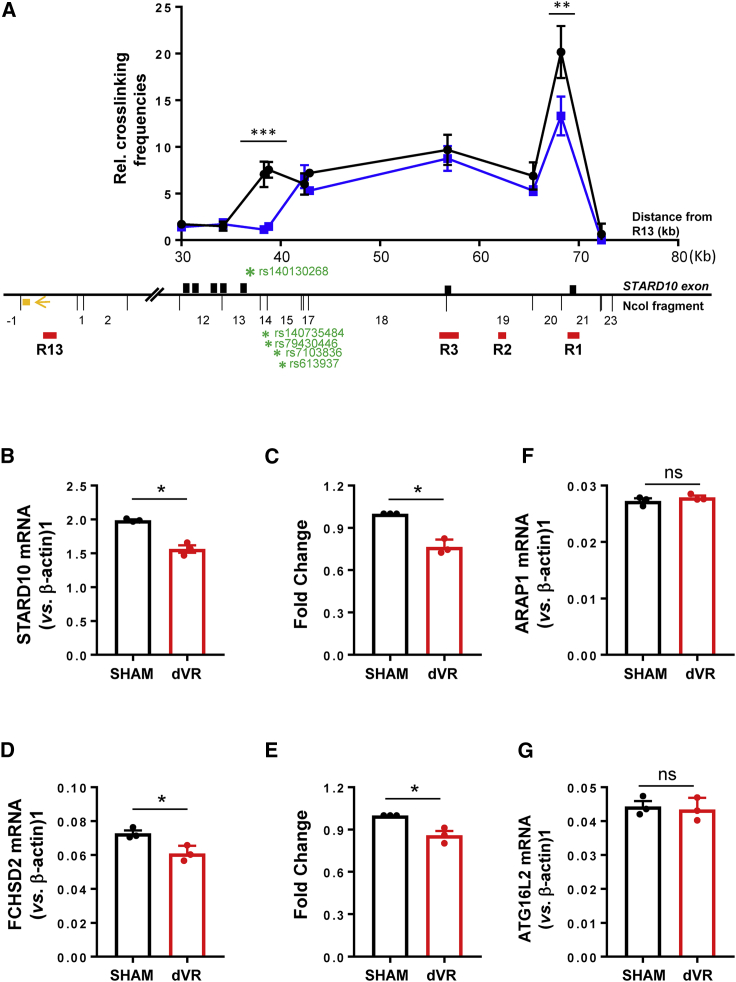
Effect of VR deletion on 3D structure and gene expression (A) Combined 3C-qPCR data of chromatin interactions in dVR cells. Black: control; blue: dVR. The numbering of *NcoI* DNA fragments is given relative to the viewpoint. Viewing point: R13 region; black bars: *STARD10* exon; red bars: regulatory region; orange box: qPCR probe; orange arrow: qPCR constant primer; green stars: credible set genetic variants. Data were normalized to a *CXCL12* loading control; n = 3. (B–G) Taqman qRT-PCR analysis of gene expression in SHAM control and dVR cells. (B and C) *STARD10*; (D and E) *FCHSD2*; (F) *ARAP1*; and (G) *ATG16L2*. (B) and (D) show relative expression levels (representative experiments); (C) and (E) indicate the fold change of expression; n = 3.

Finally, having examined the 3D structure and downstream genes of the enhancer cluster, we attempted to determine whether the risk-bearing VR may affect enhancer cluster function. As shown in Figure 1A, the VR is located between 2 active enhancers, R7 and R8, and, most important, is associated with CTCF-binding regions R13 and R1 through chromatin looping (Figure 2C). These observations suggest that the VR may be involved in the formation of local chromatin structure and thus in controlling the activity of the enhancer cluster. To test this possibility, we performed 3C-qPCR analysis in sham control and dVR cells. To increase the deletion efficiency of CRISPR-Cas9 editing in these experiments, we doubled the concentration of lentivirus (MOI = 20) and achieved ~80% deletion (Figures S10A and S10B). As shown in Figure 7A, VR deletion caused a significant change in 3D structure, notably a reduction in the physical interaction between the R13 and R1 regions (p = 0.0064). Further analysis of gene expression by qRT-PCR in dVR cells revealed that both the *STARD10* and *FCHSD2* genes were moderately downregulated when compared with sham control cells (*STARD10*: p = 0.015 and *FCHSD2*: p = 0.011). No significant change was observed in the expression of the *ARAP1* or *ATG16L2* genes (Figures 7B–7G). These data demonstrate that the VR is an important region regulating chromatin 3D structure and transcriptional activity of the enhancer cluster.

## Discussion

Our results demonstrate the involvement of STARD10 in insulin secretion in human β cells and unveil FCHSD2 as a previously unknown regulator of this process.

Our earlier study (Carrat et al., 2017) provided evidence, based on a combination of approaches, that *STARD10* is a critical mediator of the effects of the T2D-associated variants at this locus. However, our previous report did not explore in detail how the identified credible set may influence the expression of local genes, nor did it exclude the possibility that other genes may also be involved in the actions of risk variants.

The goals of the present study were, therefore, to obtain a more detailed molecular picture of the local chromatin structure at the *STARD10* T2D locus and to use this to identify and study the genes likely to interact with previously identified T2D risk variants in human β cells. In this way, we report a likely spatial organization, defined by CTCF-stabilized looping, which allows an enhancer cluster to regulate not only the *STARD10* gene but also the distal *FCHSD2* gene, which is contained in the same 3D chromatin compartment as *STARD10* and the T2D risk variants. In contrast, and consistent with our earlier studies (Carrat et al., 2017), we found no evidence for a role for *ARAP1* in mediating the effects of the variants on β cell function.

We also report here that variants of the risk haplotype are eQTLs for both *STARD10* and *FCHSD2* in human islets (note that data were not available for the indel with the highest posterior probability). More important, these results were only obtained in laser capture microdissection (LCM) donors from partially pancreatectomized patients (PPPs) from the “IMIDIA” dataset (Carrat et al., 2017). In contrast, interrogation of OD samples in the same dataset failed to reveal a nominal association between any of the T2D risk SNPs and *FCHSD2* expression. Moreover, interrogation of the other islet eQTL datasets reported earlier, which are also derived from OD samples (Fadista et al., 2014; Miguel-Escalada et al., 2019), provided no evidence of association with islet *FCHSD2* levels.

We note that the use of LCM to extract mRNA is unlike the enzymatic digestion methods that are commonly used with OD samples. Thus, surgical specimens are subject to immediate cryo-fixation, limiting RNA degradation and hence transcriptomic changes, which can occur in OD samples in which islets are isolated from the rest of the pancreas before mRNA extraction. Moreover, LCM methods provide a purer and more β cell-enriched cell population (Solimena et al., 2018). Correspondingly, OD and LCM samples from the same individual cluster separately (Solimena et al., 2018).

We suspect that the use of this dataset (Solimena et al., 2018) was critical to revealing an association between T2D risk variants at the *STARD10* locus, and the levels of expression of the *FCHSD2* gene. We note that this eQTL only achieved nominal association in a targeted analysis of the locus, but did not reach genome-wide significance, and that the additional human islet eQTL datasets we interrogated in this study (Fadista et al., 2014; Miguel-Escalada et al., 2019) only reported genome-wide significant eQTLs. These additional islet eQTL datasets were derived from OD samples. It is possible, therefore, that β cell-specific effects may be masked by “contaminating” signals from islet non-β and other cells. Supporting this view, interrogation of OD data from both the IMIDIA dataset (Solimena et al., 2018) and datasets from the Parker (Varshney et al., 2017) and Groop (Fadista et al., 2014) labs, and from Groop and Ferrer combined (Miguel-Escalada et al., 2019), as well as more recent OD data (Viñuela et al., 2020), fail to reveal a nominal association between SNP rs1552224 and *FCHSD2* expression (based on the index SNP). Interrogation of a very recent dataset involving 26 normoglycemic subjects in which islets were isolated from ODs and, subsequent to islet dispersal, purified by fluorescence-activated cell sorting (FACS) (Viñuela et al., 2020), demonstrated nominal eQTLs for rs1552224 and *STARD10* (7/7 exons), *PDE2A* (15/35 exons), *ARAP1* (1/9 exons), and *FAM168A* (1/9 exons) as well as *FCHSD2* (2/22 exons) at borderline (p = 0.05) significance or non-significance (p = 0.06). These data may further support the view that enhanced β cell purity contributes to the detection of an eQTL for *FCHSD2* in the LCM, but not OD (whole islet), data.

We used a CRISPR-Cas9 approach to delete the whole of the 4.1-kb VR likely to host the credible set of T2D variants with significant posterior probability at this locus (Carrat et al., 2017). This region was also identified as a credible set in earlier reports (Bonàs-Guarch et al., 2018; Gaulton et al., 2015; Mahajan et al., 2018), but did not feature in a recent report (Mahajan et al., 2018). Unlike our previous analysis, the new report from Mahajan et al. (2018) does not include indels (a Haplotype Reference Consortium [HRC] panel was used that included only SNPs). Thus, the variant in the VR with the highest PPA (indel rs140130268), as described in our earlier report (Carrat et al., 2017) in which a Metabochip scaffold was imputed up to a 1000 Genomes reference panel including indels, was not included. Whereas indel rs140130268 showed a PPA of 45% (Carrat et al., 2017), in Mahajan et al. (2018), maximum PPA is assigned to rs7109575 (PPAg [genetic credible set] = 0.14 [14%] and PPAf [functional credible set] = 0.38 [38%]). These two SNPs are in very high LD (EUR R^2^ = 0.95). In any case, the discordance between earlier studies emphasizes the need for direct interventional studies in disease-relevant cells, as presented here.

While the ideal design of such studies would involve using homologous recombination to convert risk into protective alleles (or vice versa) one by one, currently available cellular systems largely preclude this. EndoC-βH1 cells grow slowly and do not tolerate single cell cloning, while human embryonic stem cell (ESC)-derived β cells (Nair et al., 2019; Pagliuca et al., 2014; Rezania et al., 2014) do not reliably provide a robust platform for functional studies. Although the approach adopted here to delete the whole 4.1-kb region in EndoC-βH1 cells provides no information on the role of the individual variants, it does allow us to suggest that this region is important for the control of local gene expression and β cell function.

### Enhancer hub and chromatin structure at the *STARD10* locus

The spatial organization of chromatin can play an important role in gene regulation (Vernimmen and Bickmore, 2015). We found that the T2D risk variants at the *STARD10* locus reside in an islet enhancer hub that contains the genes *ARAP1*, *STARD10*, *ATG16L2*, and *FCHSD2*. Of note, the expression of *ARAP1* in human islets is chiefly driven by two promoters that reside outside the enhancer hub (Figures 5A and S4C) and is thus not likely to be co-regulated with *STARD10* and other hub genes, in line with evidence from eQTL studies (this report and Carrat et al., 2017).

Of note, we demonstrate that two CBSs exist at either end of the cluster, possibly creating a spatially organized transcriptional complex that is likely to influence the expression of relevant genes (Figure 1A).

### *STARD10* and *FCHSD2* mediate altered β cell function and disease risk

Our previous study (Carrat et al., 2017) provided evidence of an essential role for *STARD10* in mediating glucose-induced insulin secretion and proinsulin processing in the mouse. Here, using CRISPR-Cas9-mediated inactivation, we demonstrate that STARD10 is equally important in human β cells. In contrast, deletion of *ARAP1* from human EndoC-βH1 cells had no effect on basal or glucose-stimulated secretion, in line with our earlier study in the mouse (Carrat et al., 2017) and further arguing against a role for this gene in mediating the effects of risk genes at this locus. In addition, we failed to obtain any evidence for an impact of the disease-associated variants on *ARAP1* expression in human islets. We note, however, that the Affymetrix probes used (U133, Plus 2.0) detect exons common to all isoforms, including the low-abundance long transcript expressed from the P1 in the enhancer hub, as well as from promoter P2 (Figures 5A, S4C, and S4D). We are therefore unable to exclude the possibility that this transcript may be affected by the variants, although the impact, if any, of such a change is unclear.

Although the molecular roles for STARD10 in the β cell remain to be elucidated, our recent findings (Carrat et al., 2020) indicate a role in the control of secretory granule biogenesis. Analysis of human islet pcHiC (Miguel-Escalada et al., 2019) and measurements of changes in gene expression after deletion of R2 region or the core of the VR provide evidence that *FCHSD2* may also play a role in mediating disease risk. Interestingly, deletion of the VR led to a lowering of stimulated insulin secretion, but no change in basal insulin secretion (Figures 1F and 1G). Whereas the knockout of *FCSHD2* lowered both basal and stimulated insulin release (Figures 6H and S7D), the loss of *STARD10* had no effect on insulin secretion at low glucose, but it did impair insulin secretion at high glucose (Figures 6C and 6D). It may, therefore, be speculated that the VR exerts effects on insulin secretion via a combined action on both genes.

By what mechanisms might FCHSD2 affect insulin secretion? FCHSD2 has been shown to regulate F-actin polymerization (Cao et al., 2013) and act as a positive regulator of clathrin-mediated endocytosis (Almeida-Souza et al., 2018). More important, impaired clathrin-mediated endocytosis, achieved by the inactivation of dynamin-2 in β cells, impairs insulin exocytosis (Fan et al., 2015).

FCHSD2 is recruited to clathrin-coated pits by interaction through its second HS3 domain (SH3-2), while its first SH3 domain (SH3-1) binds to N-WASP to initiate F-actin polymerization (Almeida-Souza et al., 2018; Xiao et al., 2018). Exocytosis requires F-actin-mediated cytoskeletal remodeling (Jewell et al., 2008; Kalwat and Thurmond, 2013) in which N-WASP and the ARP2/3 complex are important protein complexes in the formation of focal adhesion. By affecting these processes, FCHSD2 may play an active role in the regulation of late events in secretion. However, measurements of exocytosis using sensitive capacitance recordings (Figures S9A–S9E) failed to provide direct evidence for such a role. Instead, we speculate that FCHSD2 regulates more proximal events, such as the recruitment of granules to a “reserve” pool (Rorsman and Renström, 2003) or the recycling of receptors (e.g., those for glucagon-like peptide-1 [GLP1]) (Jones et al., 2018).

### Possible impact of T2D variants on chromatin landscape at the *STARD10* locus

We show that the VR region is located between two enhancers (R7 and R8) that are occupied by β cell-specific TFs such as PDX1 (Figure 1A). We also demonstrate that the VR interacts with CBSs and that its deletion causes a significant change in 3D structure, affecting the expression of the downstream genes *STARD10* and *FCHSD2* (Figure 7).

We also reveal that deleting a genomic region hosting variants associated with T2D affects chromatin structure. Future studies, in more tractable systems (e.g., CRISPR-edited human embryonic stem cell-derived β-like cells) are likely to be required to determine the effect of more targeted (e.g., single variant, haplotype) changes on local DNA structure.

In summary, the present report extends our previous study of the *STARD10* locus (Carrat et al., 2017), revealing important aspects of the control of the local chromatin structure and identifying a functionally relevant new gene, *FCHSD2*.

## STAR★methods

### KEY RESOURCES TABLE

**Table:** 

REAGENT or RESOURCE	SOURCE	IDENTIFIER
**Antibodies**		

CTCF	EMD Millipore	Cat# 07-729; RRID: AB_441965
Rabbit IgG, plasma	EMD Millipore	Cat# 401590; RRID: N/A
STARD10	Santa Cruz Biotechnology	Cat# sc-54336; RRID: AB_2197780
FCHSD2	Almeida-Souza et al., 2018	N/A
ARAP1	Abcam	Cat# ab99382; RRID: AB_10675661
α-TUBULIN	Sigma-Aldrich	Cat# T5168; RRID: AB_477579)

**Bacteria and virus strain**		

Stbl3 competent cells	Thermo Fisher Scientific	Cat# C737303
10-beta competent cells	New England Biolabs	Cat# C30191

**Biological samples**		

Human islet samples	Carrat et al., 2017	N/A

**Chemicals, peptides, and recombinant proteins**		

3-Isobutyl-1-methylxanthine (IBMX)	Sigma-Aldrich	Cat# I5879
Foskolin	Sigma-Aldrich	Cat# F3917
Sodium Selenite	Sigma-Aldrich	Cat# S1382
Nicotinamide	Sigma-Aldrich	Cat# 481907
Paraformaldehyde	Agar Scientific	Cat# AGR1026
TRIzol	Invitrogen	Cat# 15596018
NP40	Sigma-Aldrich	Cat# 13021
Proteinase inhibitor cocktail	Sigma-Aldrich	Cat# 04693132001
Albumin from Bovine Serum Fraction V	Roche Diagnostics	Cat# 10775835001
Human Transferrin	Sigma-Aldrich	Cat# T8158
ECM	Sigma-Aldrich	Cat# E1270
RNase A	Thermo Fisher Scientific	Cat# EN0531
Proteinase K	Sigma-Aldrich	Cat# AM2546
Dynabeads-Protein G	Thermo Fisher Scientific	Cat# 10003D
Dynabeads-Protein A	Thermo Fisher Scientific	Cat# 10008D
ATP, [γ-^32^P]	Perkin Elmer	Cat# NEG002A

**Critical commercial assay**		

Dual-Luciferase Assay Reporter Assay System	Promega	Cat# E1910
Lipofectamine 2000	Thermos Fisher Scientific	Cat# 11668025
Insulin Ultra-sensitive Assay Kit	Cisbio Bioassays	Cat# 62IN2PEH
High-Capacity cDNA Reverse Transcription Kit	Thermos Fisher Scientific	Cat# 4368814
NE-PER™ Nuclear and Cytoplasmic extraction reagents	Thermos Fisher Scientific	Cat# 78833
Taqman™ Fast advanced master mix	Thermos Fisher Scientific	Cat# 4444553
Fast SYBR™ Green Master Mix	Thermos Fisher Scientific	Cat# 4385612
Phusion High fidelity DNA polymerase	Thermos Fisher Scientific	Cat# F530
T4 polynucleotide kinase	New England BioLabs	Cat# M0201S

**Experimental model: cell line**		

HEK293T	ATCC	Cat# CRL_3216; RRID: CVCL_0063
EndoC-βH1	Univercell-Biosolutions	Cat# N/A; RRID: CVCL_L909

**Oligonucleotides**		

VR_KO1: GACCCCTGTGAGCTCCTCGT	This paper	N/A
VR_KO2: AGCGACCACCAGCTAGGTTT	This paper	N/A
CBS1: GCTTGGGTGGGGGTGCAGCC	This paper	N/A
CBS2: GGGCAGTCAAGGGCACAGGA	This paper	N/A
CBS5: TGCAGAAGAATGGTCACTAG	This paper	N/A
CBS7: AGTCTCGGGATCGACACGTG	This paper	N/A
CBS8: CGGCCACAACCACTAGGGGG	This paper	N/A
R2_KO1: GTGTCCTTCACAGTGGCGAT	This paper	N/A
R2_KO2: ACTCCACCTTGGTATTACAC	This paper	N/A
STARD10-1: AGAGGCCGCCAGCTTCTCCA	This paper	N/A
STARD10-2: AGTCTTGGTCATCGGGCACC	This paper	N/A
FCHSD2-1: GCATCATGCAGCCGCCGCCG	This paper	N/A
FCHSD2-2: GCCCTTACCTTCCTCGGCGG	This paper	N/A
ARAP1-1: TGGGGATGCTGCGCTATCGG	This paper	N/A
ARAP1-2: TGCACCTGGAGCAGTACACG	This paper	N/A
HBB_KO1: GCACCATAAGGGACATGATA	This paper	N/A
HBB_KO2: TTTCCTTACTAAACCGACAT	This paper	N/A
Inter_KO1: TTGAGGTTACATCATTCTAC	This paper	N/A
Inter_KO2: AGTACCTAAAGAGACCCACA	This paper	N/A
Scrabmble_1: GAACTCAACCAGAGGGCCAA	This paper	N/A
Scrabmble_2: GGGAGGTGGCTTTAGGTTTT	This paper	N/A

**Recombinant DNA**		

pMD2.G	Didier Trono (https://www.epfl.ch/labs/tronolab/)	Cat# 12259; RRID: Addgene_12259
pSPAX2	Didier Trono (https://www.epfl.ch/labs/tronolab/)	Cat# 12260; RRID: Addgene_12260
BAC DNA	bacpacresources.org	RP11-101P7
pLenti-CRISPR-RIP-Cas9	Paul Gadue	N/A
pGL3-promoter	Promega	E1761
pRL-Renilla	Promega	E2231
pBlueScript II KS+	Stratagene/Agilent	212207

**Software**		

GraphPad Prism 7	GraphPad Software	RRID: SCR_002798
Illustrator	Adobe	RRID:SCR_010279
Photoshop	Adobe	RRID: SCR_014199

### Resource availability

#### Lead contact

Further information and requests for resources and regents should be directed to and will be fulfilled by the lead Contact, Guy A Rutter (g.rutter@imperial.ac.uk)

#### Materials availability

All unique/stable reagents generated in this study are available from the Lead Contact without restriction

#### Data and code availability

This study did not generate any unique datasets or code.

### Experimental model and subject details

The human-derived β cell line EndoC-βH1 was grown on ECM (1% v/v) and Fibronectin (2 μg/ml)-coated plates or Petri dishes in serum-free DMEM containing low glucose (1 g/L), 2% (w/v) albumin from bovine serum fraction V, 50 μM β-Mercaptoethanol, 10 mM nicotinamide, 5.5 μg/mL human transferrin, 6.7 ng/mL sodium selenite, penicillin (100 units/mL), and streptomycin (100 μg/mL) (Ravassard et al., 2011). HEK293T cell was cultured in DMEM high glucose medium supplemented with 10% fetal bovine serum, 6 mM L-glutamine, penicillin (100 μg/mL) and streptomycin (100 μg/mL).

### Method details

#### Electrophoretic mobility shift assay (EMSA)

Electrophoretic mobility shift assay (EMSA) was carried out as previously described (Stewart et al., 1997). In brief, complementary oligonucleotides were designed to contain either risk or protective variants (Table S3). EndoC-βH1 nuclear extract was prepared using NE-PER nuclear and cytoplasmic extraction kit according to manufacturer’s instruction (Thermo Scientific). Oligonucleotides bearing either risk or protective variants were synthesized (Sigma) and end-labeled with γ-^32^P-ATP using T4 polynucleotide kinase (New England BioLabs). ^32^P-labeled oligoes were incubated for 20 min at room temperature with 5 μg of nuclear extract in binding reactions consisted of 1 x binding buffer (20 mM HEPES pH 7.9, 90 mM KCl, 5 mM MgCl_2_, and 0.05% Nonidet P-40) and 1 μg poly(dI-dC). Samples were electrophoresed on a 5% acrylamide gel in 0.5 x TBE buffer (90 mM Tris, 64.6 mM Boric acid and 2.5 mM EDTA, pH 8.3). The acrylamide gel was then vacuum-dried and autoradiographed.

#### Chromatin conformation capture (3C and 4C)

3C was performed as described (Hagège et al., 2007). In brief, a suspension of 1 × 10^7^ EndoC-βH1 cells was cross-linked with 4% (v/v) formaldehyde at room temperature for 10 min. The cross-linked DNA was digested overnight with restriction enzyme *Nco*I and then ligated with T4 DNA ligase at 16°C overnight. The ligated 3C DNA was purified by extraction with phenol/chloroform and precipitation with ethanol. The ligation products were quantitated by Taqman™ qPCR and normalized to the human *CXCL12* gene. The standard curve for each primer pair was generated using *Nco*I-digested and ligated BAC DNA (RP11-101P7) encompassing the human *ARAP1*, *STARD10*, and *ATG16L2* loci. The Taqman™ probes and primers used for the 3C experiments presented in this study are listed in Table S3. Taqman™ qPCR were carried out on a 7500 Fast Real-Time PCR System (Applied Biosystems). PCR reactions were set as follows: 95°C for 10 min., then with 45 cycles at 95°C 30 s and 58°C 45 s. Crosslinking frequencies were plotted as percentage of that of the human *CXCL12* gene. BamHI-digested and ligated 3C sample was used as a negative control.

For 4C, 1 × 10^7^ EndoC-βH1 cells were fixed with 4% paraformaldehyde, digested with restriction enzyme PstI, ligated with T4 DNA ligase and then digested with second restriction enzyme *Msp*I. After the second round of DNA ligation with T4 DNA ligase, DNAs were purified with phenol/chloroform extraction and ethanol precipitation. PCR reactions were carried out to amplify ligation products using nested PCR primer sets (Table S3). PCR products were then digested with restriction enzymes *Xho*I and *Not*I and sub-cloned into pBluescript II KS+ (pBSKS) (Stratagene/Agilent) for Sanger sequencing analysis.

#### Chromatin immunoprecipitation

Immunoprecipitation was carried out according to a standard protocol (Boj et al., 2001). In brief, 1 × 10^6^ EndoC-βH1 cells were fixed with 1% (v/v) formaldehyde for 10 min. and quenched with 1.25 mM glycine. Cells were then scraped and resuspended in lysis buffer (2% Triton-100, 1% SDS, 100 mM NaCl, 10 mM Tris-HCl, 1 mM EDTA). After 20 stokes of homogenization with a disposable pestle, cells were sonicated for 10 min. using Covaris™ S220 to breakdown genomic DNA to 200-500 bp fragments. DNA/protein complexes were then precipitated with anti-CTCF antibody or rabbit IgG (EMD Millipore) conjugated with protein A and G beads. DNAs were purified through Phenol/Chloroform extraction and Ethanol precipitation.

#### PCR and qPCR

Fusion high fidelity Taq polymerase (Thermo Fisher Scientific) was used in all routine PCR reactions to avoid PCR errors. A typical PCR reaction was set as follow: 98°C for 30 s, then with 35 cycles at 98°C 10 s, 60°C 10 s and 72°C 15 s. The primer sets for genomic DNA amplification are listed in Table S3.

Total RNA from EndoC-βH1 cells was obtained using TRIzol reagent (Invitrogen). Total RNAs (2 μg) were then reverse-transcribed into first strand cDNAs using High-Capacity cDNA Reverse Transcription Kit (Thermo Fisher Scientific) according to the manufacturer’s instructions. Real-time PCR was performed on a 7500 Fast Real-Time PCR System using the Fast SYBR™ Green master mix or Fast Taqman™ master mix. The SYBR™ Green PCR primer sets for variant region and CTCF binding site (CBS) are listed in Table S2. The experiment was performed in duplicate and repeated three times.

#### Molecular cloning

Active enhancer regions, identified by integration of previously published human islet ATAC-seq and H3K27ac ChIP-seq datasets (Miguel-Escalada et al., 2019), were PCR-amplified from BAC DNA (RP11-101P7) with primer sets (Table S3) designed by Primer3-based software and cloned into pGL3-promoter vector between NheI and XhoI restriction enzyme sites. Plasmid DNA was extracted using mini-prep plasmid extraction kit and/or Maxi-prep plasmid extraction kit (QIAGEN). Correct cloning was confirmed by Sanger sequencing.

#### Transfection and luciferase assay

EndoC-βH1 cells were seeded at a density of 50,000 per well in 48-well plates. After 48 hours, 0.4 μg of luciferase constructs containing putative regulatory sequences were co-transfected with 1 ng of pRL-Renilla construct as internal control into EndoC-βH1 cells, using Lipofectamine 2000, according to manufacturer’s instruction. pGL3-promoter vector was served as a negative control. 48 h later, transfected cells were washed once with PBS and lysed directly in passive cell lysis buffer (Promega). Cells were incubated on a rotating platform at room temperature for 10 min. to ensure complete lysis of cells, and then spun at 10,000 rpm for 10 min to remove cell debris. Supernatant was transferred into a fresh tube and used to measure luciferase activity with Dual-Luciferase Reporter Assay kit (Promega) on a Lumat LB9507 luminometer (Berthold Technologies). Firefly luciferase measurements were normalized to *Renilla* luciferase.

#### CRISPR-Cas9-mediated genome editing

gRNA sequences were designed using the software provided by Broad Institute (https://portals.broadinstitute.org/gpp/public/analysis-tools/sgrna-design) (Doench et al., 2016). To generate mutations or deletions in EndoC-βH1 cells, lentiviral constructs carrying both gRNA and humanized *S. pyogenes* Cas9 (*hsp*Cas9) were transfected into HEK293T cells together with packaging plasmids PMD2.G and psPAX2 using CaCl_2_ transfection protocol (Graham and van der Eb, 1973). The lentiviral vector containing RIP-Cas9 gene cassette but without gRNA was served as a SHAM control. Next day, cells were treated with sodium butyrate (10 mM) for 8 hours before changing to fresh medium. The medium was collected twice in the next two days and subjected to ultracentrifugation (Optima XPN-100 Ultracentrifuge, Beckman Coulter) at 26,000 rpm for 2 hours at 4°C. The lentiviruses were collected from the bottom of the tube and titrated. Same amount of viruses was used to transduce to EndoC-βH1 cells (MOI = 10). Puromycin (4 μg/ml) was added 72 h after infection to select lentivirus-infected cells. For deletion of genomic regions, two plasmids carrying two different gRNAs flanking target regions were co-transfected into HEK293T cells with packaging plasmids. All sequences of gRNAs and primers used for genotyping of genome editing experiments in this study are listed in STAR methods and Table S3, respectively.

To measure deletion efficiency after CRISPR/Cas9 mediated genome editing, Cyber Green qPCR was deployed to detect wild-type allele using primers 1 and 2 from genomic DNA extracted from control and DNA deletedcells. *CXCL12* was served as an internal DNA copy number control (Key resources table). The deletion efficiency was calculated as: [1-2^ΔΔC*t*(del-*CXCL12*)^/2^ΔΔC*t*(WT-*CXCL12*)^] x 100%. In addition, the relative values of DNA deletion or inversion was also measured using primer set 1+4 or 1+3 respectively. The primers were listed in Table S3.

#### Electrophysiology

Membrane capacitance measurements were performed at 32°C in standard whole cell configuration. The recordings were performed using an EPC-10 amplifier and Pulse software. In brief, the extracellular medium was composed of: 118 mM NaCl, 5.6 mM KCl, 2.6 mM CaCl_2_, 1.2 mM MgCl_2_, 5 mM HEPES, and 20 mM tetraethylammonium (TEA) (pH 7.4 with NaOH). The intracellular medium comprised: 129 mM CsOH, 125 mM Glutamic acid, 20 mM CsCl, 15 mM NaCl, 1 mM MgCl_2_, 0.05 mM EGTA, 3 mM ATP, 0.1 mM cAMP, 5 mM HEPES (pH7.2 with CsOH). Exocytosis was detected as changes in cell membrane capacitance while the cells were subjected to ten depolarisations (pulse) of 500ms from −70mV to 0mV at 1Hz. For each cell, the size is determined by the membrane capacitance value measured before the first pulse and the calcium current measured from −40mV to +40mV is triggered by a 100ms depolarisation from the resting potential (−70mV). For each recording, the amplitude of the calcium current corresponds to the average of the second component of the inward current in order to avoid the impact of the sodium current. Data were analyzed using R software with ggstatsplot (https://CRAN.R-project.org/package=ggstatsplot) and plotted in Origin Pro 2020 software. The Analysis of Variance was performed using Welch’s ANOVA followed by Games-Howell post hoc test and p values adjusted by the Benjamini & Hochberg procedure.

#### Insulin secretion

EndoC-βH1 cells were seeded onto ECM/Fibronectin-coated 48-well plates at 2.5 x 10^5^ cells per well. Four days after seeding, cells were incubated overnight in a glucose starving medium (glucose-free DMEM supplemented with 2% Albumin from bovine serum fraction V, 50 μL 2-mercaptoethanol, 10 mM nicotinamide, 5.5 μg/ml transferrin, 6.7 ng/ml sodium selenite, 100 units/ml, penicillin, 100 μg/ml streptomycin and 2.8 mM glucose).

The next morning cells were incubated for 1 h in Krebs-Ringer solution [0.2% BSA, 25% solution 1 (460 mM NaCl), 25% solution II (96 mM NaHCO_3_, 20 mM KCl and 4 mM MgCl_2_), 25% solution III (4 mM CaCl_2_), 10 mM HEPES] supplemented with 0.5 mM glucose. EndoC-βH1 cells were then incubated in the presence of low (0.5 mM) or high glucose (15 mM) or other stimuli [0.5 mM IBMX or 20 nM Forskolin or 20 mM KCl]. After incubation for 1 h, the supernatant was collected, placed onto ice and centrifuged for 5 min. at 3,000 rpm at 4°C. The supernatant was then transferred into a fresh tube. Cells were lysed in 50 μL of cell lysis solution (TETG: 20 mM Tris pH 8.0, 1% Triton X-100, 10% glycerol, 137 mM NaCl, 2 mM EGTA). The lysate was then removed to a fresh tube and centrifuged at 3,000 rpm for 5 min at 4°C. Insulin content was measured using an insulin ultra-sensitive assay kit. Secreted insulin was normalized as percentage of total insulin content. Fold increase in glucose- or other stimuli-stimulated insulin secretion is expressed as a ratio in comparison with secretion at basal level (0.5 mM glucose). Insulin secretion assays were performed in either duplicate or triplicate with insulin measurement in duplicates, as indicated.

#### Human islet regulome and interactome analysis

Human islet regulome maps, including accessible chromatin regions (ATAC-seq) and ChIP-seq for H3K27ac, CTCF and different islet-enriched TFs (NKX2.2, FOXA2, PDX1 and MAFB) were obtained from previously published datasets (Pasquali et al., 2014; Miguel-Escalada et al., 2019) and visualized using the UCSC Genome Browser (http://genome.ucsc.edu/) with GRCh37/hg19 assembly. Represented data corresponds to consolidated tracks released by Miguel-Escalada et al. (2019). Details on number of samples per track are available (Miguel-Escalada et al., 2019). Previously published human islet RNA-seq (Morán et al., 2012), chromatin interaction maps (Miguel-Escalada et al., 2019) were visualized on the WashU Epigenome browser using this session link: http://epigenomegateway.wustl.edu/browser/?genome=hg19&session=62hGf7nfcS&statusId=140947077. For pcHi-C interactions, only interactions with a CHiCAGO score > 5 were taken as high-confidence interactions, as previously described (Miguel-Escalada et al., 2019). For visualization of purposes only, pcHi-C interactions mediated by the CTCF sites R1 and R13 were colored in orange and all other high-confidence interactions in gray (Figure 5A).

#### Transcription factor binding motif analysis

TF binding profile on genetic variants was carried out using JASPAR CORE program (http://jaspar.genereg.net) (Khan et al., 2018). The threshold of relative profile score was set up at 80%. The scores of potential transcription factors were compared between risk and protective variants and listed in Table S1.

#### Expression quantitative trait loci (eQTL) analysis

Pancreatic tissues and blood samples were collected from 103 patients that have undergone partial pancreatectomy from the IMIDIA consortium (Khamis et al., 2019; Solimena et al., 2018), with appropriate permissions from donors and/or families. Briefly, expression data was acquired from islets isolated by laser capture microdissection from surgical specimens using Human Genome U133 Plus2.0 Array (Affymetrix). DNA was genotyped using the 2.5 M Omniarray beadchip (Illumina) and imputed with 1000 Genomes reference panel (phase 3), resulting in 7.5 M SNPs. Standard quality control assessment was carried out on the genotyping data using PLINK (Purcell et al., 2007). Expression and genotype analysis was combined to generate eQTLs, performed with FastQTL (Ongen et al., 2016) with gender and age as covariates. A cis-window of 500 kb was used, i.e., the maximum distance at which a gene-SNP is considered local.

### Quantification and statistical analysis

Data are expressed as means ± SEM. Significance was tested by Student’s two-tailed t test, Mann-Whitney test for non-parametric data, and one- or two-way ANOVA with SIDAK multiple comparison test, as appropriate, using Graphpad Prism 7.0 software. p < 0.05 was considered significant. The statistical details can be found in Method details and in the figure legends (indicated as *n* number).a

## Supplemental information

Document S1. Figures S1–S10 and Tables S1–S3

Document S2. Article plus supplemental information
