# Chromatin 3D interaction analysis of the *STARD10* locus unveils *FCHSD2* as a regulator of insulin secretion

**DOI:** 10.1016/j.celrep.2021.108881

**Published:** 2021-03-16

**Authors:** Ming Hu, Inês Cebola, Gaelle Carrat, Shuying Jiang, Sameena Nawaz, Amna Khamis, Mickaël Canouil, Philippe Froguel, Anke Schulte, Michele Solimena, Mark Ibberson, Piero Marchetti, Fabian L. Cardenas-Diaz, Paul J. Gadue, Benoit Hastoy, Leonardo Almeida-Souza, Harvey McMahon, Guy A. Rutter

(Cell Reports *34*, 108703-1–15.e1–e5; February 2, 2021)

In the originally published version of this article, author name Leonardo Almeida-Souza was misspelled in the author list. The corrected author name now appears here and with the paper online.

The authors regret this error.

