# Supplementary material for: Chromatin 3D interaction analysis of the STARD10 locus unveils FCHSD2 as a regulator of insulin secretion
Source: Cell Rep. 2021 Feb 2;34(5):108703. doi: 10.1016/j.celrep.2021.108703 (PMC7856552; doi:10.1016/j.celrep.2021.108703)
Supplement: Document S1. Figures S1–S10 and Tables S1–S3 [file mmc1.pdf]

Cell Reports, Volume *34*

Supplemental Information

Chromatin 3D interaction analysis of the *STARD10* locus unveils *FCHSD2* as a regulator of insulin secretion

Ming Hu, Inês Cebola, Gaelle Carrat, Shuying Jiang, Sameena Nawaz, Amna Khamis, Mickaël Canouil, Philippe Froguel, Anke Schulte, Michele Solimena, Mark Ibberson, Piero Marchetti, Fabian L. Cardenas-Diaz, Paul J. Gadue, Benoit Hastoy, Leonardo Alemeida-Souza, Harvey McMahon, and Guy A. Rutter

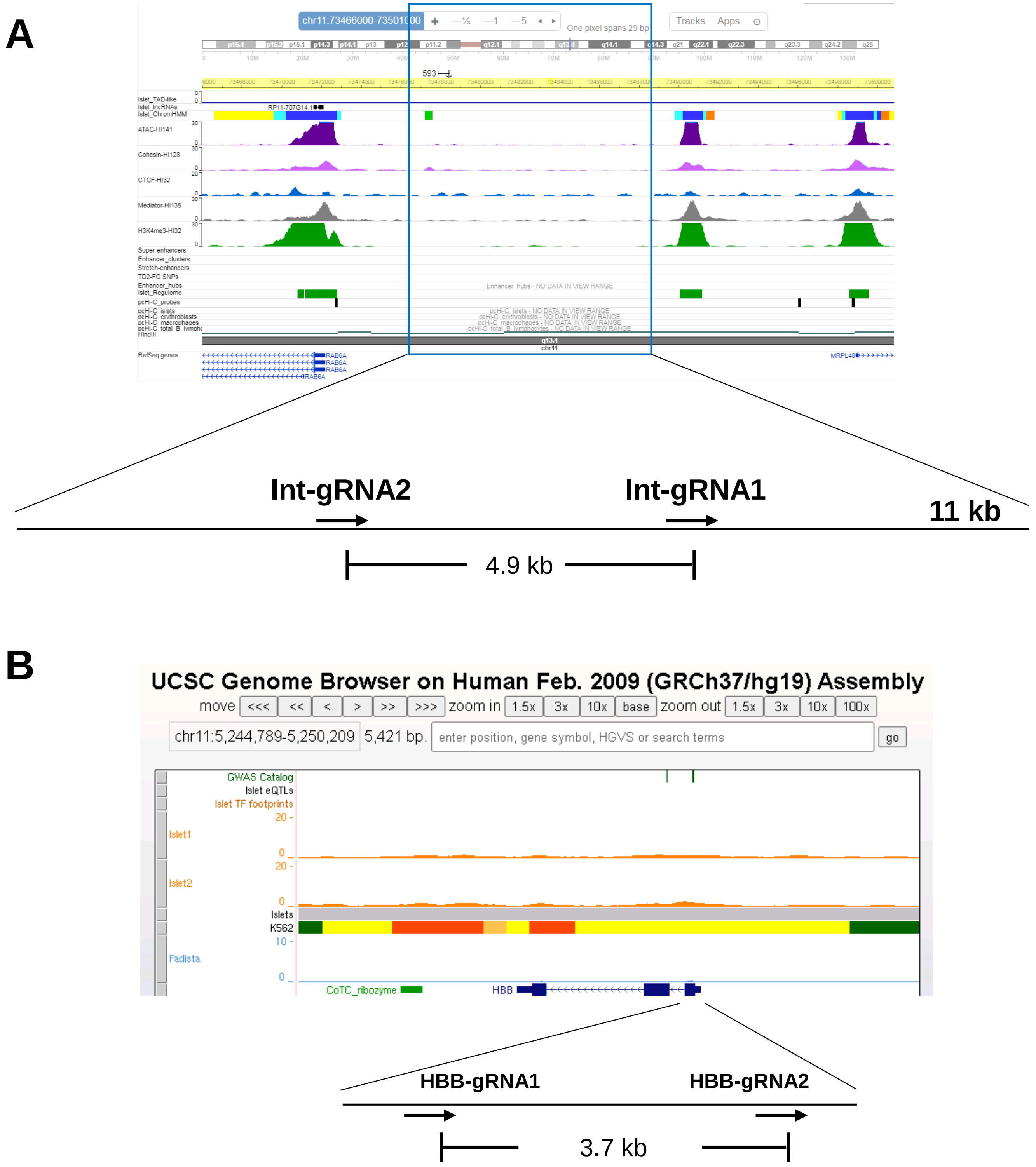

Figure S1. Diagrams of intergenic region and hemoglobin (HBB) gene locus, related to Figure 1D.
(A) Diagram of an intergenic region between *RAB6A* and *MRPL48* genes, 500 Kb downstream of *FCHSD2* gene. The region is epigenetically silent in human islets as demonstrated by the ATAC-seq and epigenomic data. Two gRNAs (gRNA1 and gRNA2) were designed to delete a 4.9 kb genomic DNA fragment.
(B) Diagram of β-globin (*HBB*) gene region. *HBB* gene is not expressed in the human islets. Two gRNAs were designed to delete a 3.7 kb genomic DNA fragment.

Suppl. Fig.1

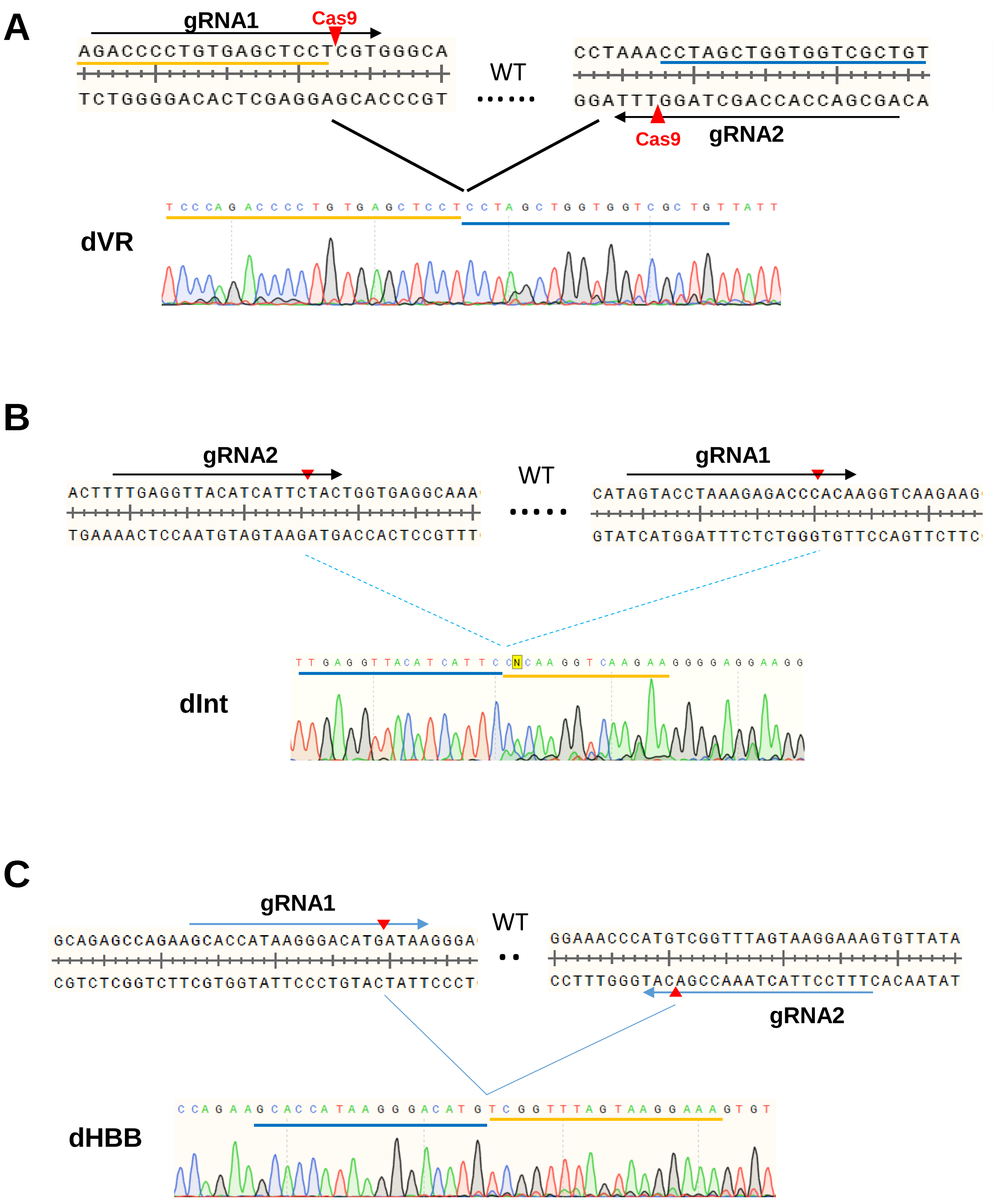
A
gRNA1
Cas9
AGACCCCTGTGAGCTCCTCGTGGGCA
TCTGGGGACACTCGAGGAGCACCCGT
WT
CCTAAACCTAGCTGGTGGTCGCTGT
GGATTTGGATCGACCACCAGCGACA
Cas9
gRNA2
dVR
B
gRNA2
ACTTTTGAGGTTACATCATTCTACTGGTGAGGCAAA
TGAAAACTCCAATGTAGTAAGATGACCACTCCGTTT
WT
gRNA1
CATAGTACCTAAAGAGACCCACAAGGTCAAGAAG
GTATCATGGATTTCTCTGGGTGTTCCAGTTCTTC
dInt
C
gRNA1
GCAGAGCCAGAAGCACCATAAGGGACATGATAAGGGA
CGTCTCGGTCTTCGTGGTATTCCCTGTACTATTCCCT
WT
GGAAACCCATGTCGGTTTAGTAAGGAAAGTGTTATA
CCTTTGGGTACAGCCAAATCATTCCTTTCACAATAT
gRNA2
dHBB

Suppl. Fig.2

Figure S2. Deletion of genomic DNA in EndoC-βH1 cells, related to Figure 1D.

(A) Sanger sequencing of PCR product amplified from wild-type and dVR cell. Orange and blue bars: 5' and 3' end of DNA sequences flanking VR region, respectively.

(B) Sanger sequencing of PCR product amplified from wild-type and dInt cell. Blue and orange bars: 5' and 3' end of DNA sequences flanking intergenic region, respectively.

(C) Sanger sequencing of PCR product amplified from wild-type and dHBB cell. Blue and orange bars: 5' and 3' end of DNA sequences flanking HBB region, respectively.

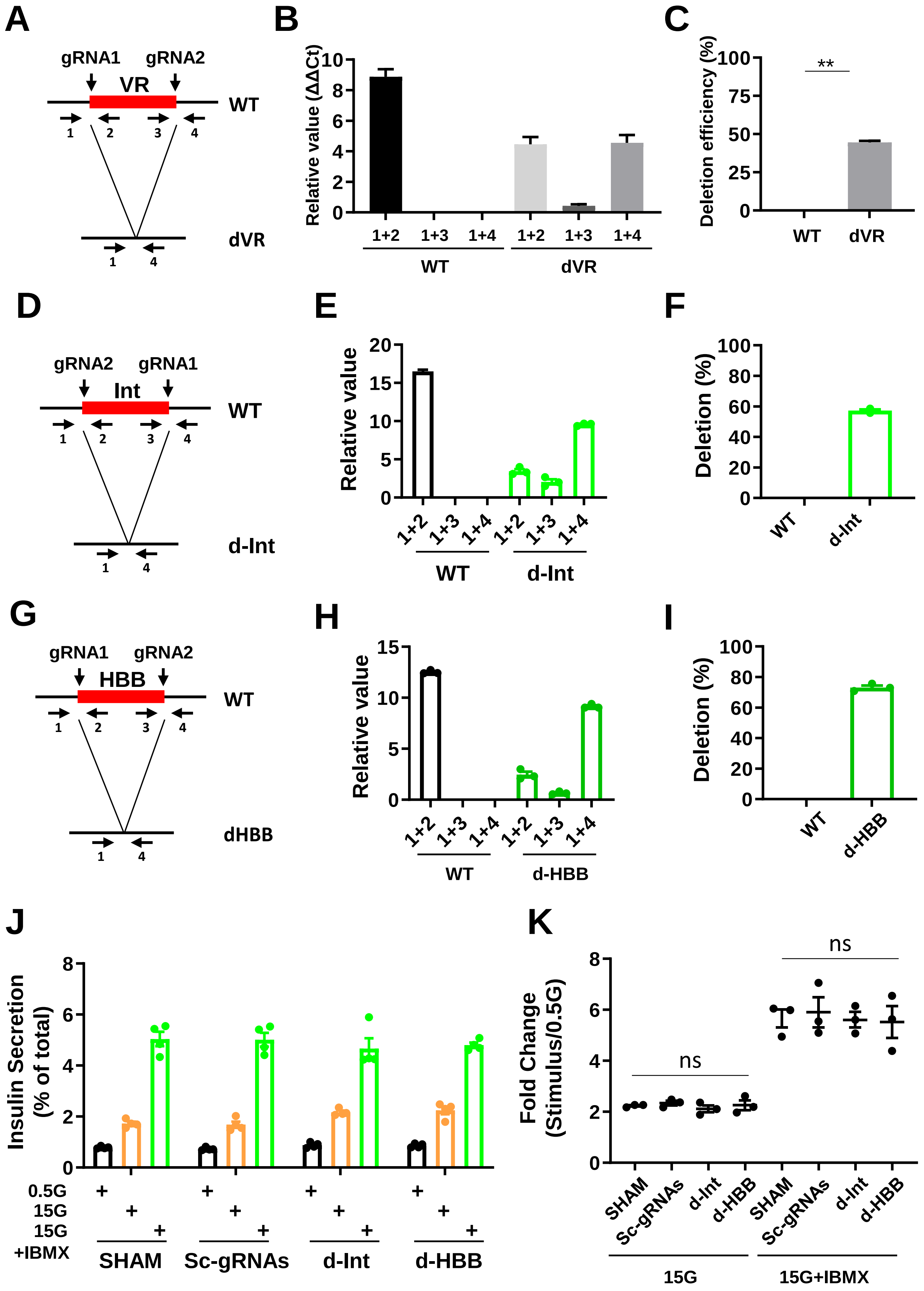

A
gRNA1
gRNA2
VR
WT
dVR
B
Relative value (ΔΔCt)
1+2
1+3
1+4
WT
dVR
C
Deletion efficiency (%)
**
WT
dVR
D
gRNA2
gRNA1
Int
WT
d-Int
E
Relative value
F
Deletion (%)
G
gRNA1
gRNA2
HBB
WT
dHBB
H
Relative value
d-HBB
I
Deletion (%)
J
Insulin Secretion (% of total)
0.5G
15G
15G +IBMX
SHAM
Sc-gRNAs
d-Int
d-HBB
K
Fold Change (Stimulus/0.5G)
ns
ns
15G
15G+IBMX

Suppl. Fig.3

Figure S3. Effect of genome deletion in β cell function, related to Figure 1D-1G.

(A) Diagram of SYBR^{TM} Green qPCR analysis at variant region (VR). Primers 1 and 2 were designed to amplify wildtype genomic DNA; primers 1 and 3 detect inversion of DNA after editing and primers 1 and 4 amplify DNA fragment after deletion. The deletion efficiency rate (%) was determined by: 1. calculating the remaining wildtype allele (primer 1+2) in dVR genomic DNA and compared with wildtype allele in SHAM control and 2. taking away the rate of inversion (primer 1+3) in dVR cells. *CXCL12* gene was served as an internal DNA copy number control.

(B) Representative data of SYBR^{TM} Green qPCR analysis on wildtype and dVR genomic DNAs.

(C) Deletion efficiency. Data are mean ± SEM. *, $P < 0.05$; **, $P < 0.01$; ***, $P < 0.005$. $n = 3$.

(D) Diagram of SYBR^{TM} Green qPCR analysis at intergenic region (Int). Primers 1 and 2 were designed to amplify wildtype genomic DNA; primers 1 and 3 detect inversion of DNA after editing and primers 1 and 4 amplify DNA fragment after deletion. *CXCL12* gene was served as an internal DNA copy number control.

(E) Representative data of SYBR^{TM} Green qPCR analysis on wildtype and dInt genomic DNAs.

(F) Deletion efficiency of the intergenic region. Data are mean ± SEM. *, $P < 0.05$; **, $P < 0.01$; ***, $P < 0.005$. $n = 3$.

(G) Diagram of SYBR^{TM} Green qPCR analysis at *HBB* region. Primers 1 and 2 were designed to amplify wildtype genomic DNA; primers 1 and 3 detect inversion of DNA after editing and primers 1 and 4 amplify DNA fragment after deletion. *CXCL12* gene was served as an internal DNA copy number control.

(H) Representative data of SYBR^{TM} Green qPCR analysis on wildtype and dHBB genomic DNAs.

(I) Deletion efficiency of *HBB* gene region. Data are mean ± SEM. *, $P < 0.05$; **, $P < 0.01$; ***, $P < 0.005$. $n = 3$.

(J-K) GSIS assay of CRISPR-Cas9 control cell lines. (J) Representative data of insulin secretion stimulated by 15 mM glucose or 15 mM glucose plus IBMX. (K) Fold change of secreted insulin. Data are normalized to insulin secretion at basal level (0.5 mM glucose) and drawn from 3 independent experiments ($n = 3$). Data are mean ± SEM. *, $P < 0.05$; **, $P < 0.01$; ***, $P < 0.005$.

.

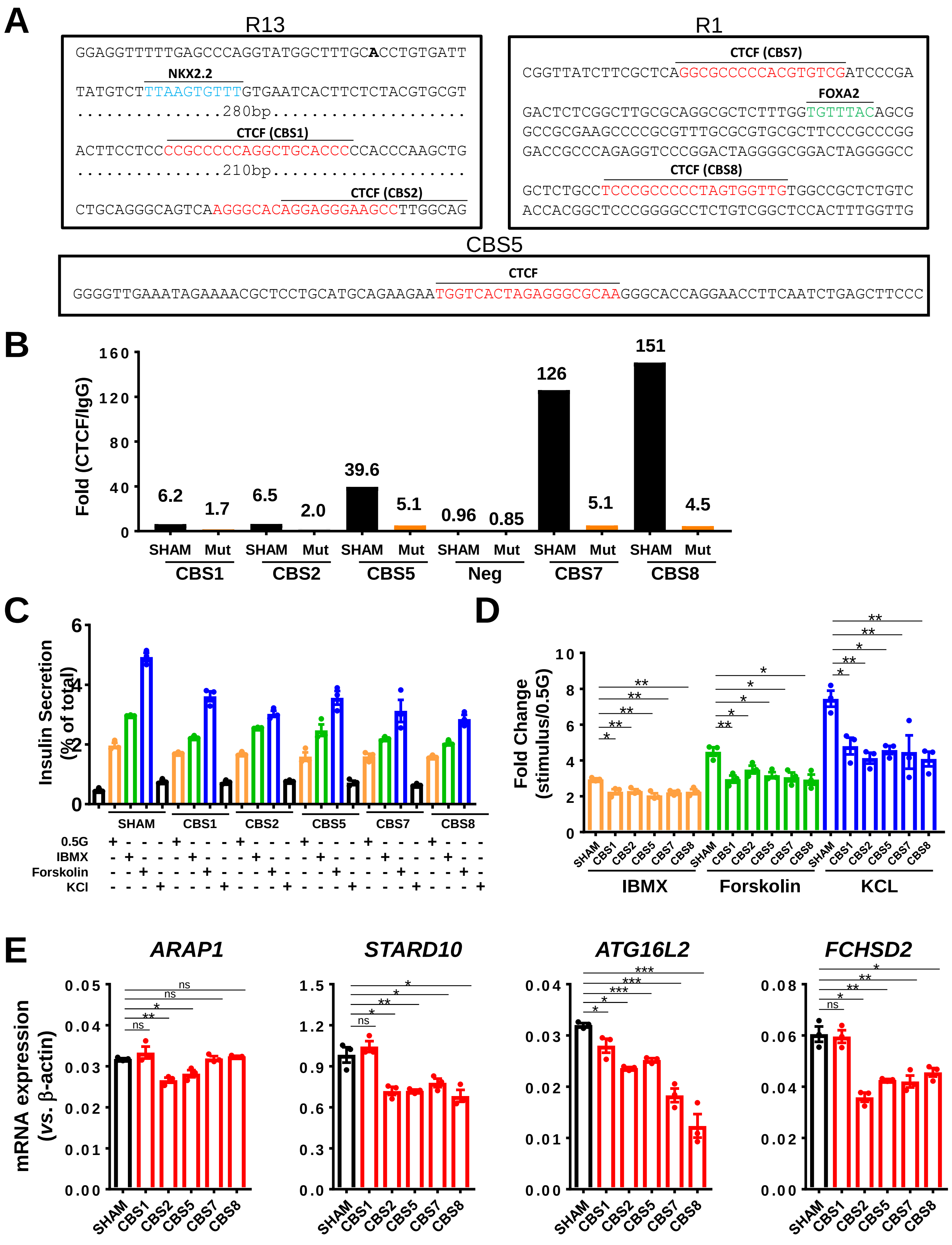
A
R13
GGAGGTTTTTGAGCCCAGGTATGGCTTTGCACCTGTGATT
NKX2.2
TATGTCTTTAAGTGTTTGTGAATCACTTCTCTACGTGCGT
...............280bp...................
CTCF (CBS1)
ACTTCCTCCCCGCCCCCAGGCTGCACCCCCACCCAAGCTG
...............210bp...................
CTCF (CBS2)
CTGCAGGGCAGTCAAGGGCACAGGAGGGAAGCCTTGGCAG
R1
CTCF (CBS7)
CGGTTATCTTCGCTCAGGCGCCCCCACGTGTCGATCCCGA
FOXA2
GACTCTCGGCTTGCGCAGGCGCTCTTTGGTGTTTACAGCG
GCCGCGAAGCCCCGCGTTTGCGCGTGCGCTTCCCGCCCGG
GACCGCCCAGAGGTCCCGGACTAGGGGCGGACTAGGGGCC
CTCF (CBS8)
GCTCTGCCTCCCGCCCCCTAGTGGTTGTGGCCGCTCTGTC
ACCACGGCTCCCGGGGCCTCTGTCGGCTCCACTTTGGTTG
CBS5
CTCF
GGGGTTGAAATAGAAAACGCTCCTGCATGCAGAAGAATGGTCACTAGAGGGCGCAAGGGCACCAGGAACCTTCAATCTGAGCTTCCC
B
Fold (CTCF/IgG)
160
120
80
40
0
6.2
1.7
6.5
2.0
39.6
5.1
0.96
0.85
126
5.1
151
4.5
SHAM Mut SHAM Mut SHAM Mut SHAM Mut SHAM Mut SHAM Mut
CBS1 CBS2 CBS5 Neg CBS7 CBS8
C
Insulin Secretion
(% of total)
6
4
2
0
SHAM CBS1 CBS2 CBS5 CBS7 CBS8
0.5G + - - - + - - - + - - - + - - - + - - - + - - -
IBMX - + - - - + - - - + - - - + - - - + - - - + - -
Forskolin - - + - - - + - - - + - - - + - - - + - - - + -
KCl - - - + - - - + - - - + - - - + - - - + - - - +
D
Fold Change
(stimulus/0.5G)
10
8
6
4
2
0
SHAM CBS1 CBS2 CBS5 CBS7 CBS8 SHAM CBS1 CBS2 CBS5 CBS7 CBS8 SHAM CBS1 CBS2 CBS5 CBS7 CBS8
IBMX
Forskolin
KCL
E
mRNA expression
(vs. β-actin)
ARAP1
0.05
0.04
0.03
0.02
0.01
0.00
STARD10
1.5
1.2
0.9
0.6
0.3
0.0
ATG16L2
0.04
0.03
0.02
0.01
0.00
FCHSD2
0.08
0.06
0.04
0.02
0.00
SHAM CBS1 CBS2 CBS5 CBS7 CBS8
ns
*
**
***

Suppl. Fig.4

Figure S4. CTCF-binding site (CBS) at *STARD10* locus, related to Figure 3.

(A) DNA sequencing diagrams of ChIP-qPCR identified CBSs at R13 and R1 regions. CBS1 and 2 are within R13, CBS5 is 2.7 kb downstream of R13, and CBS7 and 8 are within R1 region.

(B) Comparison of CTCF binding affinity before and after CRISPR-Cas9 editing at CBSs. CTCF binding affinity was normalized to IgG control in wild-type and CBS mutate cells. The numbers above each bar represent the fold change before and after genome editing. The data are pulled from two independent experiments.

(C) Representative data of Insulin secretion stimulated by multiple stimuli. Cells were treated with 0.5 mM glucose for an hour and then stimulated with either IBMX (0.5 μM) or Forskolin (20 nM) or KCl (20 μM). Data are mean ± SEM. *, $P < 0.05$; **, $P < 0.01$; ***, $P < 0.005$.

(D) Fold change of secreted insulin. Data are normalized to insulin secretion at basal level (0.5 mM glucose) and drawn from 3 independent experiments ($n = 3$). *, $P < 0.05$; **, $P < 0.01$; ***, $P < 0.005$.

(E) Representative data of Taqman™ qRT-PCR analysis in CBS mutate cells. G. *ARAP1*; H. *STARD10*; I. *ATG16L2* and J. *FCHSD2*. *, $P < 0.05$; **, $P < 0.01$; ***, $P < 0.005$. $n = 3$.

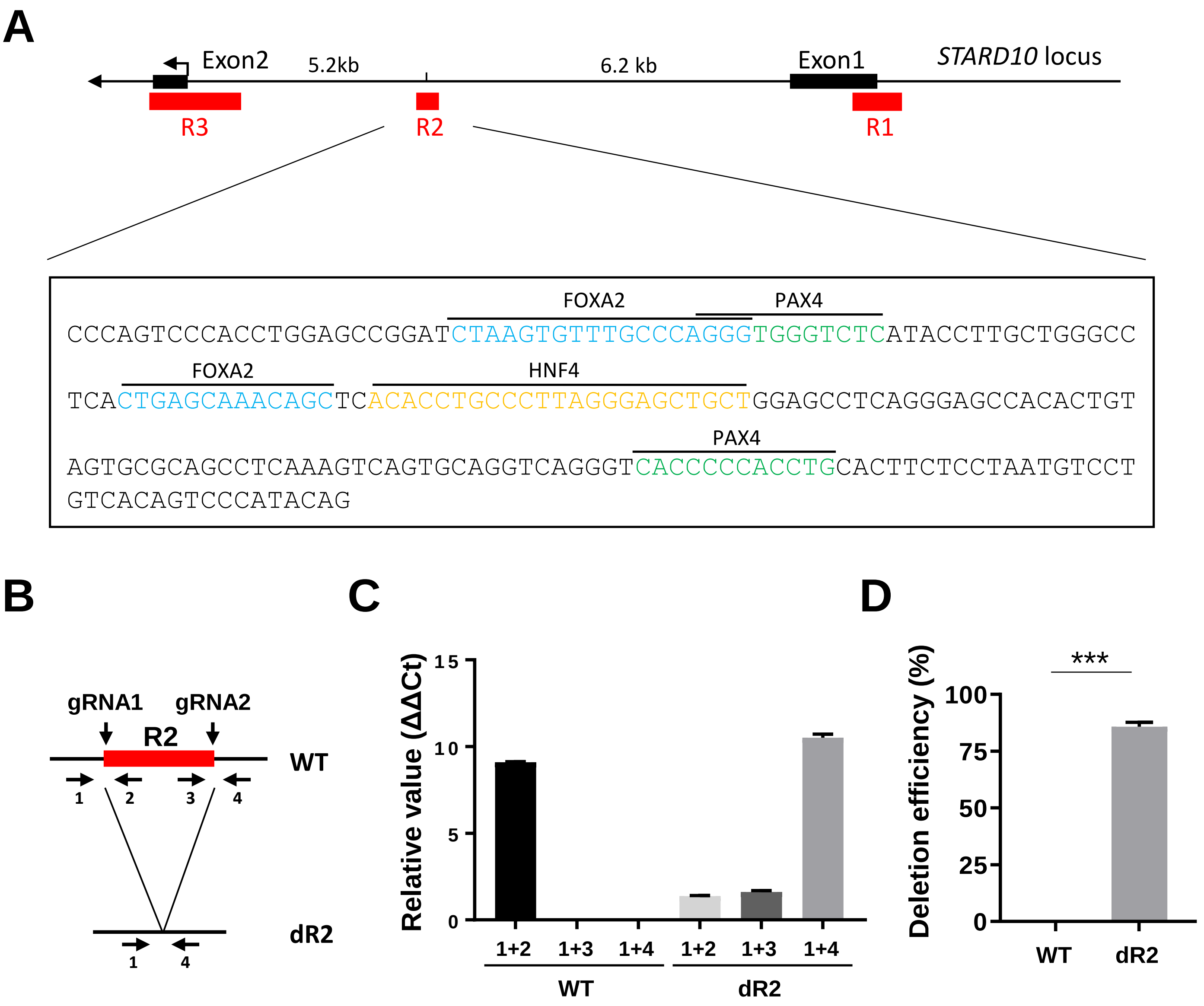

Figure S5. Deletion of R2 enhancer in EndoC-βH1 cells, related to Figure 4.
(A) Diagram of genomic DNA surrounding R2 region. R2 enhancer is located between two promoters of *STARD10* gene (R3 and R1) with multiple binding sites for islet-associated transcription factors.
(B) Diagram of primers designed for SYBR^{TM} Green PCR analysis at R2 region. Primers 1 and 2 were designed to amplify wildtype genomic DNA; primers 1 and 3 were to detect DNA inversion after genome editing and primers 1 and 4 were to amplify DNA fragment after deletion. CXCL12 gene was served as an internal DNA copy number control.
(C) Representative data of SYBR^{TM} Green qPCR analysis in SHAM and dR2 cells.
(D) Deletion efficiency. Data are mean ± SEM. *, $P < 0.05$; **, $P < 0.01$; ***, $P < 0.005$. $n = 2$.

Suppl. Fig.5

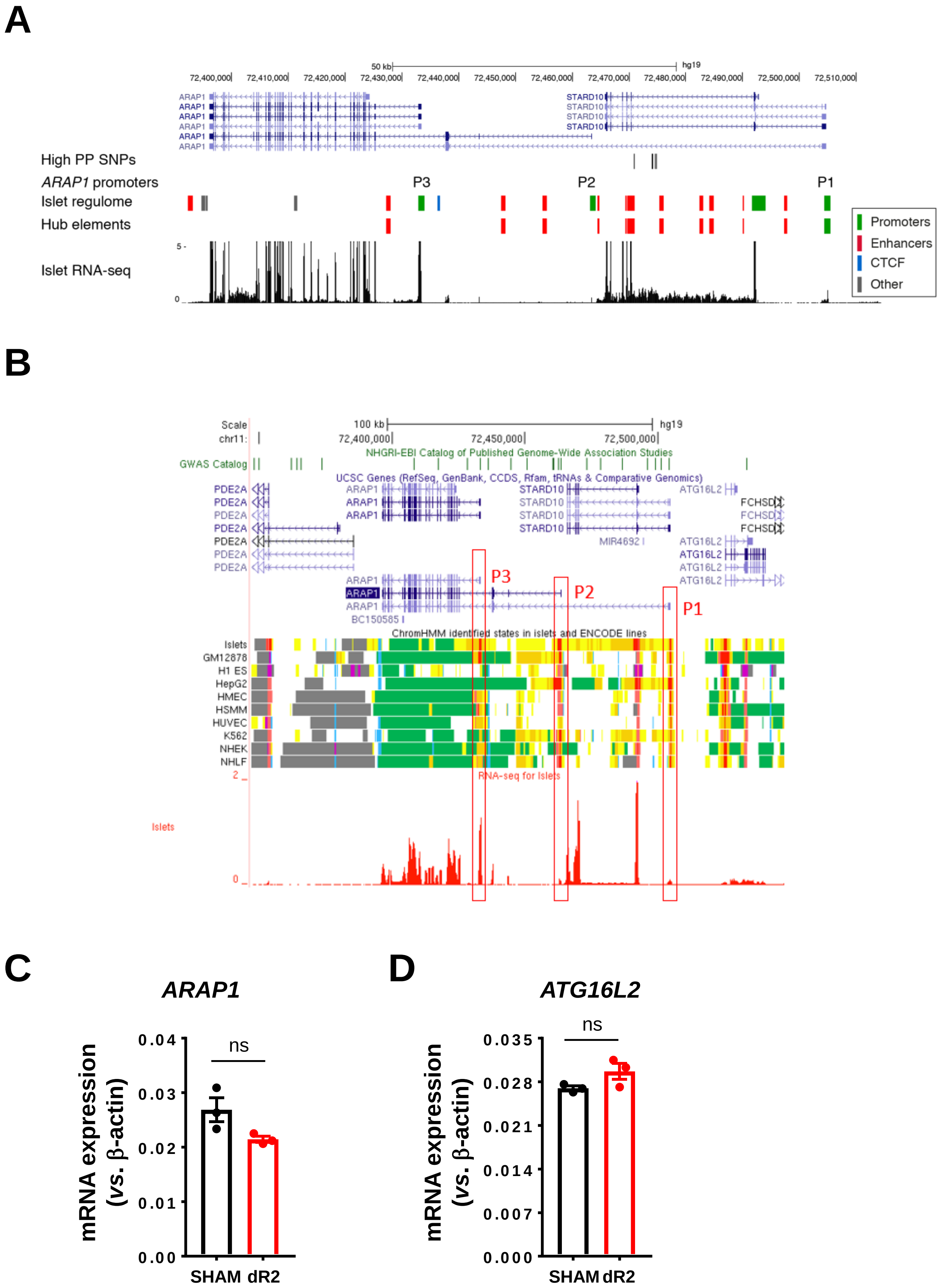
A
50 kb
hg19
72,400,000
72,410,000
72,420,000
72,430,000
72,440,000
72,450,000
72,460,000
72,470,000
72,480,000
72,490,000
72,500,000
72,510,000
ARAP1
STARD10
High PP SNPs
ARAP1 promoters
P3
P2
P1
Islet regulome
Hub elements
Islet RNA-seq
5 -
0
Promoters
Enhancers
CTCF
Other
B
Scale
chr11:
100 kb
hg19
72,400,000
72,450,000
72,500,000
NHGRI-EBI Catalog of Published Genome-Wide Association Studies
GWAS Catalog
UCSC Genes (RefSeq, GenBank, CCDS, Rfam, tRNAs & Comparative Genomics)
PDE2A
ARAP1
STARD10
ATG16L2
FCHSD2
MIR4692
BC150585
P3
P2
P1
ChromHMM identified states in islets and ENCODE lines
Islets
GM12878
H1 ES
HepG2
HMEC
HSMM
HUVEC
K562
NHEK
NHLF
RNA-seq for Islets
2
Islets
0
C
ARAP1
mRNA expression
(vs. β-actin)
0.04
0.03
0.02
0.01
0.00
ns
SHAM
dR2
D
ATG16L2
mRNA expression
(vs. β-actin)
0.035
0.028
0.021
0.014
0.007
0.000
ns
SHAM
dR2

Suppl. Fig.6

Figure S6. Expression of local genes at *ARAP1*/*STARD10* locus, related to Figure 5.

(A) Genome browser view of the *ARAP1/STARD10* locus displaying the three putative promotors for *ARAP1* (P1-3). Human islet RNA-seq track shows that exons belonging to *ARAP1* isoforms expressed from P1 and P2 are very lowly expressed in comparison to the isoform driven by the P3 promoter. We have modified the scale range to highlight this better. Please note that only P1 belongs to the islet enhancer hub (hub elements track). High PP SNPs track shows all T2D credible set SNPs with PP > 0.05 (Carrat et al., 2017).

(B) Genome browser view of the *ARPA1/STARD10* locus with RNA-seq data. Red box: correlation between promoter region and RNA-seq peak (Parker et al., 2013).

(C) Representative data of *ARAP1* gene expression in dR2 cells. *, $P < 0.05$; **, $P < 0.01$; ***, $P < 0.005$. $n = 3$.

(D) Representative data of *ATG16L2* gene expression in dR2 cells. *, $P < 0.05$; **, $P < 0.01$; ***, $P < 0.005$. $n = 3$.

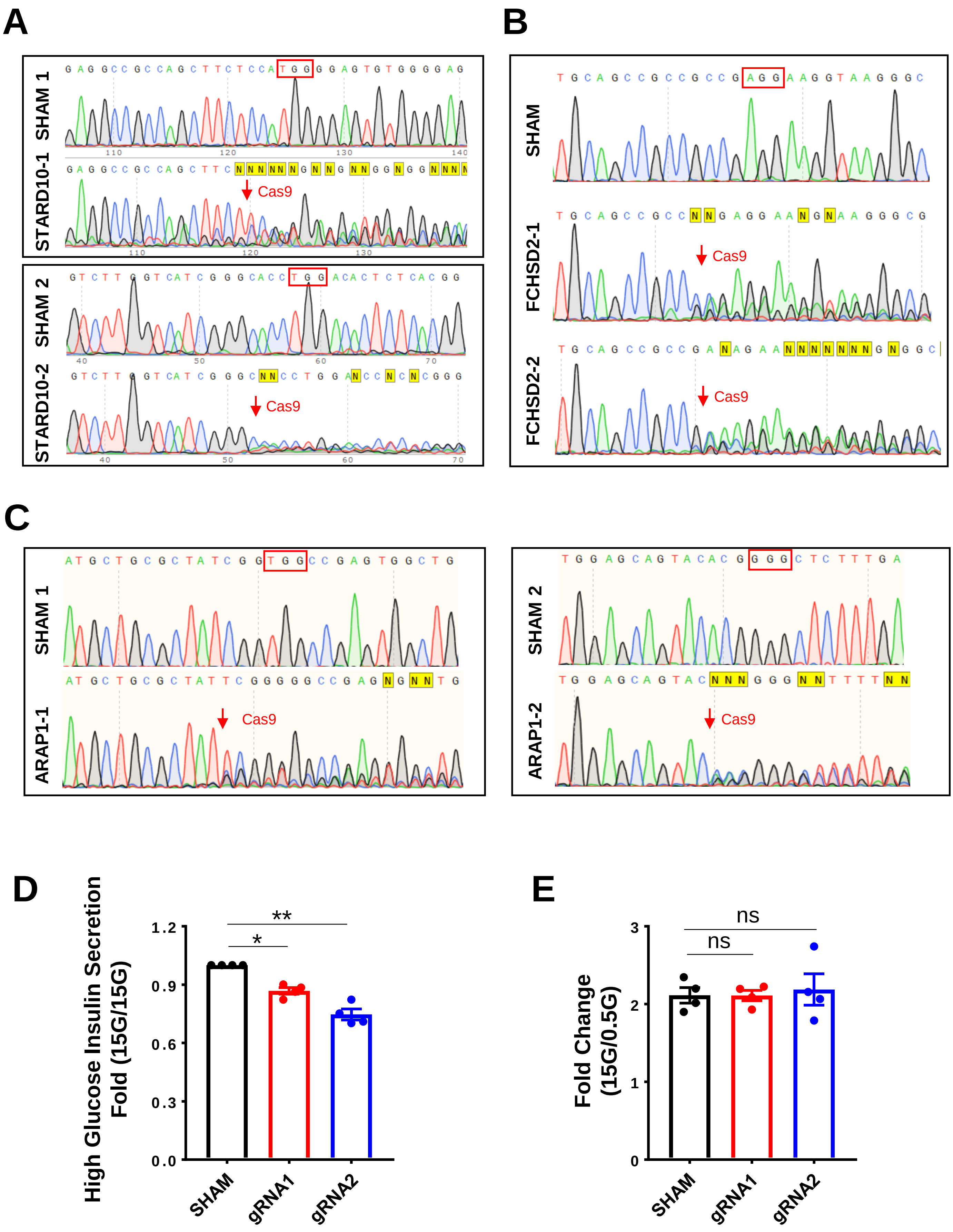
A
SHAM 1
STARD10-1
Cas9
SHAM 2
STARD10-2
Cas9
B
SHAM
FCHSD2-1
Cas9
FCHSD2-2
Cas9
C
SHAM 1
ARAP1-1
Cas9
SHAM 2
ARAP1-2
Cas9
D
High Glucose Insulin Secretion
Fold (15G/15G)
1.2
0.9
0.6
0.3
0.0
**
*
SHAM
gRNA1
gRNA2
E
Fold Change
(15G/0.5G)
3
2
1
0
ns
ns
SHAM
gRNA1
gRNA2

Suppl. Fig.7

Figure S7. Knockout effects of *STARD10*, *FCHSD2* or *ARAP1* in EndoC-βH1 cells, related to Figure 6.

(A) Saner sequencing of PCR products amplified from wild-type and *STARD10*-KO cells. Red box: PAM sequence; red arrow: Cas9 cutting site.

(B) Sanger sequencings of PCR products amplified from wild-type and *FCHSD2*-KO cells. Red box: PAM sequence; red arrow: Cas9 cutting site.

(C) Sanger sequencings of PCR products amplified from wild-type and *ARAP1*-KO cells. Red box: PAM sequence; red arrow: Cas9 cutting site.

(D-E) GSIS assay of *FCHSD2*-KO cells. (D) Fold change at high glucose level (15 mM glucose vs. 15 mM glucose) (E) Fold change of secreted insulin at 15 mM glucose vs. 0.5 mM glucose. Data are drawn from 4 independent experiments (n = 4). Data are mean ± SEM. *, $P < 0.05$; **, $P < 0.01$; ***, $P < 0.005$.

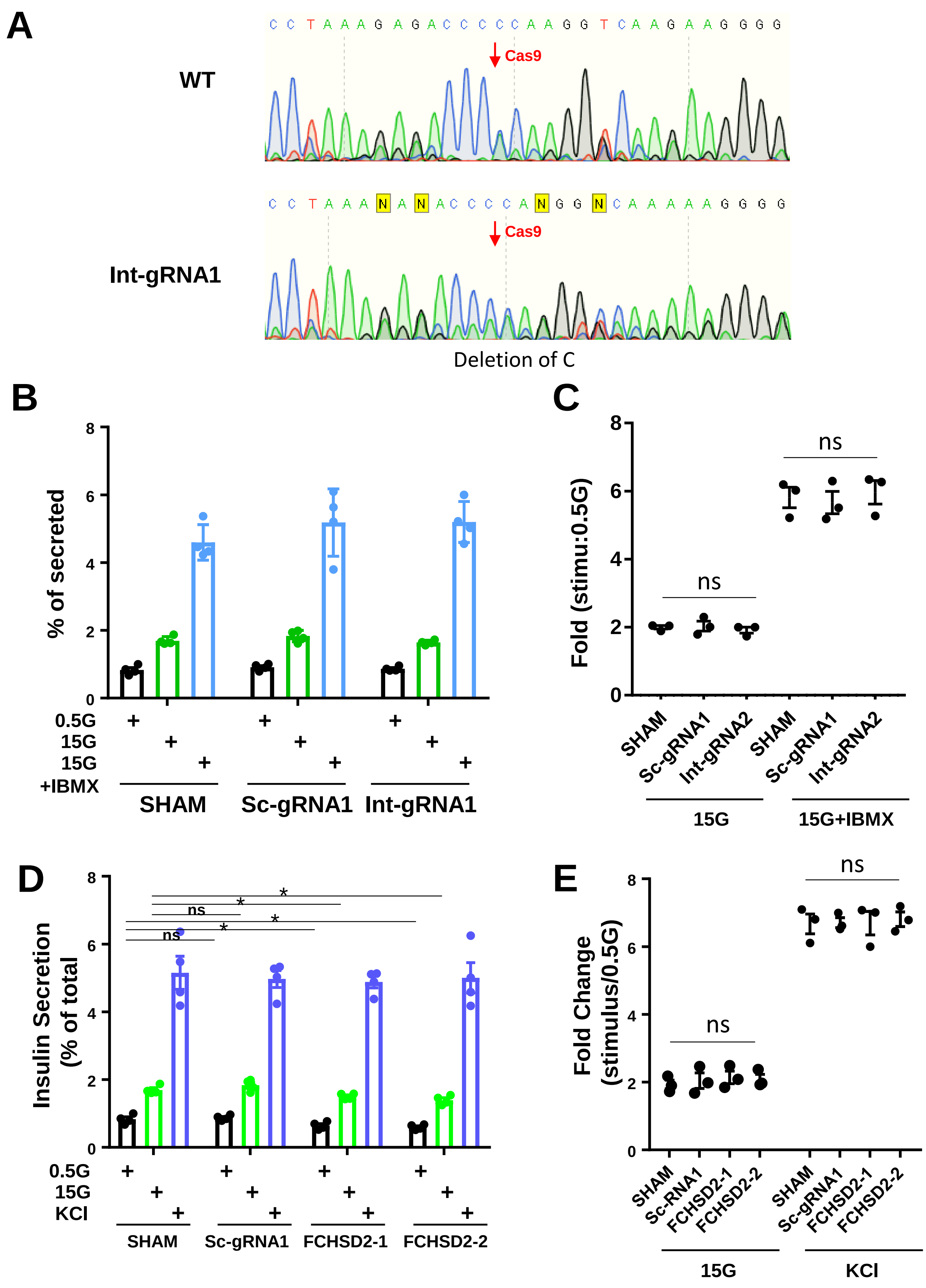
A
WT
C C T A A A G A G A C C C C C A A G G T C A A G A A G G G G
Cas9
Int-gRNA1
C C T A A A N A N A C C C C A N G G N C A A A A A G G G G
Cas9
Deletion of C
B
% of secreted
0.5G
15G
15G
+IBMX
SHAM
Sc-gRNA1
Int-gRNA1
C
Fold (stimu:0.5G)
ns
ns
SHAM
Sc-gRNA1
Int-gRNA2
SHAM
Sc-gRNA1
Int-gRNA2
15G
15G+IBMX
D
Insulin Secretion
(% of total
ns
ns
0.5G
15G
KCl
SHAM
Sc-gRNA1
FCHSD2-1
FCHSD2-2
E
Fold Change
(stimulus/0.5G)
ns
ns
SHAM
Sc-RNA1
FCHSD2-1
FCHSD2-2
SHAM
Sc-gRNA1
FCHSD2-1
FCHSD2-2
15G
KCl

Suppl. Fig.8

Figure S8. Effects of DNA mutation in β cell function, related to Figure 6.

(A) Sanger sequencing of PCR products amplified from wild-type and intergenic region gRNA1-cutting (int-gRNA1) cells. Red box: PAM sequence; red arrow: Cas9 cutting site.

(B-C) GSIS assay of CRISPR-Cas9 control cells. (B) Representative data of insulin secretion. (C) Fold change of secreted insulin. Data are normalized to insulin secretion at basal level (0.5 mM glucose) and drawn from 3 independent experiments (n = 3). Data are mean ± SEM. *, $P < 0.05$; **, $P < 0.01$; ***, $P < 0.005$.

(D-E) GSIS assay of *FCHSD2*-KO cells stimulated by KCl. (D) Representative data of insulin secretion. (E) Fold change of secreted insulin. Data are normalized to insulin secretion at basal level (0.5 mM glucose) and drawn from 3 independent experiments (n = 3). Data are mean ± SEM. *, $P < 0.05$; **, $P < 0.01$; ***, $P < 0.005$.

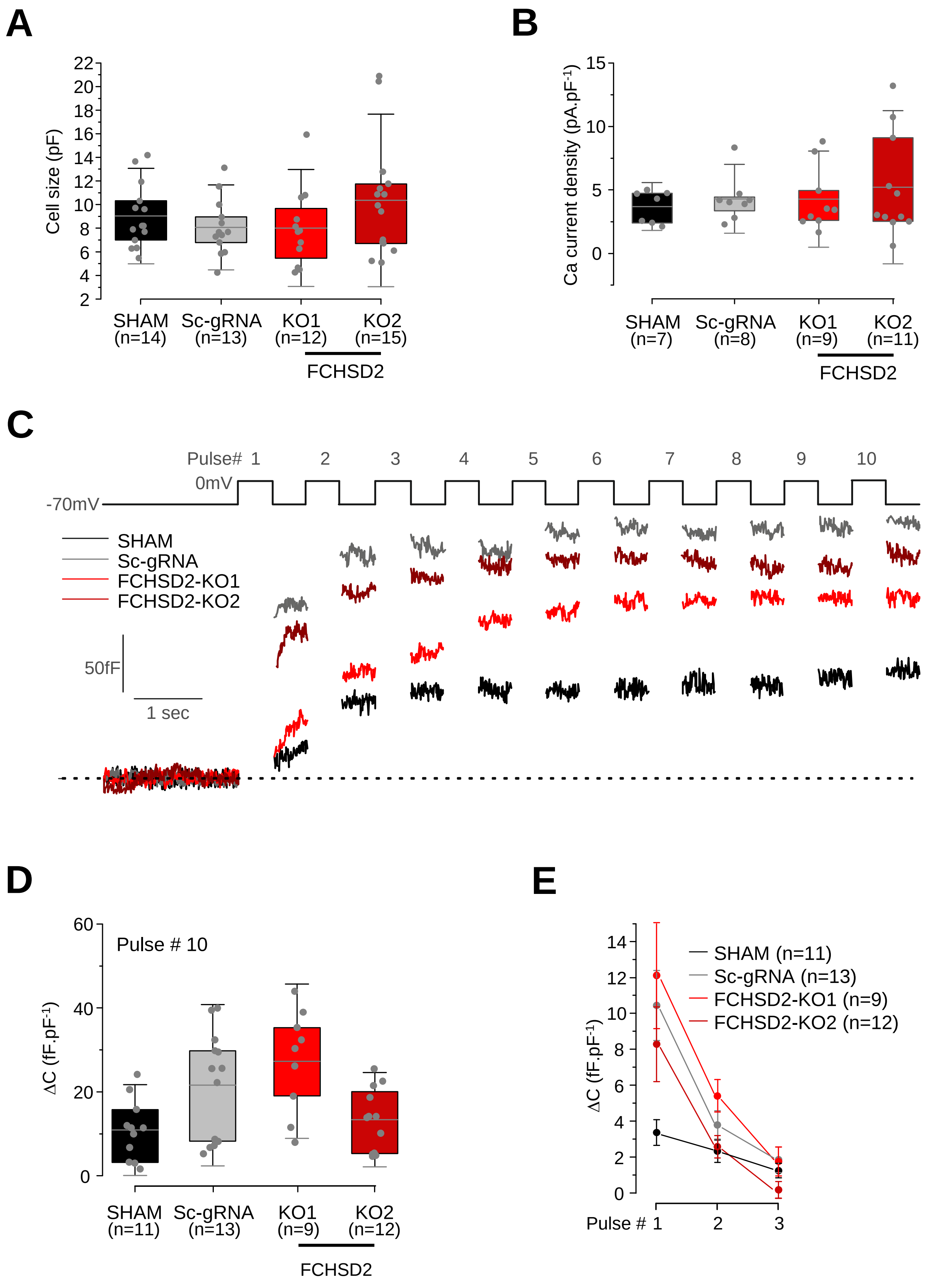

A
22
20
18
16
14
12
10
8
6
4
2
Cell size (pF)
SHAM
(n=14)
Sc-gRNA
(n=13)
KO1
(n=12)
KO2
(n=15)
FCHSD2
B
15
10
5
0
Ca current density (pA.pF-1)
SHAM
(n=7)
Sc-gRNA
(n=8)
KO1
(n=9)
KO2
(n=11)
FCHSD2
C
Pulse# 1
2
3
4
5
6
7
8
9
10
0mV
-70mV
SHAM
Sc-gRNA
FCHSD2-KO1
FCHSD2-KO2
50fF
1 sec
D
60
40
20
0
Pulse # 10
ΔC (fF.pF-1)
SHAM
(n=11)
Sc-gRNA
(n=13)
KO1
(n=9)
KO2
(n=12)
FCHSD2
E
14
12
10
8
6
4
2
0
ΔC (fF.pF-1)
SHAM (n=11)
Sc-gRNA (n=13)
FCHSD2-KO1 (n=9)
FCHSD2-KO2 (n=12)
Pulse # 1
2
3

Suppl. Fig.9

Figure S9. Impact of *FCHSD2*-KO on exocytosis properties, related to Figure 6D-6F.

(A) Comparison of the single cell sizes measured in each cell line (pF).

(B) Calcium current density measured in response to a 100ms depolarisation from -70mV to 0mV. The current density was determined by normalising the amplitude of calcium current (pA) by the cell size (pF).

(C) Representative traces in each cell line of exocytosis elicited by 10 stimulations from -70mV to 0mV.

(D) Amplitude of exocytosis normalised by cell size ($fF.pF^{-1}$) measured at the 10th pulse (Pulse#10).

(E) Increment in exocytosis triggered at each of the three firsts pulses. All box plots (A, B, and C) display the interquartile range (IQR) with means, whiskers indicating 1.5x the SD, and all data points. For clarity, line plots (E) display means ± sem. Ns represents the number of cells measured per cell line and are indicated in each panels. Data were compared using Welch's ANOVA, which does not make the assumption of equal variance in each population, followed by Games-Howell post-hoc test and p-values adjusted by Benjamini & Hochberg procedure.

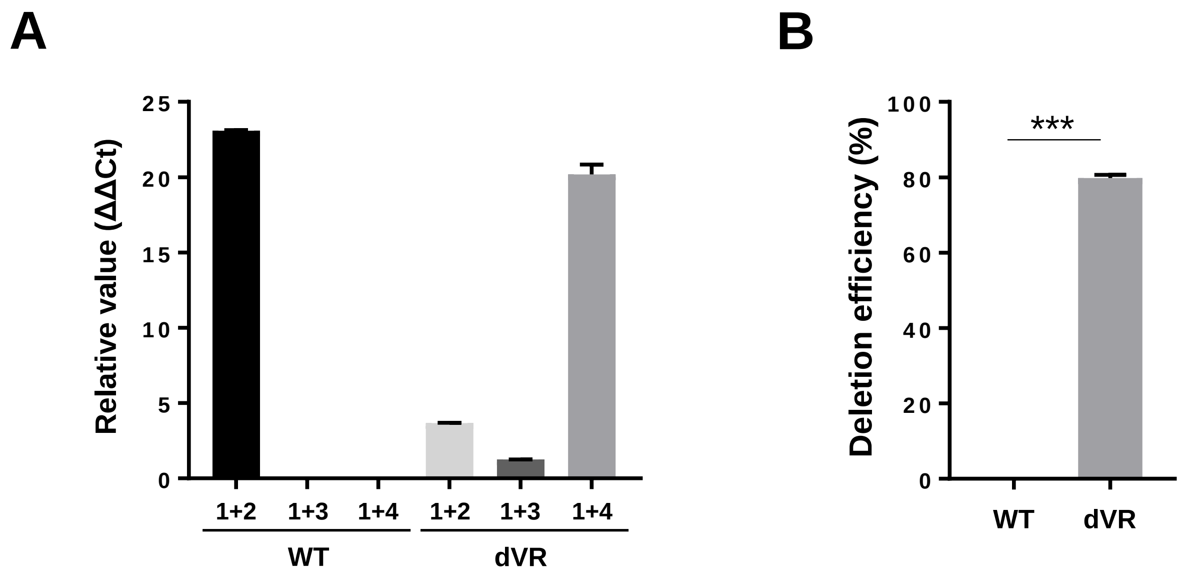

Figure S10. Deletion efficiency of variant region (VR) by CRIPSR-Cas9 genome editing, related to Figure 7.

(A) Representative data of SYBR^{TM} Green qPCR analysis on wildtype and dVR genomic DNAs.

(B) Deletion efficiency. Data are mean ± SEM. *, *P* < 0.05; **, *P* < 0.01; ***, *P* < 0.005. *n* = 1.

Table S1 Transcription factor binding affinity at genetic variants
Related to Fig 1B

**variant**	**gene**	**Score**	
		Risk allele	Protective allele
rs79430446	GATA3	4.6395	6.22367
	GATA5	4.36019	8.01532
	GATA6	6.53008	8.17938
	MAFK	6.15646	-
	NRL	5.70744	-
	MAFF	7.22087	-
	TBP	6.96517	-
	GSC	-	8.9786
	GSC2	-	8.55269
	BARX1	-	6.55349
	BSX	-	5.48796
	EVX1	-	6.53802
	FOXP3	-	5.80405
	OTX2	-	9.73373
	OTX1	-	8.01065
	HOXB2	-	5.44904
	HOXB3	-	4.68114
	MEF2C	-	8.10057
	MEF2A	-	7.64429
	RHOXF1	-	6.92681
	VENTX	-	6.05976
	POU3F4	5.81505	10.7694
	POU5F1B	5.84768	11.6522
	POU5F1	-	8.37144
	POU2F1	-	9.09201
	POU3F1	-	7.69322
	POU3F2	-	7.49311
	POU3F3	-	7.84807
	RFX5	7.526	-
rs140735484	SP1	7.6665	10.0444
	HIC2	-	5.55379
	SP3	-	5.90913
	CDX2	5.8768	-
rs7103836	HLF	7.50884	-
	GATA6	5.78747	-
	SOX10	10.8334	5.84552
	THAP1	6.55426	-
	SOX15	6.50967	-
	RUNX3	5.15967	-
	SOX13	5.39057	-
	FOXP3	5.92425	-
	BARHL2	7.58065	-
rs613937	GSC2	6.72964	-

	OTX1	7.62999	-
	NKX3-2	5.9364	-
	EBF1	4.59291	9.45164
	RFX5	-	5.60305

Table S2 eQTL analysis of *FCHSD2* and *STARD10* gene expression in human islet samples. Related to Fig. 5D

Gene	Variant ID	Position (hg19)	Ref allele	Alt allele	MAF	Risk allele	Nominal P value	rs140130268 R2 (EUR)
FCHSD2	rs11603334	Chr11:72432985	G	A	0.144	G	0.0127082	0.8934
FCHSD2	rs1552224	Chr11:72433098	A	C	0.144	A	0.0127082	0.8934
FCHSD2	rs75896506	Chr11:72429829	G	A	0.144	G	0.0127082	0.8934
STARD10	rs11603334	Chr11:72432985	G	A	0.144	G	2.98×10^{-4}	0.8934
STARD10	rs1552224	Chr11:72433098	A	C	0.144	A	2.98×10^{-4}	0.8934
STARD10	rs75896506	Chr11:72429829	G	A	0.144	G	2.98×10^{-4}	0.8934

Table S3. Primers and probe sequences, related to gRNAs in STAR Methods.

Name	Sequence (R, risk, P, protective allele)
**EMSA assay**	
rs140130268	TATTTGTGGTTTGTTTGTTTGTTTTTCCT (R)
	TATTTGTGGTTTGTTTGTTTTTCCT (P)
rs79430446	GTATAAGATGAGCATGGAAC (R)
	GTATAAGATTAGCATGGAAC (P)
rs140735484	AAATCCCCCAGTCCCAGGCA (R)
	AAATCCCCC-GTCCCAGGCA (P)
rs7103836	TCTGCCACAGAGACATAACA (R)
	TCTGCCACACAGACATAACA (P)
rs613937	CAGCCTCCTAAGCGGCCACA (R)
	CAGCCTCCTGAGCGGCCACA (P)
**genomic DNA PCR amplification**	
VRdel_F1	CCATCTCCCCCGACTCAGCCCAG
VRdel_R1	GGGAGATCCGATTTTGAGTCCCTGC
VRdel_F2	CGACTCAGCCCAGTCTCCTCC
CBS1&2_F	GCAGCCGTGGCCAACACACACACTTCC
CBS1&2_R1	CTCGTGGTGGGGTGCTTGCTGAGG
CBS1&2_R2	GGAGCCCAGAGATGCTGAGAAACTTGC
CBS5_F	CCACTTCCAACCCCAGAGAC
CBS5_R1	CATACTCAGGGGGCCTTGTG
CBS5_R2	CCTTGTGGGGAGGGTCTGGGAG
CBS7&8_F	CACCCCTGGATCTCATTTGATCCTCC
CBS7&8_R1	GCAGTCCTTGAATCCTGATCCTTCCCTGG
CBS7&8_R2	GGTCAGAAGGACGATGCCGAGCGC
R2_F1	GTCAGAAGGCTGAGGCAAGAGGATGG
R2_F2	CAGTGAGCTGAGATGTTGCCACAGC
R2_R	CCACTTTGGTGCCATGTGTGGCCTGG
STARD10_CRISPR_F	CACCCCAGCCCTGCTATAGGTCAGG
STARD10_CRISPR_R	GCCCCCAGCGCACTGATTCCCGTCC
STARD10_CRISPR_R2	GCACTGATTCCCGTCCCCAAACCG
FCHSD2_CRISPR_F1	CTCCCTCGTCTCCTCACACTCG
FCHSD2_CRISPR_F2	TGCCGCCCGCTGGCCTGCTCC
FCHSD2_CRISPR_R	CCCAAGACGAGGGCGGTCACG
ARAP1_CRISPR_F1	CTATGGCTTGAGAGAGCCATGCAGG
ARAP1_CRISPR_F2	GCTGCTGGTGAGGGGACCAATCC
ARAP1_CRISPR_R	CTTCATGGGCACAGGCCGTGG
HBB-F	GACTGGGAGAGAGGACAAGGACC
HBB-R1	GAGTGAGATTTTTTCACAAGTACCTGATGAGG
HBB-R2	GTACCTGATGAGGGTTGAGACAGG
Int-del-F1	CCGGATTTCACCACATTGGCCAGG
Int-del-F2	CTGACGTCAAGTGATGCACCTGC
Int-del-R	CACTCCAATGCCCACCTGTTGTGG
**SYBR Green qPCR assay**	
dVR_1	CCTTCTGGGCTCCCACACAATGC
dVR_2	CTGCCCCAAATGTTCAACACGC

dVR_3	TGTTCAACACGCACTCATTCTTCACC
dVR_4	GTTGCTGAATCCCCCAAGCTTCAG
dR2_1	GCTGGCTCGGCCTTGAGAGG
dR2_2	GGCAAACACTTAGATCCGGCTCC
dR2_3	TTCTCCTAATGTCCTGTCACAGTCCC
dR2_4	ACTTTGGTGCCATGTGTGGCCTGG
qHBB_1	AACACTATGCTAATAACTGCAGAGCCAG
qHBB_2	GATTAGCATTCAGGAAGAGATCAGAGG
qHBB_3	GGCCCTGTCAGTCATCCTGAATCC
qHBB_4	GCTGAAAGGAAGAAGTAGGAGAAACATGC
qInt_1	AGCATGAGTCACTGTGCCCAGC
qInt_2	GCCTGGGTGACAGAGCAAGACTCC
qInt_3	GAAAGAAAGAAATGTGGTTCTAAGGAAAGG
qInt_4	CTAAAACTAAACTTACCACCTTCCTCCCC

4C assay at VR region

4C_F1	GGTATAGCCTGCTACTCAAAAGGTCCCTGG
4C_R1	TACCTCCTGCACTGAGATTCTCCATGAAGC
4C_F2_XhoI	AATTTCTCGAGCTTCTTGTGCCAGGAAGTAAGC
4C_R2-NotI	ATGTGCGGCCGCGGACATCTCCCCATTTCCAAGG

Taqman™ primer/probe for 3C assay

Probe at VR region	AGATGTCCTAAAGTGCTCATTGGGGGCATA
Constant primer	CAGTGAAAGGAGGAGCCCACC
NcoI Fragment	
-13	ACTTCTGTGAGCTCCCTGAGG
-12	ACCTTGTCCGCTCTCAGTCC
-11	CTCTCAGAGCCTGTCTGAACATAGC
6	CCCTTGGGGCTCTGTAGAGG
7	CCAAACCCACACCTGGAACAGG
8	CCAGCTCCACCGCTCCAAGG
9	ACAGGCGTGAGCCATCATGC
Probe at R13 region	CCAGGCCTGGCCCTGTGCTGGCTCCTGAGG
Constant primer	ACTTCTGTGAGCTCCCTGAGG
NcoI Fragment	
12	CCCAACCTTTTTGGCACCAGG
13	CCGTGATGTCATCACCCTCC
14	CCTCCTGCACTGAGATTCTCC
15	GCAGCTTATCTCAGATTGAGCCC
17	CCTGGGTCCCTAGGACTTTGG
18	CTGGCAGAGGTGGTTTGAGC
19	CGGAGCCTCCGCGGAGGACC
20	CCAGAACACCAGGGACTCACG
21	ACTCCCCAGCCAGGTGAAGC
23	CAAGCGTGAGCCACTGCACC

Taqman™ primer for gene expression (Thermo Fisher Scientific)

CXCL12	Cat# 4400291; Assay ID: Hs02117611_cn
STARD10	Cat# 4331182; Assay ID: Hs00246405_ml
ARAP1	Cat# 4331182; Assay ID: Hs00362929_ml
FCHSD2	Cat# 4331182; Assay ID: HS00207952_ml

ATG16L2	Cat# 4331182; Assay ID: Hs01057324_ml
ACTB	Cat# 4331182; assay ID: Hs01060665_gl

SYBR Green qPCR assay at CTCF binding site (CBS)

CBS1_F	AAAGTCACCCAGAATCCCCC
CBS1_R	CAGGGCTTGTCAGTCAGGAC
CBS2_F	ATCAGCCTCCAGGAAGACCA
CBS2_R	GGTGTCCCCCTCTGATACCT
CBS3_F	CTCAGCCACCACATGACCTT
CBS3_R	GGGAGTCCCATCACAGTGTC
CBS4_F	CCAGTCAGGGTCCATGTTGG
CBS4_R	GGTCTTCAAAGCCCTGTGGT
CBS5_F	CCACAAGCTGATGGGGTTGA
CBS5_R	ACCTGGAGGGAAGCTCAGAT
CBS6_F	CACCAAATCTCCCTCACCCC
CBS6_R	AATCTCCTTCACAGACGCCC
Neg_F	AAAGGCCACAAACTCCCCAT
Neg_R	ATTTGGCAGAGCTGAGCGTT
CBS7_F	CAGAACGATGACTGGACCGT
CBS7_R	GGCCGCTGTAAACACCAAAG
CBS8_F	CAGAACGATGACTGGACCGT
CBS8_R	GGCCGCTGTAAACACCAAAG

Cloning of active enhancer region

R2_F	ACTGAGCTAGCGCAGTGAGCTGAGATGTTGC
R2_R	ACTGACTCGAGATCTGGCAACTCCACTTTGG
R4_F	ACTGAGCTAGCGAGTGGTCTGTTGCAGTCAGC
R4_R	ACTGACTCGAGGGTTTCACCACATTGGCCAGG
R5_F	ACTGAGCTAGCGGACAGAGTAACCTTAAGACACAGG
R5_R	ACTGACTCGAGCCAGAGAGGTGATGAGTCTTGAGG
R6_F	ACTGAGCTAGCTGGAAGGACACAGAGCACAG
R6_R	ACTGACTCGAGTCTCTGCCTCCCTTTCTCAG
R7_F	ACTGAGCTAGCGCAGGTGAAGAAACTGAGGC
R7_R	ACTGACTCGAGAACGTACTCAAGCCCAGCAT
R8_F	ACTGAGCTAGCCCTGCTCCTTACAGCCTCAC
R8_R	ACTGACTCGAGCCCTGTTCTTTGCTGTCCTC
