# Supplementary material for: Chromatin 3D interaction analysis of the STARD10 locus unveils FCHSD2 as a regulator of insulin secretion
Source: Cell Rep. 2021 Feb 2;34(5):108703. doi: 10.1016/j.celrep.2021.108703 (PMC7856552; doi:10.1016/j.celrep.2021.108703)
Supplement: Document S2. Article plus supplemental information [file mmc2.pdf]

Cell Reports

Article

Chromatin 3D interaction analysis of the *STARD10* locus unveils *FCHSD2* as a regulator of insulin secretion

Graphical Abstract

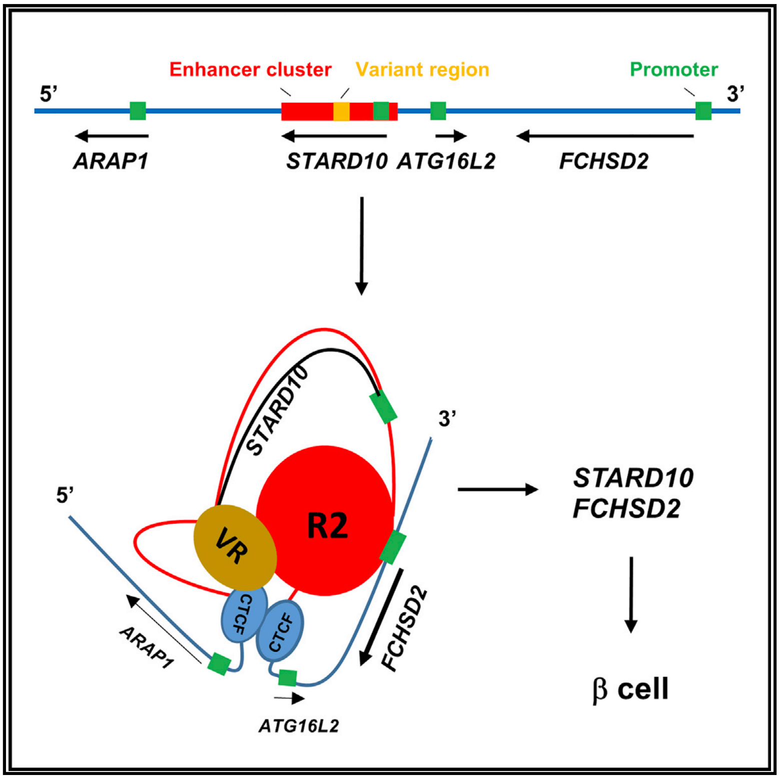

Highlights

- Type 2 risk variants at *STARD10* reside in CTCF-flanked enhancer clusters
- Loss of the risk variant-bearing region inhibits regulated insulin secretion
- 3D chromatin interaction maps reveal *FCHSD2* as target of the enhancer cluster
- Deletion of *STARD10* or *FCHSD2* from EndoC-βH1 β cells inhibits insulin secretion

Authors

Ming Hu, Inês Cebola, Gaelle Carrat, ..., Leonardo Alemeida-Souza, Harvey McMahon, Guy A. Rutter

Correspondence

g.rutter@imperial.ac.uk

In Brief

In this article, Hu et al. show the importance, for gene regulation, of a genomic region harboring GWAS variants that affect type 2 diabetes risk at the *STARD10* locus. They also identify FCHSD2 as a likely mediator of the effects of these variants on insulin secretion.

Hu et al., 2021, Cell Reports *34*, 108703
February 2, 2021 © 2021 The Author(s).
https://doi.org/10.1016/j.celrep.2021.108703

CellPress

Cell Reports

CellPress
OPEN ACCESS

Article

Chromatin 3D interaction analysis of the *STARD10* locus unveils *FCHSD2* as a regulator of insulin secretion

Ming Hu,[1] Inês Cebola,[2] Gaelle Carrat,[1] Shuying Jiang,[1] Sameena Nawaz,[3] Amna Khamis,[4] Mickaël Canouil,[4] Philippe Froguel,[4] Anke Schulte,[5] Michele Solimena,[6] Mark Ibberson,[7] Piero Marchetti,[8] Fabian L. Cardenas-Diaz,[9,10] Paul J. Gadue,[9,10] Benoit Hastoy,[3] Leonardo Alemeida-Souza,[11] Harvey McMahon,[12] and Guy A. Rutter[1,13,14,*]

[1]Section of Cell Biology and Functional Genomics, Department of Medicine, Imperial College London, Hammersmith Hospital, Du Cane Road, London W12 0NN, UK
[2]Section of Genetics and Genomics, Department of Metabolism, Digestion, and Reproduction, Imperial College London, Hammersmith Hospital, Du Cane Road, London W12 0NN, UK
[3]Oxford Centre for Diabetes Endocrinology and Metabolism, University of Oxford, Churchill Hospital, Headington, Oxford OX3 7LE, UK
[4]Université de Lille, CNRS, CHU Lille, Institut Pasteur de Lille, UMR 8199 - EGID, 59000 Lille, France
[5]Sanofi-Aventis Deutschland GmbH, 65926 Frankfurt am Main, Germany
[6]Paul Langerhans Institute of the Helmholtz Center Munich at the University Hospital and Faculty of Medicine, TU Dresden, 01307 Dresden, Germany
[7]Vital-IT Group, SIB Swiss Institute of Bioinformatics, 1015 Lausanne, Switzerland
[8]Department of Endocrinology and Metabolism, University of Pisa, 56126 Pisa, Italy
[9]Department of Pathology and Laboratory Medicine, University of Pennsylvania, Philadelphia, PA, USA
[10]Centre for Cellular and Molecular Therapeutics, Children's Hospital of Philadelphia, Philadelphia, PA, USA
[11]HiLIFE Institute of Biotechnology & Faculty of Biological and Environmental Sciences, University of Helsinki, Helsinki, Finland
[12]MRC MRC Laboratory of Molecular Biology, Francis Crick Avenue, Cambridge CB2 0QH, UK
[13]Lee Kong Chian School of Medicine, Nan Yang Technological University, Singapore, Singapore
[14]Lead contact
*Correspondence: g.rutter@imperial.ac.uk
https://doi.org/10.1016/j.celrep.2021.108703

SUMMARY

Using chromatin conformation capture, we show that an enhancer cluster in the *STARD10* type 2 diabetes (T2D) locus forms a defined 3-dimensional (3D) chromatin domain. A 4.1-kb region within this locus, carrying 5 T2D-associated variants, physically interacts with CTCF-binding regions and with an enhancer possessing strong transcriptional activity. Analysis of human islet 3D chromatin interaction maps identifies the *FCHSD2* gene as an additional target of the enhancer cluster. CRISPR-Cas9-mediated deletion of the variant region, or of the associated enhancer, from human pancreas-derived EndoC-βH1 cells impairs glucose-stimulated insulin secretion. Expression of both *STARD10* and *FCHSD2* is reduced in cells harboring CRISPR deletions, and lower expression of *STARD10* and *FCHSD2* is associated, the latter nominally, with the possession of risk variant alleles in human islets. Finally, CRISPR-Cas9-mediated loss of *STARD10* or *FCHSD2,* but not *ARAP1,* impairs regulated insulin secretion. Thus, multiple genes at the *STARD10* locus influence β cell function.

INTRODUCTION

Genome-wide association studies (GWAS) have identified >400 genetic signals across >200 loci that associate with type 2 diabetes (T2D) risk (Mahajan et al., 2014, 2018; Morris et al., 2012; Scott et al., 2017; Spracklen et al., 2020; Voight et al., 2010). Data from these and other studies indicate that islet dysfunction plays a major role in T2D genetic risk. However, most associated genetic variants lie in intergenic or intronic regions of the genome, but only a minority affect protein sequences (Fuchsberger et al., 2016).

One plausible mechanism by which genetic variants contribute to T2D risk is by affecting functional noncoding sequences. Consisting of short DNA regions and located at varying distances from promoter sequences, enhancers are *cis*-regulatory elements that promote the expression of target genes due to their co-occupancy by tissue-enriched transcription factors and coactivators. T2D GWAS variants are enriched within pancreatic islet enhancer clusters, also called clusters of open regulatory elements (COREs), stretch enhancers, super-enhancers and, more recently, enhancer hubs (Gaulton et al., 2010; Miguel-Escalada et al., 2019; Parker et al., 2013; Pasquali et al., 2014). Enhancer clusters often control temporal and cell-specific functions and define cell identity (Gosselin et al., 2014; Huang et al., 2016; Whyte et al., 2013). Thus, genetic variants in islet enhancer clusters may contribute to diabetes risk by

 © 2021 The Author(s).
This is an open access article under the CC BY license (http://creativecommons.org/licenses/by/4.0/).

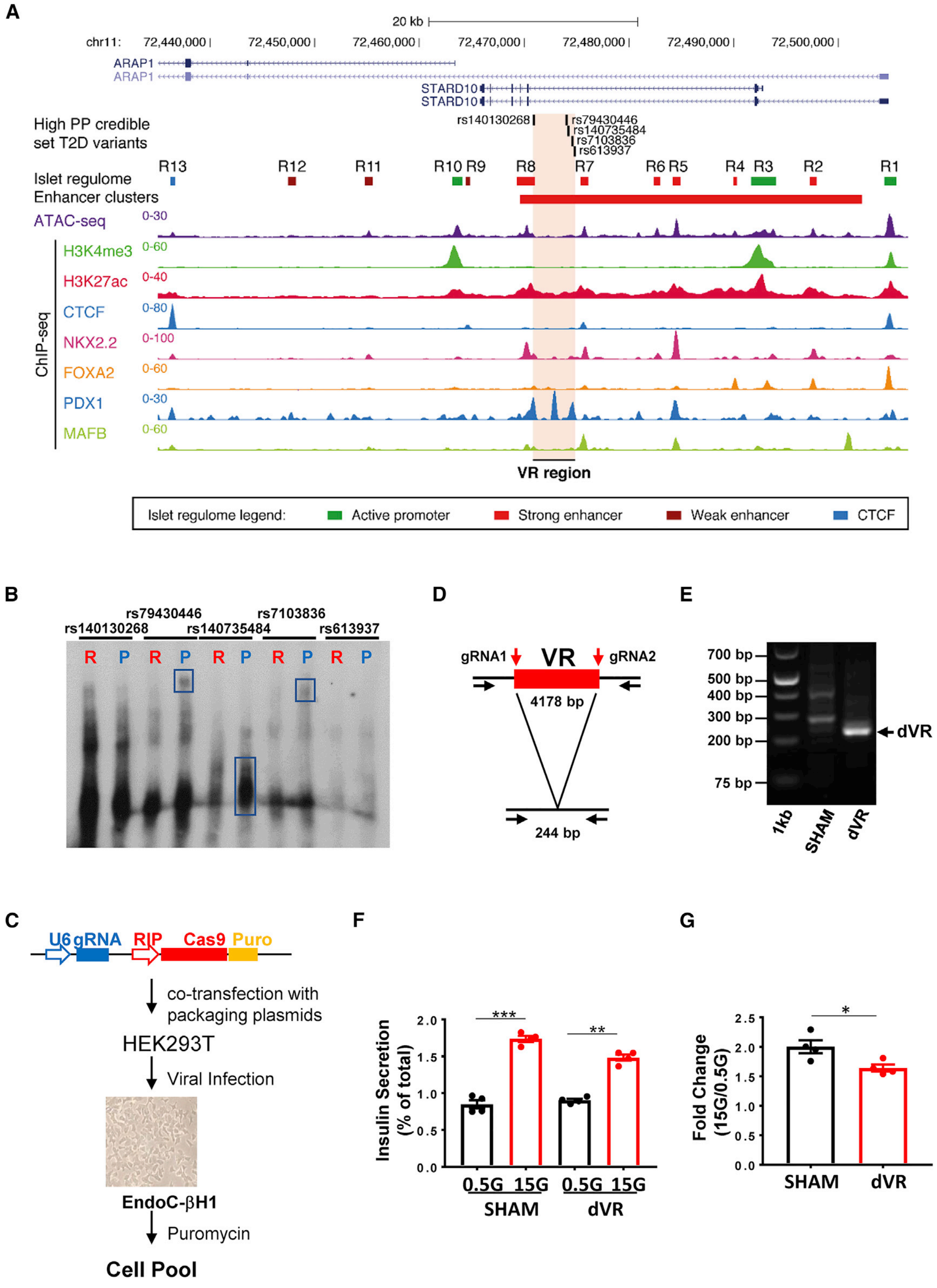

Figure 1. Variant region (VR) in local chromatin structure and β cell function

(A) Epigenomic map of *STARD10* locus in human islets. The open chromatin regions identified by ATAC-seq were as R for regulatory region. Enhancer cluster: solid red bar.

(B) Electrophoretic mobility shift assay (EMSA). R, risk allele; P, protective allele. n = 2.

(legend continued on next page)

perturbing islet transcriptional networks. Consequently, in addition to the identification of causal variants, functional characterization of enhancer-target gene(s) interactions, and of their effect(s) on β cell function, are required to fully understand the genetic influence of T2D pathogenesis.

Enhancers interact with target gene(s) to regulate their expression, an effect achieved through chromatin looping, often mediated by the highly conserved architectural protein CTCF (CCCTC-binding factor) (Bonev and Cavalli, 2016; Williams and Flavell, 2008). CTCF contains a DNA-binding domain that recognizes a non-palindromic motif. Highlighting the relevance of CTCF sites in chromatin architecture and gene regulation, deletion, or inversion of CTCF-binding sites (CBSs) can affect chromatin looping and cause dysregulated gene expression (Guo et al., 2015; Mandegar et al., 2016; de Wit et al., 2015).

In our recent studies (Carrat et al., 2017), we used functional GWAS (fGWAS) (Pickrell, 2014) to fine map a diabetes-associated credible set in the *STARD10* (StAR-related lipid transfer protein 10) T2D GWAS locus, in which the risk haplotype has a global frequency of 86%. The identified credible set is composed of 8 variants, 5 of which displayed a posterior probability >0.05, in intron 2 of the *STARD10* gene. One of these (indel rs140130268), which possessed the highest probability, is located at the edge of a region of open chromatin (assay for transposase-accessible chromatin using sequencing [ATAC-seq]). Whether and how these variants affect the expression of local or remotely located genes in human β cells were not, however, examined in our earlier report.

In the present study, we have used human EndoC-βH1 cells, which recapitulate many of the functional properties of native human β cells (Ravassard et al., 2011), and deployed chromatin interaction analyses and β-cell tailored clustered regularly interspaced short palindromic repeats (CRISPR)-endonuclease from *Streptococcus pyogenes* (Cas9) genome editing to explore this question. We show that the variant region (VR) is required for normal glucose-stimulated insulin secretion and identify the enhancer regions with which it interacts physically. We also demonstrate direct roles for *STARD10* in human-derived β cell function. Finally, we provide genetic and functional evidence of a role for a previously unimplicated nearby gene, *FCHSD2* (FCH and double SH3 domains protein 2), encoding a regulator of membrane trafficking and endocytosis (Almeida-Souza et al., 2018), in variant action.

RESULTS

Chromatin landscape at the *STARD10* locus

We investigated regulatory regions at the T2D GWAS locus close to *STARD10* by overlaying multiple human islet epigenomic datasets: ATAC-seq, histone marks associated with active chromatin (i.e., H3K27ac), and chromatin immunoprecipitation sequencing (ChIP-seq) for key islet transcription factors (TFs) (Miguel-Escalada et al., 2019; Pasquali et al., 2014). This analysis revealed multiple regulatory elements (R1–R13) that are active in human islets, including a cluster of 6 enhancers (Figure 1A). Several of these were bound by islet-enriched TFs such as NKX2.2, FOXA2, and MAFB, and are thus likely to contribute to an islet-specific gene expression signature. We also detected two binding sites for the chromatin architectural factor CTCF flanking the enhancer cluster, which may be involved in the creation of a distinct chromatin domain and mediate long-range looping with distal target genes (Figure 1A).

Credible set variants exhibit differential transcription factor binding and transcriptional activity

We next turned our attention to the five variants in the credible set with the greatest causal probability, as defined previously by fine mapping and fGWAS analysis (Carrat et al., 2017). These variants span a 4.1-kb interval and include 2 deletions (indels rs140130268 and rs140735484) and 3 single nucleotide polymorphisms (SNPs; rs79430446, rs7103836, and rs613937). Of these, indel rs140130268 displayed the highest posterior probability and showed allele-specific transcriptional activity in β cells (Carrat et al., 2017). Detailed epigenomic analysis (Figure 1A) mapped these variants to the center of an enhancer cluster defined by strong H3K27ac enrichment in islet chromatin (Pasquali et al., 2014), although none of them resided within a previously mapped open chromatin region. We note, however, that the risk haplotype in this particular locus, which associates with lowered regulatory activity (Carrat et al., 2017), is present in 86% of the human population. Thus, it is possible that the existing regulatory maps do not represent the epigenomic landscape of carriers of the non-risk haplotype.

A likely mechanism by which the risk haplotype could confer reduced local chromatin accessibility is via the alteration of TF recognition sequences. To test this hypothesis, we performed TF motif analysis (Khan et al., 2018) on these variants, which suggested that four of the five variants may affect TF binding to this enhancer cluster (Table S1). To further explore this possibility, we assayed allele-specific TF binding by electrophoretic mobility shift assays (EMSAs), with oligonucleotides carrying either risk or protective variants. While differences were modest between risk and protective alleles for rs140130268, we observed marked differences in DNA-protein complex mobility between risk and protective alleles for rs79430446, rs140735484, and rs7103836 (Figure 1B). These results therefore point to a potential regulatory function of the variants in this credible set.

(C) Diagram of CRISPR-Cas9-mediated genome editing with a β cell-tailored vector via lentiviral approach. Lentiviruses were generated in HEK293T cells, titrated, and used to infect EndoC-βH1 cells. Puromycin was used to select viral resistant cells and generate a cell pool.

(D) Strategy of VR deletion. Two gRNAs were designed to flank the VR region and generate a 4,178-bp deletion.

(E) Electrophoresis of PCR products amplified from SHAM and VR-deleted (dVR) genomic DNA. Note that the bands in the SHAM lane (~300–400 bp) were non-specific products.

(F) Representative data of glucose-stimulated insulin secretion (GSIS) assay. The experiment was performed in duplicate (n = 2) with insulin measurement in duplicate.

(G) Fold change of secreted insulin. Data are normalized to insulin secretion at basal level (0.5 mM). The experiments were repeated 4 times (n = 4).

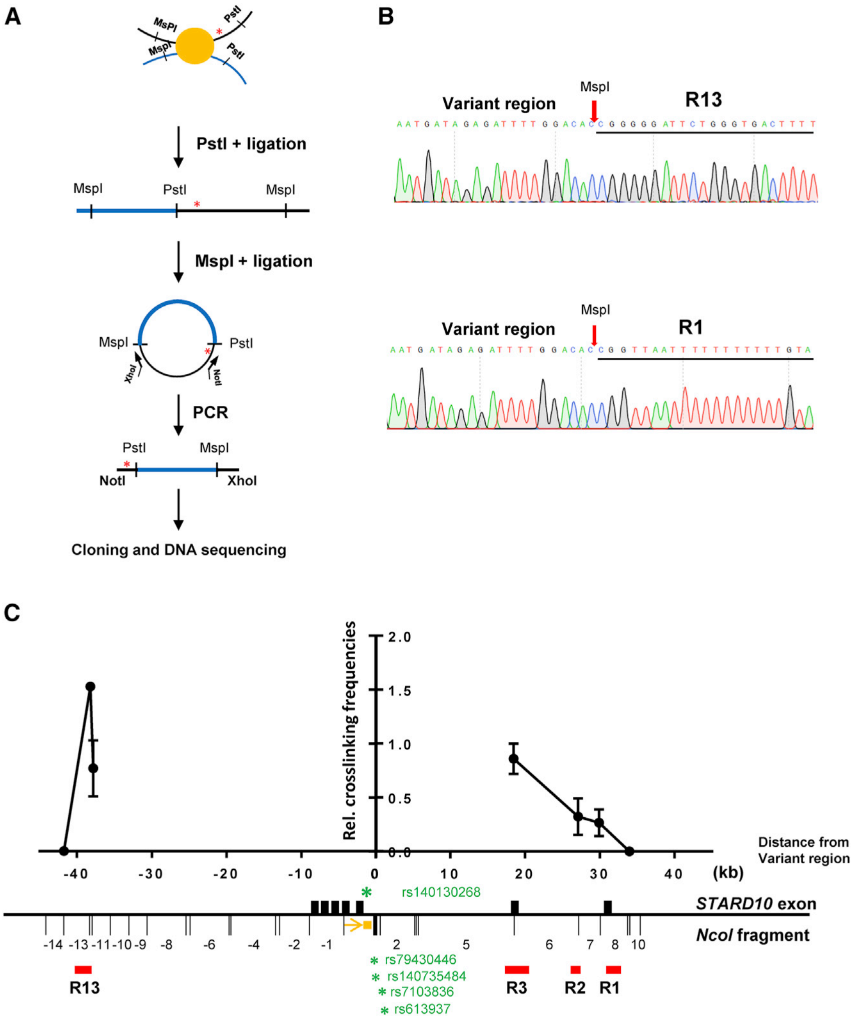

Figure 2. Identification and confirmation of genomic regions that physically interact with VR

(A) Diagram of conventional 4C assay.

(B) Representative DNA sequencing results showing ligation products between VR and interactive regions (R13 and R1 regions).

(C) Representative 3C-qPCR data for chromatin interactions at *STARD10* locus. Viewpoint: VR region; black bar: *STARD10* exons; red bar: regulatory regions; orange box: qPCR probe; orange arrow: qPCR constant primer; green stars: credible set genetic variants. The numbering of *NcoI* fragments is given relative to the viewpoint. The leftmost dot corresponds to the 5′ end of the −13 DNA fragment. Note that the bait fragment contains rs140130268 only. rs79430446 and rs140735484 are in *NcoI* fragment 1; rs7103836 and rs613937 are in *NcoI* fragment 2; R13 is in *NcoI* fragment −13 and −12, while R1 is in *NcoI* fragment +8. Data were normalized to a *CXCL12* loading control; n = 3.

Deletion of the VR from the EndoC-βH1 genome reduces insulin secretion

These 5 risk-bearing variants are located in a region between enhancers R7 and R8 (Figure 1A). This variant-containing region represents an area of open chromatin, as characterized by H3K27ac status and the binding of PDX1 (Figure 1A). To assess its importance, we deleted the entire 4.1-kb VR in EndoC-βH1 cells, which are homozygous for the risk haplotype, using CRISPR-Cas9-mediated genome editing (Figure 1C). To this end, we designed 2 guide RNAs (gRNAs) flanking the 4.1-kb genomic region that contains the T2D variants (Figure 1D). CRISPR-Cas9 genome editing was then performed in the glucose-responsive human β cell line EndoC-βH1 (Ravassard et al., 2011), delivering the gRNAs and the Cas9 gene under the control of the rat insulin promoter (RIP), and a puromycin resistance cassette. Deletion of the VR was confirmed by PCR (Figure 1E) and Sanger sequencing (Figure S2A). To control for possible non-specific effects of genome deletion and off-target CRISPR-Cas9 editing, we set up 2 controls, deleting regions of similar length in (1) an intergenic region between the *RAB6A* and *MRPL48* genes (Figures S1A and S2B) (this region is ~100 kb 3′ end of the *FCHSD2* promoter region) and (2) the β-globin locus (*HBB* gene) (Figures S1B and S2C). In addition, we used two scrambled gRNAs (Sc-gRNAs) that do not bind to the human genome as an additional control. gRNAs were designed using the Massachusetts Institute of Technology Genetic Perturbation Platform (MIT GPP) sgRNA designer platform (https://portals.broadinstitute.org/gpp/public/analysis-tools/sgrna-design) to minimize possible off-target sites.

Quantification by qPCR revealed that the remaining wild-type allele in dVR cells represented ~48% of the total. In addition, we detected DNA inversion after editing, corresponding to ~4.7% of the remaining wild-type alleles. Hence, the overall deletion efficiency was ~43% (Figures S3A–S3C). The deletion efficiencies for the intergenic region and the *HBB* region were 57.3% and 73.1%, respectively (Figures S3D–S3I).

To determine whether loss of the T2D variants may affect β cell function, we assayed glucose-stimulated insulin secretion (GSIS) in the presence (SHAM) or absence (dVR) of the VR. dVR cells displayed a small but significant reduction in GSIS (fold change: SHAM: 2.00 ± 0.11 versus dVR: 1.64 ± 0.06; p = 0.0267) (Figures 1F and 1G). In contrast, stimulation with neither glucose nor glucose plus the phosphodiesterase inhibitor isobutylmethylxanthine (IBMX) were altered in cell lines generated using Sc-RNAs or deleted for the *RAB6A-MRPL48* or *HBB* regions (Figures S3J and S3K).

The T2D credible set variants interact with active islet regulatory elements

The 4.1-kb VR that encompasses the 5 T2D credible set variants lies between 2 active enhancers (Figure 1A), but it does not overlap with any annotated islet regulatory element. Since the above

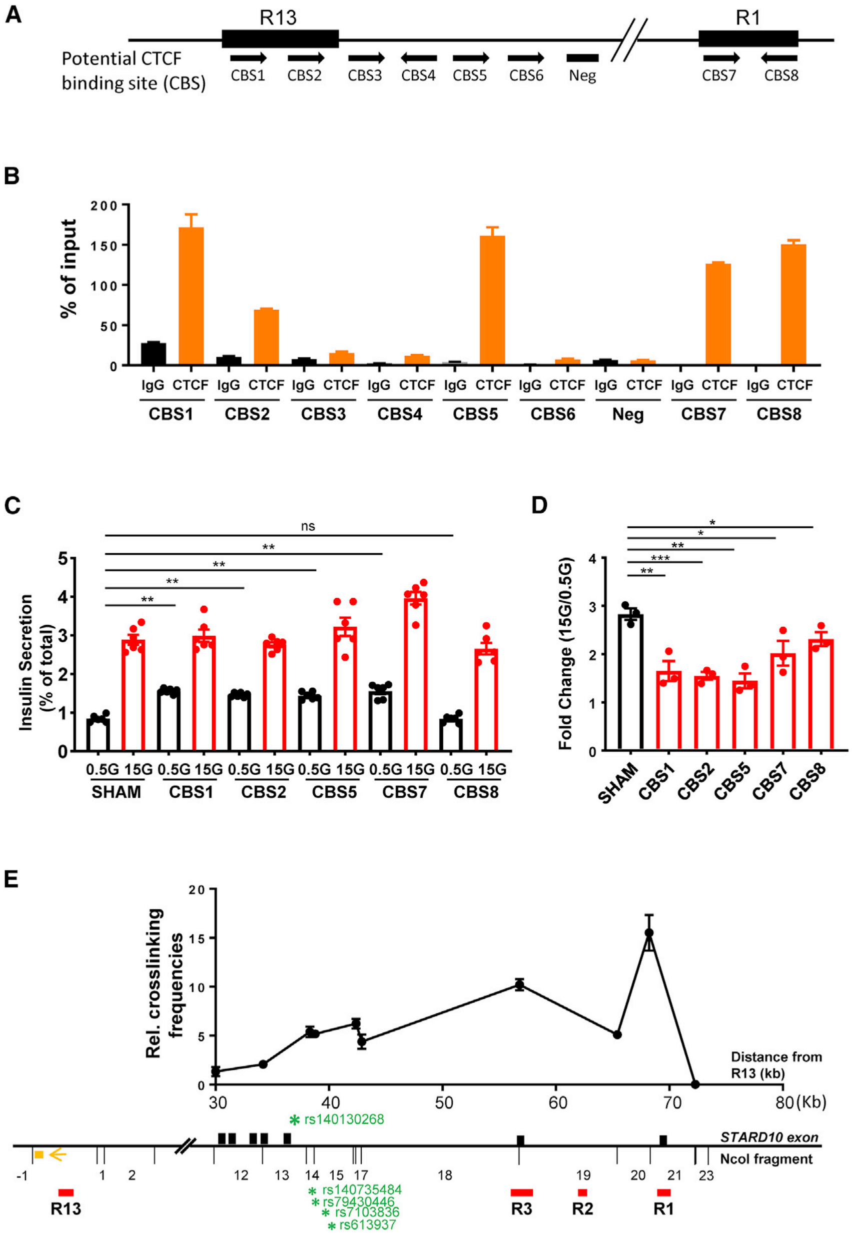

Figure 3. CTCF binding sites (CBSs) at R13 and R1 regions

(A) Diagram of potential CBS within and surrounding R13 and R1 regions. Black arrows: binding orientation of CTCF.

(B) Representative data of ChIP-qPCR analysis for CTCF binding at predicted binding sites; n = 3.

(C) Representative data of GSIS assay; n = 3.

(D) Fold change of secreted insulin. Data are normalized to insulin secretion at basal level (0.5 mM glucose); n = 3.

(E) Representative 3C-qPCR data of chromatin interactions at *STARD10* locus. The numbering of *NcoI* DNA fragments is given relative to the viewpoint. Viewpoint: R13 region; black bars: *STARD10* exon; red bars: regulatory region; orange box: qPCR probe; orange arrow: qPCR constant primer; green stars: credible set genetic variants. Note that R1 is in the *NcoI* fragment +21. Data were normalized to a *CXCL12* loading control; n = 3.

experiments demonstrated that this region is involved in the regulation of insulin secretion (Figures 1F and 1G), we hypothesized that it may contribute to β cell function by affecting chromatin topology and/or gene expression. To determine which genomic region(s) the VR may interact with, we performed circular chromosome conformation capture (4C) analysis (Göndör et al., 2008) (Figure 2A). Out of a total of 56 clones obtained after restriction enzyme digestion (PstI and *Msp*I) and DNA ligation, we detected 4 clones containing a DNA fragment within region R13, which corresponds to a strong CTCF site in human islets (Figure 2B). Moreover, 5 sequenced fragments mapped to a region 1.3 kb upstream of the R1 region, which contains 1 of the 2 promoters of *STARD10* that are active in human islets and is bound by CTCF (Figure 2B). The remaining fragments corresponded to DNA regions in the vicinity of the viewpoint, likely reflecting local chromatin collisions (Hagège et al., 2007).

To validate the 4C results above, we performed a 3C analysis (Figure 2C). Taking the T2D credible set variants region as a viewpoint, we detected higher interaction frequencies of the VR with both the CTCF site R13 and the 2 promoters of *STARD10* (R3 and R1). These results demonstrate that the T2D credible set variants region in *STARD10* undergo cis-interactions with human islet regulatory elements, including CTCF anchor points and the 2 promoters of *STARD10*.

Identification of CBSs at the *STARD10* locus

Inspection of human islet ChIP-seq datasets, together with *in silico* TF binding motif analysis (Figures 1A and S4A), revealed that both R1 and R13 contain binding sites for critical islet TFs, including NKX2.2 and FOXA2, indicating their potential role in the regulation of islet gene expression. Of note, the two regions also showed enrichment for CTCF by ChIP-seq in human islets (Figure 1A). Using the ChIP-qPCR assay in EndoC-βH1 cells, we detected the binding of CTCF to five of the eight potential CTCF binding motifs (Figures 3A and 3B). More important, we detected CTCF enrichment at convergent CTCF-binding motif sequences, a configuration that has been previously shown to be involved in chromatin looping (de Wit et al., 2015).

Mutation of CBSs leads to impaired insulin secretion

Higher-order chromatin structure is required for the regulation of cell-specific transcriptional activity (de Wit et al., 2015; Guo et al., 2015; Lupiáñez et al., 2015). Given the structural features

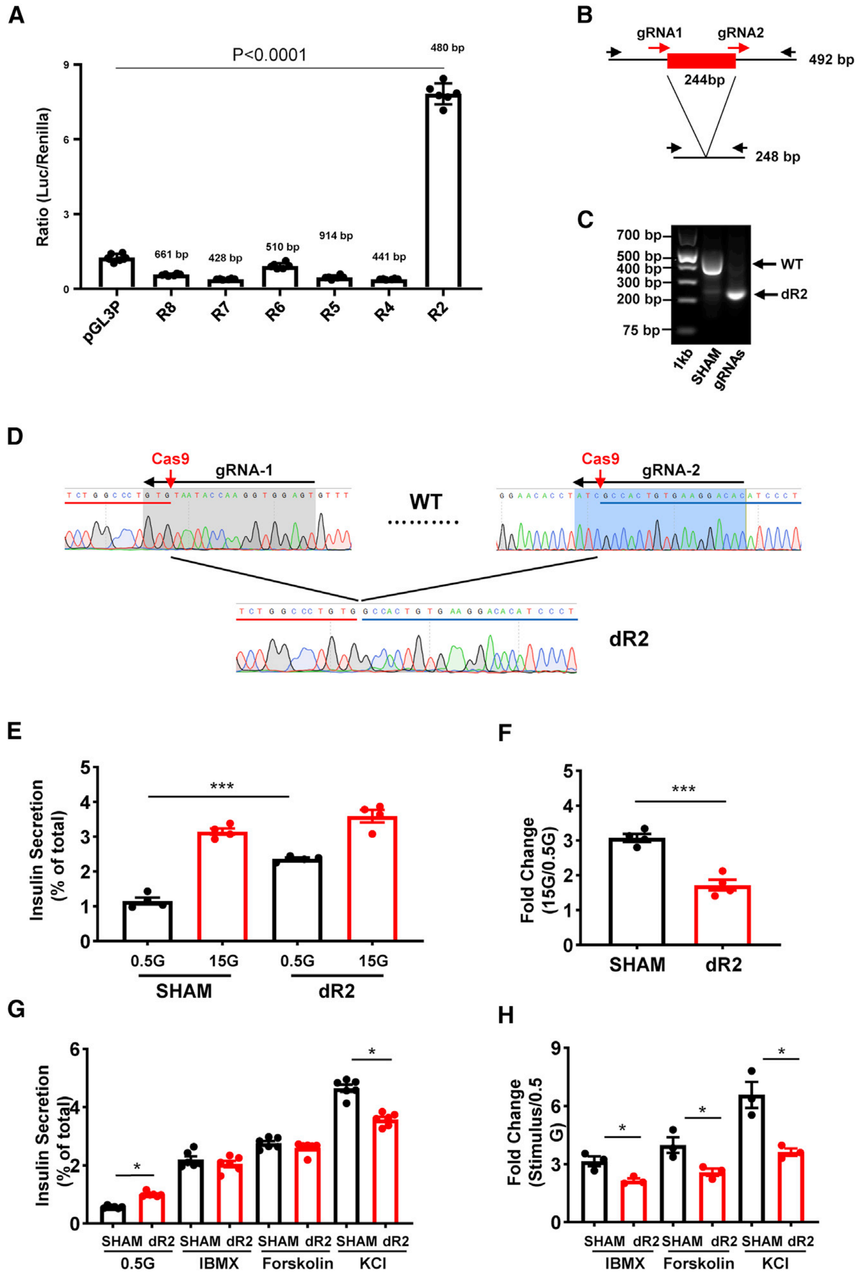

Figure 4. Role of enhancer R2 in insulin secretion

(A) Promoter-luciferase assay in EndoC-βH1 cells; n = 3.

(B) Strategy of R2 deletion by CRISPR-Cas9 genome editing. Two gRNAs were designed to delete 244 bp of the core R2 region.

(C) Gel electrophoresis of PCR products amplified from genomic DNAs isolated from control and R2-deleted (dR2) cells.

(D) Sanger sequencing confirmation of R2 deletion. Red and blue bars represent the 5′ and 3′ ends of the DNA sequence flanking the R2 region.

(E) Representative data of GSIS assay; n = 2

(F) Fold change of secreted insulin. Data are normalized to insulin secretion at basal level (0.5 mM glucose); n = 4.

(G) Representative data of insulin secretion stimulated by other stimuli; n = 3

(H) Fold change of secreted insulin. Data are normalized to insulin secretion at basal level (0.5 mM glucose); n = 3.

of the *STARD10* locus described above, we investigated whether loss of key architectural elements in the *STARD10* locus could lead to β cell function impairment. Using CRISPR-Cas9, we mutated these identified CBSs individually in EndoC-βH1 cells. ChIP-qPCR for CTCF confirmed the significant loss of CTCF-binding ability at designated binding sites after CRISPR targeting (Figure S4B). In assays of GSIS, we observed that CRISPR-Cas9-mediated targeting of 4 of the 5 CBSs in the region (CBS1, CBS2, CBS5, and CBS7) led to increased basal insulin secretion (at 0.5 mM glucose) and lowered the fold change in secretion at high (0.5 versus 15 mM) glucose (Figures 3C and 3D). Similar results were observed for insulin secretion stimulated by cyclic AMP (cAMP)-raising reagents such as IBMX and forskolin, as well as in response to cell depolarization with KCl (Figures S4C and S4D). Furthermore, we found that the expression patterns of *STARD10* and of nearby genes was significantly altered in CBS mutated cells. qRT-PCR analyses (Figure S4E) revealed that *STARD10*, *ATG16L2*, and *FCHSD2* were the most downregulated in CBS mutant cells, while *ARAP1*, the gene that resides nearest the T2D-associated credible set, was unaffected.

These results demonstrate that CTCF binding, through its likely impact on chromatin structure organization at the *STARD10* locus, is necessary to maintain normal β cell function.

R13 and R1 regions form chromatin loops via CBSs

CTCF, together with the Cohesin complex, plays an important role in the formation of higher-order chromatin structures and may act as an insulator or boundary between *cis*-regulatory elements and their target genes (Bell et al., 1999; Lupiáñez et al., 2015; Merkenschlager and Odom, 2013). As shown in Figure 2C, the region containing the T2D credible set in *STARD10* interacts with both R1 and R13 (and both of the latter contain bona fide CTCF convergent binding sites) (Figure 3A). We therefore hypothesized that the two regions may interact with each other, via the formation of a CTCF-CTCF loop, to establish a restricted chromatin domain. To test this hypothesis, we performed 3C analysis in EndoC-βH1 cells and explored the interaction frequency between R1 and R13. Taking R13 as the viewpoint, we detected interaction frequencies above background level across

CellPress
OPEN ACCESS

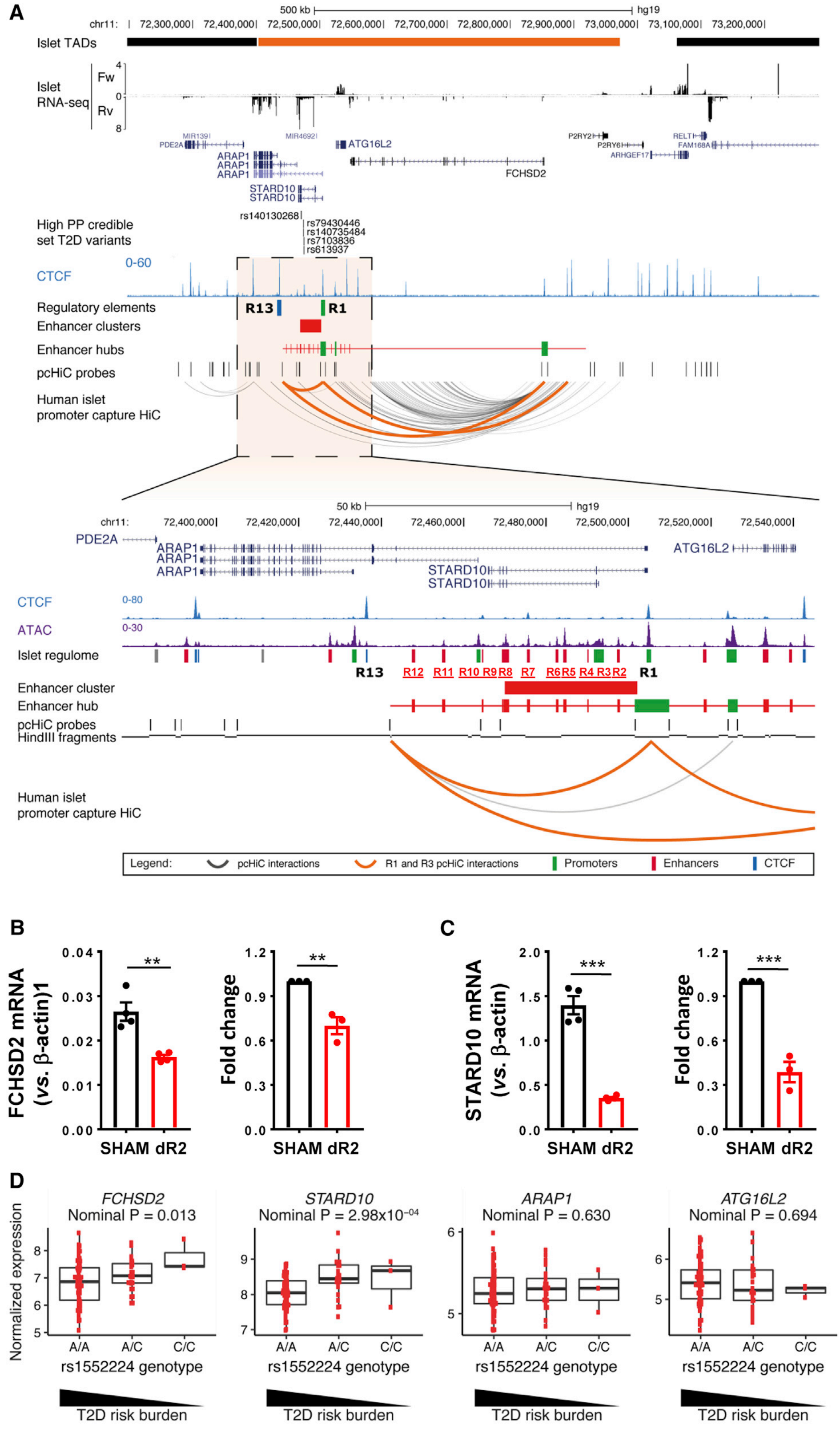

(legend on next page)

the entire *STARD10* locus (Figure 3E), particularly with the 2 promoters of *STARD10* (R3 and R1), but also with the T2D credible set region, as observed previously (Figure 2C). These observations confirm that the T2D VR interacts with a distal CTCF site in β cells and demonstrate that R13 and R1 are also associated through chromatin looping.

Screen of annotated genomic features reveals a functional islet enhancer that regulates basal insulin secretion

Since the T2D variants are deeply embedded within the region of active enhancers (Figure 1A), we hypothesized that the causal variants may exert their effect(s) by altering the activities of these enhancers. To explore this possibility, we sought first to understand the roles of the enhancer cluster in the control of β cell gene expression and function.

Regulome analyses of human islet samples, including ATAC-seq and ChIP-seq for H3K27ac, revealed 6 active enhancers showing islet TF binding (Figure 1A). We thus tested these regions by luciferase reporter assay in EndoC-βH1 cells, which revealed that R2 had a 6.25-fold activity increase compared with control ($p < 0.0001$) (Figure 4A). Other putative enhancers displayed negligible activity in this assay.

The R2 enhancer contains several recognition sequences for the binding of islet-associated TFs, such as FOXA2 and PAX4, suggesting a critical role in the regulation of nearby β cell genes (Figure S5A). To assess the role of this enhancer in β cell function, we deleted the core region of R2 (244 bp) from EndoC-βH1 cells using CRISPR-Cas9 (Figure 4B), achieving an ~85% loss of targeted alleles (Figures 4C and 4D and S5B–S5D). GSIS was significantly impaired in edited versus sham-treated cells ($p = 0.0004$) (Figures 4E and 4F), largely due to increased basal insulin secretion (0.5 mM glucose) ($p = 0.0004$). The stimulation of insulin secretion was even more sharply reduced in R2-deleted cells in response to cAMP-raising agents or after depolarization with KCl (Figures 5G and 5H).

Enhancer cluster regulates *FCHSD2* gene expression through chromatin looping

Individual enhancers can regulate multiple genes within the same cellular population, forming distinct 3-dimensional (3D) chromatin regulatory domains (“enhancer hubs”) (Miguel-Escalada et al., 2019; Oudelaar et al., 2018). We therefore postulated that the enhancer cluster may be part of a broader 3D chromatin domain in human islets.

To assess this possibility, we queried the recently published genome-wide map of human islet 3D chromatin interactions (promoter-capture HiC [pcHi-C]) (Miguel-Escalada et al., 2019). This analysis revealed that *STARD10* resides within an islet enhancer hub, together with *ARAP1*, *ATG16L2*, and the distal gene *FCHSD2*, located ~400 kb downstream of the *STARD10* enhancer cluster (Figure 5A). These four hub genes are expressed at relatively high to medium levels in human islets (see RNA-seq track in Figures 5A, and Figures S6A and S6B). We note, however, that *ARAP1* has three annotated promoters and one of them is included in the hub (Miguel-Escalada et al., 2019) (Figures S6A and S6B). The P1 promoter drives the expression of the longest isoform of *ARAP1* and is shared with *STARD10*. Examination of human islet RNA-seq revealed that the longer *ARAP1* transcript isoform from both P1 and P2 promoters displays much lower expression in human islets than the short isoform (Figure S4B). Thus, most *ARAP1* transcription is driven by promoter P3 (Figure S6B), which resides outside the *STARD10* islet enhancer hub.

To identify the gene(s) that are regulated by the nearby enhancer cluster, we carried out gene expression profiling in R2-deleted (dR2) cells. This revealed significant downregulation of only *FCHSD2* ($p = 0.0066$) and *STARD10* ($p = 0.0008$) (Figures 5B and 5C). In contrast, *ATG16L2* and *ARAP1*, the gene that was closest in linear distance to the enhancer cluster, were not affected by R2 deletion (Figures S6C and S6D). The latter observation is in line with our previous analysis of islet expression quantitative trait loci (eQTL), which did not reveal any association between the T2D variants in this locus and the expression of *ARAP1* (Carrat et al., 2017). Further analysis of the pcHi-C dataset confirmed that regions R1 and R13 interact in human islet chromatin (Figure 5A), consistent with our 3C analysis in EndoC-βH1 cells (Figure 3E). Moreover, the pcHi-C dataset revealed that the 2 CTCF binding sites that flank the *STARD10* enhancer cluster (R1 and R13) undergo long-range interactions with the promoter region of *FCHSD2* (Figure 5A).

Human islet eQTL

To gain insight into the relevance of our findings in the context of human islet physiology and diabetes risk, we analyzed the expression of *FCHSD2* in a cohort of 103 subjects who provided pancreatic samples after partial pancreatectomy and laser capture microdissection analysis (IMIDIA consortium; 47 non-diabetic, 56 T2D) (Solimena et al., 2018; Marchetti et al., 2018). We observed lower *FCHSD2* expression in carriers of the risk alleles of variants (rs75896506, rs11603334, and rs1552224; nominal $p = 0.013$), which are in high linkage disequilibrium (LD) with the high posterior probability variant rs140130268 (EUR $R^2 = 0.89$) (Figure 5D; Table S2). Consistent with earlier reports (Carrat et al., 2017; Miguel-Escalada et al., 2019), the risk variants also associated with lower *STARD10* levels in this cohort (nominal

Figure 5. Target genes regulated by the enhancer cluster

(A) Human islet promoter capture Hi-C (pcHi-C) map at *STARD10* locus and surrounding region. Orange interactions in the pcHi-C track depict interactions mediated by the CTCF sites that flank the enhancer cluster (R1 and R13). All other significant interactions are shown in dark gray. Enhancer hubs track shows all of the enhancers (red) and promoters (green) contained within the *STARD10* hub. Orange bar in islet topologically associating domains (TADs) track highlights the TAD encompassing *STARD10* and surrounding genes (*ARAP1*, *ATG16L2*, and *FCHSD2*).

(B and C) Taqman qRT-PCR analysis of gene expression in SHAM control and R2-deleted (dR2) cells.

(B) Representative data and fold change of *FCHSD2* gene; n = 3.

(C) Representative data and fold change of *STARD10* gene; n = 3.

(D) eQTL analysis of human islet samples. y axis represents normalized intensities (using robust multi-array average [RMA] method) from the Affymetrix Human Genome U133 Plus 2.0 Array; 203 total human samples.

CellPress
OPEN ACCESS

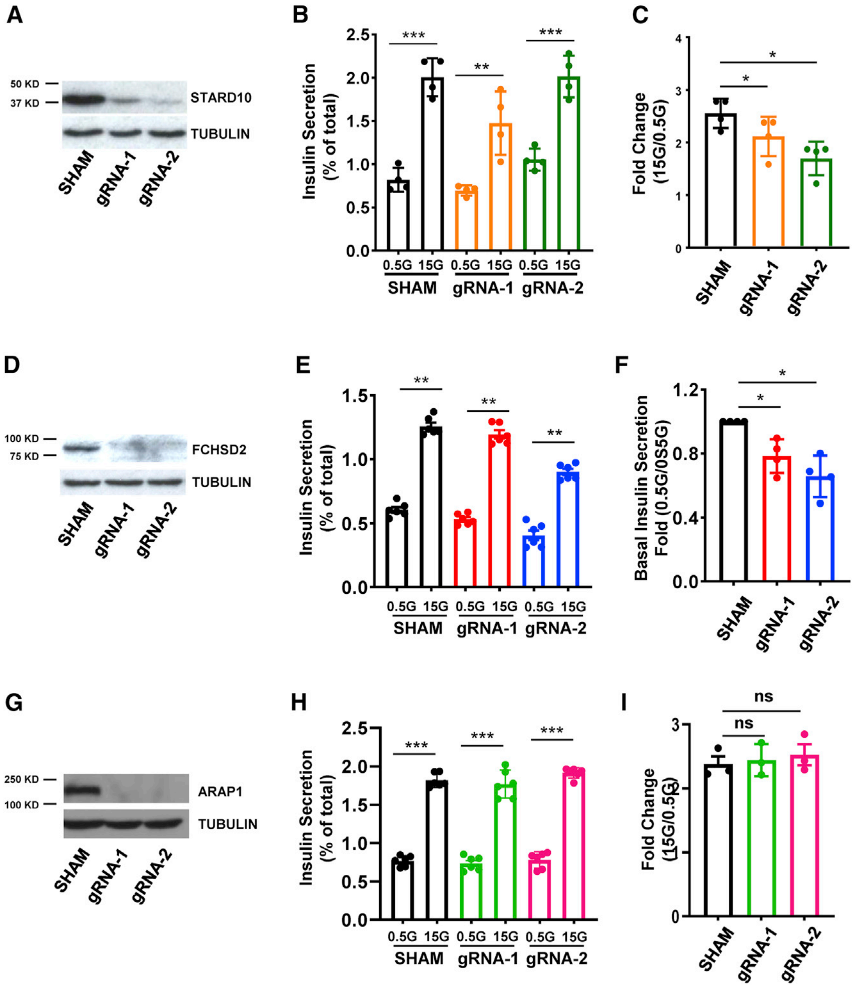

Figure 6. Effect of CRISPR/Cas9-mediated knockout of *STARD10*, *FCHSD2*, or *ARAP1* on basal and regulated insulin secretion

(A–C) Effect of *STARD10* knockout.

(A) Western blot assay.

(B) Representative data of GSIS assay; n = 2.

(C) Fold change of secreted insulin. Data are normalized to insulin secretion at basal level (0.5 mM glucose); n = 4.

(D–F) Effect of *FCHSD2* knockout.

(D) Western blot assay.

(E) Representative data of GSIS assay; n = 3.

(F) Fold change of secreted insulin at basal level. Data are normalized to basal insulin secretion (0.5 mM versus 0.5 mM); n = 4.

(G–I) Effect of *ARAP1* knockout.

(G) Western blot assay.

(H) Representative data of GSIS assay; n = 3.

(I) Fold change of secreted insulin. Data are normalized to insulin secretion at basal level (0.5 mM glucose); n = 3.

$p = 2.98 \times 10^{-4}$) (Figure 5D; Table S2). In contrast, no eQTL was found for *FCSHD2* in organ donor (OD)-obtained samples from the same cohort (p = 0.89), while the signal for *STARD10* remained nominally significant ($p = 2.19 \times 10^{-3}$). These results suggest that the T2D-associated variants in the *STARD10* enhancer hub selectively associate with differential expression of *STARD10* and *FCHSD2*.

Deletion of *STARD10* and *FCHSD2*, but not *ARAP1*, affect regulated insulin secretion in a human β cell line

We have previously demonstrated the importance of the *STARD10* gene but not *ARAP1* in regulating insulin processing and secretion in the mouse (Carrat et al., 2017). The findings above suggest that *FCHSD2* may also play a role in the control of insulin secretion. To further explore the potential roles of these genes in controlling insulin secretion in the human setting, we deployed CRISPR-Cas9 gene editing in EndoC-βH1 cells to generate frameshift mutations in exon 2 of *STARD10*, exon 1 of *FCHSD2*, and exon 3 of *ARAP1* (Figures S7A–S7C). Western blot analysis confirmed the expected loss of protein expression with 80%–90% efficiency for STARD10 (Figure 6A), >95% for both FCHSD2 (Figure 6D) and ARAP1 (Figure 6G).

For STARD10 null EndoC-βH1 cells, we observed a significant reduction in glucose-stimulated insulin secretion in comparison with control (gRNA1: p = 0.007 and gRNA2: p = 0.007) (Figures 6B and 6C), which is in agreement with our previous observations in mouse models (Carrat et al., 2017). Insulin secretion at low (0.5 mM) glucose was not affected by STARD10 loss. However, FCHSD2 null cells showed a relatively mild reduction in insulin secretion at both basal (gRNA1: p = 0.0265 and gRNA2: p = 0.013) (Figures 6E and 6F) and high glucose conditions (gRNA1: p = 0.0043 and gRNA2: p = 0.028) (Figure S7D), although no significant changes were observed when comparing the high and low glucose conditions (gRNA1: p = 0.97 and gRNA2: p = 0.75) (Figure S7E). Furthermore, *ARAP1* null cells, in agreement with previous observations in a mouse model (Carrat et al., 2017), did not show any significant changes in GSIS (gRNA1: p = 0.52 and gRNA2: p = 0.75) (Figures 6H and 6I).

FCHSD2 has been shown to regulate F-actin polymerization, suggesting it could be involved in insulin exocytosis (Cao et al., 2013). To identify any impact of *FCHSD2* loss of function on late events in insulin secretion, we next explored the regulation of secretion in response to depolarization with KCl in two further lines (KO1 and KO2) deleted for *FCHSD2,* compared to control lines generated using either Sc-gRNA or a gRNA that induces double-strand DNA breaks (Figure S8A). While no differences were apparent between the control lines (Figures S8B and S8C), both of the FCHSD2 null lines demonstrated the expected lowering in both basal (0.5 mM glucose) and stimulated (15 mM glucose)-induced secretion, but no change in the fold stimulation of secretion prompted by high glucose (15 versus 0.5 mM glucose) or by KCl (Figures S8D and S8E).

We further explored regulated exocytosis in *FCHSD2*-KO lines using sensitive electrophysiological measurements of cell

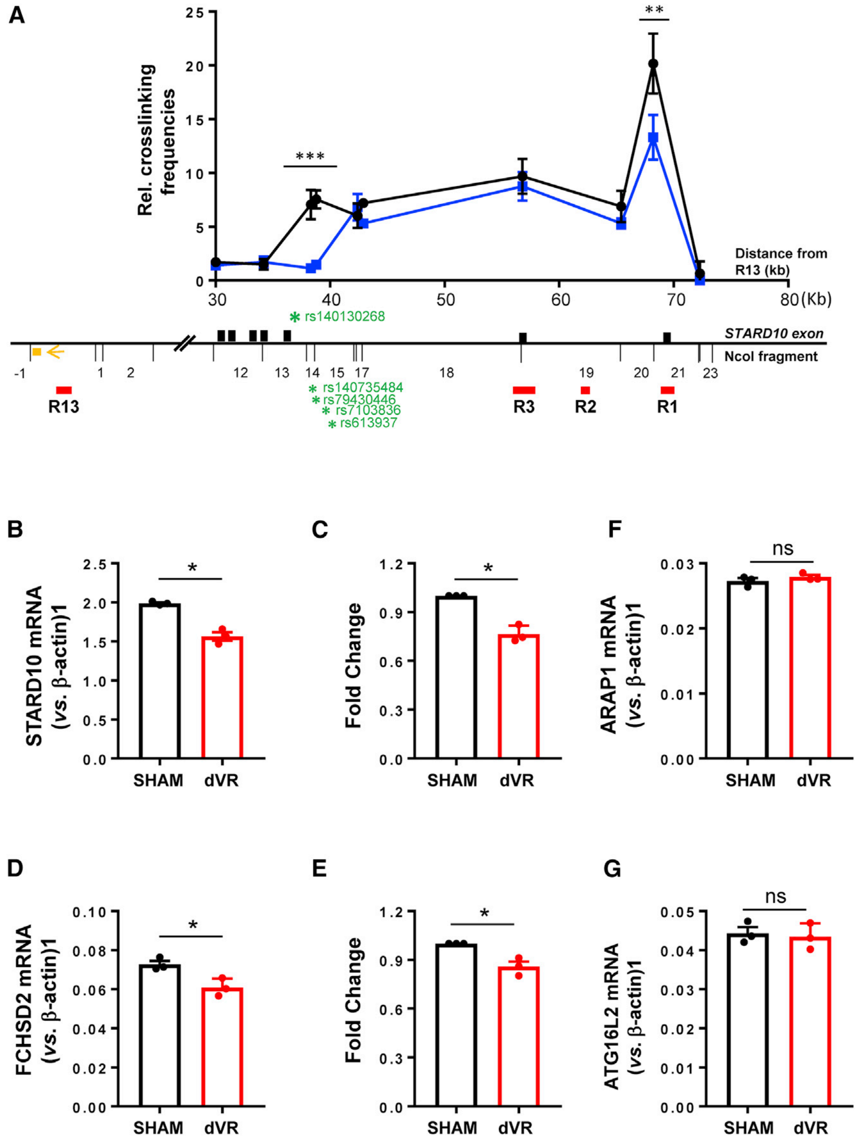

Figure 7. Effect of VR deletion on 3D structure and gene expression

(A) Combined 3C-qPCR data of chromatin interactions in dVR cells. Black: control; blue: dVR. The numbering of *NcoI* DNA fragments is given relative to the viewpoint. Viewing point: R13 region; black bars: *STARD10* exon; red bars: regulatory region; orange box: qPCR probe; orange arrow: qPCR constant primer; green stars: credible set genetic variants. Data were normalized to a *CXCL12* loading control; n = 3.

(B–G) Taqman qRT-PCR analysis of gene expression in SHAM control and dVR cells. (B and C) *STARD10*; (D and E) *FCHSD2*; (F) *ARAP1*; and (G) *ATG16L2*. (B) and (D) show relative expression levels (representative experiments); (C) and (E) indicate the fold change of expression; n = 3.

membrane capacitance (Figures S9A–S9E). FCHSD2 loss of function had no impact on cell size or on calcium current densities at 0 mV (Figures S9A and S9B). The cells were subjected to 10 depolarizations (pulses) from −70 to 0 mV (Figure S9C). The amplitude of exocytosis in *FCHSD2*-KO lines reached at pulse 10 was not significantly different between the lines (SHAM versus KO1 $p_{adjusted}$ = 0.108, SHAM versus KO2 $p_{adjusted}$ = 0.851) (Figure S9D). The cumulative increases in membrane capacitance were 10.89 ± 7.22 fF.pF^{-1} (n = 11) and 21.57 ± 3.56 fF.pF^{-1} (n = 13) for control lines and 27.29 ± 12.28 fF.pF^{-1} (n = 9) and 13.36 ± 7.48 fF.pF^{-1} (n = 12) for KO1 and KO2 lines, respectively. As observed previously (Hastoy et al., 2018), the increase in membrane capacitance was biphasic, with the majority of exocytosis elicited during the first two pulses (Figure S9E). No significant differences were observed in the increment triggered by the first pulse control and *FCHSD2*-KO lines (SHAM versus KO1 $p_{adjusted}$ = 0.108, SHAM versus KO2 $p_{adjusted}$ = 0.326). Thus, the increments equated to 3.36 ± 2.36 fF.pF^{-1} (n = 11) and 10.44 ± 7.04 fF.pF^{-1} (n = 13) for SHAM and Sc-gRNA lines, respectively, and to 12.10 ± 8.86 fF.pF^{-1} (n = 9) and 8.28 ± 7.21 fF.pF^{-1} (n = 12) for *FCHSD2*-KO1 and *FCHSD2*-KO2 lines, respectively. These findings argue against a direct role for FCHSD2 in controlling distal events in Ca^{2+}-regulated secretory granule exocytosis.

Deletion of the VR alters 3D chromatin structure and downregulates the expression of *STARD10* and *FCHSD2* genes

Finally, having examined the 3D structure and downstream genes of the enhancer cluster, we attempted to determine whether the risk-bearing VR may affect enhancer cluster function. As shown in Figure 1A, the VR is located between 2 active enhancers, R7 and R8, and, most important, is associated with CTCF-binding regions R13 and R1 through chromatin looping (Figure 2C). These observations suggest that the VR may be involved in the formation of local chromatin structure and thus in controlling the activity of the enhancer cluster. To test this possibility, we performed 3C-qPCR analysis in sham control and dVR cells. To increase the deletion efficiency of CRISPR-Cas9 editing in these experiments, we doubled the concentration of lentivirus (MOI = 20) and achieved ~80% deletion (Figures S10A and S10B). As shown in Figure 7A, VR deletion caused a significant change in 3D structure, notably a reduction in the physical interaction between the R13 and R1 regions (p = 0.0064). Further analysis of gene expression by qRT-PCR in dVR cells revealed that both the *STARD10* and *FCHSD2* genes were moderately downregulated when compared with sham control cells (*STARD10*: p = 0.015 and *FCHSD2*: p = 0.011). No significant change was observed in the expression of the

ARAP1 or *ATG16L2* genes (Figures 7B–7G). These data demonstrate that the VR is an important region regulating chromatin 3D structure and transcriptional activity of the enhancer cluster.

DISCUSSION

Our results demonstrate the involvement of STARD10 in insulin secretion in human β cells and unveil FCHSD2 as a previously unknown regulator of this process.

Our earlier study (Carrat et al., 2017) provided evidence, based on a combination of approaches, that *STARD10* is a critical mediator of the effects of the T2D-associated variants at this locus. However, our previous report did not explore in detail how the identified credible set may influence the expression of local genes, nor did it exclude the possibility that other genes may also be involved in the actions of risk variants.

The goals of the present study were, therefore, to obtain a more detailed molecular picture of the local chromatin structure at the *STARD10* T2D locus and to use this to identify and study the genes likely to interact with previously identified T2D risk variants in human β cells. In this way, we report a likely spatial organization, defined by CTCF-stabilized looping, which allows an enhancer cluster to regulate not only the *STARD10* gene but also the distal *FCHSD2* gene, which is contained in the same 3D chromatin compartment as *STARD10* and the T2D risk variants. In contrast, and consistent with our earlier studies (Carrat et al., 2017), we found no evidence for a role for *ARAP1* in mediating the effects of the variants on β cell function.

We also report here that variants of the risk haplotype are eQTLs for both *STARD10* and *FCHSD2* in human islets (note that data were not available for the indel with the highest posterior probability). More important, these results were only obtained in laser capture microdissection (LCM) donors from partially pancreatectomized patients (PPPs) from the ‘‘IMIDIA’’ dataset (Carrat et al., 2017). In contrast, interrogation of OD samples in the same dataset failed to reveal a nominal association between any of the T2D risk SNPs and *FCHSD2* expression. Moreover, interrogation of the other islet eQTL datasets reported earlier, which are also derived from OD samples (Fadista et al., 2014; Miguel-Escalada et al., 2019), provided no evidence of association with islet *FCHSD2* levels.

We note that the use of LCM to extract mRNA is unlike the enzymatic digestion methods that are commonly used with OD samples. Thus, surgical specimens are subject to immediate cryo-fixation, limiting RNA degradation and hence transcriptomic changes, which can occur in OD samples in which islets are isolated from the rest of the pancreas before mRNA extraction. Moreover, LCM methods provide a purer and more β cell-enriched cell population (Solimena et al., 2018). Correspondingly, OD and LCM samples from the same individual cluster separately (Solimena et al., 2018).

We suspect that the use of this dataset (Solimena et al., 2018) was critical to revealing an association between T2D risk variants at the *STARD10* locus, and the levels of expression of the *FCHSD2* gene. We note that this eQTL only achieved nominal association in a targeted analysis of the locus, but did not reach genome-wide significance, and that the additional human islet eQTL datasets we interrogated in this study (Fadista et al., 2014; Miguel-Escalada et al., 2019) only reported genome-wide significant eQTLs. These additional islet eQTL datasets were derived from OD samples. It is possible, therefore, that β cell-specific effects may be masked by ‘‘contaminating’’ signals from islet non-β and other cells. Supporting this view, interrogation of OD data from both the IMIDIA dataset (Solimena et al., 2018) and datasets from the Parker (Varshney et al., 2017) and Groop (Fadista et al., 2014) labs, and from Groop and Ferrer combined (Miguel-Escalada et al., 2019), as well as more recent OD data (Viñuela et al., 2020), fail to reveal a nominal association between SNP rs1552224 and *FCHSD2* expression (based on the index SNP). Interrogation of a very recent dataset involving 26 normoglycemic subjects in which islets were isolated from ODs and, subsequent to islet dispersal, purified by fluorescence-activated cell sorting (FACS) (Viñuela et al., 2020), demonstrated nominal eQTLs for rs1552224 and *STARD10* (7/7 exons), *PDE2A* (15/35 exons), *ARAP1* (1/9 exons), and *FAM168A* (1/9 exons) as well as *FCHSD2* (2/22 exons) at borderline ($p = 0.05$) significance or non-significance ($p = 0.06$). These data may further support the view that enhanced β cell purity contributes to the detection of an eQTL for *FCHSD2* in the LCM, but not OD (whole islet), data.

We used a CRISPR-Cas9 approach to delete the whole of the 4.1-kb VR likely to host the credible set of T2D variants with significant posterior probability at this locus (Carrat et al., 2017). This region was also identified as a credible set in earlier reports (Bonàs-Guarch et al., 2018; Gaulton et al., 2015; Mahajan et al., 2018), but did not feature in a recent report (Mahajan et al., 2018). Unlike our previous analysis, the new report from Mahajan et al. (2018) does not include indels (a Haplotype Reference Consortium [HRC] panel was used that included only SNPs). Thus, the variant in the VR with the highest PPA (indel rs140130268), as described in our earlier report (Carrat et al., 2017) in which a Metabochip scaffold was imputed up to a 1000 Genomes reference panel including indels, was not included. Whereas indel rs140130268 showed a PPA of 45% (Carrat et al., 2017), in Mahajan et al. (2018), maximum PPA is assigned to rs7109575 (PPAg [genetic credible set] = 0.14 [14%] and PPAf [functional credible set] = 0.38 [38%]). These two SNPs are in very high LD (EUR R^2 = 0.95). In any case, the discordance between earlier studies emphasizes the need for direct interventional studies in disease-relevant cells, as presented here.

While the ideal design of such studies would involve using homologous recombination to convert risk into protective alleles (or vice versa) one by one, currently available cellular systems largely preclude this. EndoC-βH1 cells grow slowly and do not tolerate single cell cloning, while human embryonic stem cell (ESC)-derived β cells (Nair et al., 2019; Pagliuca et al., 2014; Rezania et al., 2014) do not reliably provide a robust platform for functional studies. Although the approach adopted here to delete the whole 4.1-kb region in EndoC-βH1 cells provides no information on the role of the individual variants, it does allow us to suggest that this region is important for the control of local gene expression and β cell function.

Enhancer hub and chromatin structure at the *STARD10* locus

The spatial organization of chromatin can play an important role in gene regulation (Vernimmen and Bickmore, 2015). We found that the T2D risk variants at the *STARD10* locus reside in an islet enhancer hub that contains the genes *ARAP1*, *STARD10*, *ATG16L2*, and *FCHSD2*. Of note, the expression of *ARAP1* in human islets is chiefly driven by two promoters that reside outside the enhancer hub (Figures 5A and S4C) and is thus not likely to be co-regulated with *STARD10* and other hub genes, in line with evidence from eQTL studies (this report and Carrat et al., 2017).

Of note, we demonstrate that two CBSs exist at either end of the cluster, possibly creating a spatially organized transcriptional complex that is likely to influence the expression of relevant genes (Figure 1A).

STARD10 and *FCHSD2* mediate altered β cell function and disease risk

Our previous study (Carrat et al., 2017) provided evidence of an essential role for *STARD10* in mediating glucose-induced insulin secretion and proinsulin processing in the mouse. Here, using CRISPR-Cas9-mediated inactivation, we demonstrate that STARD10 is equally important in human β cells. In contrast, deletion of *ARAP1* from human EndoC-βH1 cells had no effect on basal or glucose-stimulated secretion, in line with our earlier study in the mouse (Carrat et al., 2017) and further arguing against a role for this gene in mediating the effects of risk genes at this locus. In addition, we failed to obtain any evidence for an impact of the disease-associated variants on *ARAP1* expression in human islets. We note, however, that the Affymetrix probes used (U133, Plus 2.0) detect exons common to all isoforms, including the low-abundance long transcript expressed from the P1 in the enhancer hub, as well as from promoter P2 (Figures 5A, S4C, and S4D). We are therefore unable to exclude the possibility that this transcript may be affected by the variants, although the impact, if any, of such a change is unclear.

Although the molecular roles for STARD10 in the β cell remain to be elucidated, our recent findings (Carrat et al., 2020) indicate a role in the control of secretory granule biogenesis. Analysis of human islet pcHiC (Miguel-Escalada et al., 2019) and measurements of changes in gene expression after deletion of R2 region or the core of the VR provide evidence that *FCHSD2* may also play a role in mediating disease risk. Interestingly, deletion of the VR led to a lowering of stimulated insulin secretion, but no change in basal insulin secretion (Figures 1F and 1G). Whereas the knockout of *FCSHD2* lowered both basal and stimulated insulin release (Figures 6H and S7D), the loss of *STARD10* had no effect on insulin secretion at low glucose, but it did impair insulin secretion at high glucose (Figures 6C and 6D). It may, therefore, be speculated that the VR exerts effects on insulin secretion via a combined action on both genes.

By what mechanisms might FCHSD2 affect insulin secretion? FCHSD2 has been shown to regulate F-actin polymerization (Cao et al., 2013) and act as a positive regulator of clathrin-mediated endocytosis (Almeida-Souza et al., 2018). More important, impaired clathrin-mediated endocytosis, achieved by the inactivation of dynamin-2 in β cells, impairs insulin exocytosis (Fan et al., 2015).

FCHSD2 is recruited to clathrin-coated pits by interaction through its second HS3 domain (SH3-2), while its first SH3 domain (SH3-1) binds to N-WASP to initiate F-actin polymerization (Almeida-Souza et al., 2018; Xiao et al., 2018). Exocytosis requires F-actin-mediated cytoskeletal remodeling (Jewell et al., 2008; Kalwat and Thurmond, 2013) in which N-WASP and the ARP2/3 complex are important protein complexes in the formation of focal adhesion. By affecting these processes, FCHSD2 may play an active role in the regulation of late events in secretion. However, measurements of exocytosis using sensitive capacitance recordings (Figures S9A–S9E) failed to provide direct evidence for such a role. Instead, we speculate that FCHSD2 regulates more proximal events, such as the recruitment of granules to a "reserve" pool (Rorsman and Renström, 2003) or the recycling of receptors (e.g., those for glucagon-like peptide-1 [GLP1]) (Jones et al., 2018).

Possible impact of T2D variants on chromatin landscape at the *STARD10* locus

We show that the VR region is located between two enhancers (R7 and R8) that are occupied by β cell-specific TFs such as PDX1 (Figure 1A). We also demonstrate that the VR interacts with CBSs and that its deletion causes a significant change in 3D structure, affecting the expression of the downstream genes *STARD10* and *FCHSD2* (Figure 7).

We also reveal that deleting a genomic region hosting variants associated with T2D affects chromatin structure. Future studies, in more tractable systems (e.g., CRISPR-edited human embryonic stem cell-derived β-like cells) are likely to be required to determine the effect of more targeted (e.g., single variant, haplotype) changes on local DNA structure.

In summary, the present report extends our previous study of the *STARD10* locus (Carrat et al., 2017), revealing important aspects of the control of the local chromatin structure and identifying a functionally relevant new gene, *FCHSD2*.

STAR★METHODS

Detailed methods are provided in the online version of this paper and include the following:

- KEY RESOURCES TABLE
- RESOURCE AVAILABILITY
    - Lead contact
    - Materials availability
    - Data and code availability
- EXPERIMENTAL MODEL AND SUBJECT DETAILS
- METHOD DETAILS
    - Electrophoretic mobility shift assay (EMSA)
    - Chromatin conformation capture (3C and 4C)
    - Chromatin immunoprecipitation
    - PCR and qPCR
    - Molecular cloning
    - Transfection and luciferase assay
    - CRISPR-Cas9-mediated genome editing
    - Electrophysiology
    - Insulin secretion
    - Human islet regulome and interactome analysis

  ○ Transcription factor binding motif analysis
  ○ Expression quantitative trait loci (eQTL) analysis
- QUANTIFICATION AND STATISTICAL ANALYSIS

SUPPLEMENTAL INFORMATION

Supplemental Information can be found online at https://doi.org/10.1016/j.celrep.2021.108703.

ACKNOWLEDGMENTS

G.A.R. was supported by Wellcome Trust Senior Investigator (WT098424AIA) and Investigator (212625/Z/18/Z) awards; Medical Research Council (MRC) Programme grants (MR/R022259/1, MR/J0003042/1, and MR/L020149/1); Experimental Challenge Grant (DIVA, MR/L02036X/1); MRC (MR/N00275X/1); Diabetes UK grants (BDA/11/0004210, BDA/15/0005275, and BDA 16/0005485); Imperial Confidence in Concept (ICiC) grants; and a Royal Society Wolfson Research Merit Award. I.C. has been awarded an Academy of Medical Sciences Springboard Fellowship (SBF005\1050), which is supported by the British Heart Foundation, Diabetes UK, the Global Challenges Research Fund, the Government Department for Business, Energy, and Industrial Strategy, and the Wellcome Trust. I.C. was also supported by the National Institute for Health Research (NIHR) and Imperial Biomedical Research Centre. B.H. thanks Diabetes UK for the R.D. Lawrence Fellowship (BDA 19/0005965). This project has received funding from the European Union's Horizon 2020 research and innovation programme via the Innovative Medicines Initiative 2 Joint Undertaking under grant agreement no. 115881 (RHAPSODY) to G.A.R., P.M., P.F., and M.S. This Joint Undertaking receives support from the European Union's Horizon 2020 research and innovation programme and EFPIA. We also thank the Imperial College London Biomedical Research Centre Genomics Unit and the High Performance Computing Service. Finally, we thank Jorge Ferrer for helpful discussion and Silvia Bonàs-Guarch for interrogating islet eQTL datasets.

AUTHOR CONTRIBUTIONS

M.H. designed and performed all of the molecular and cellular experiments (EMSA, promoter-luciferase assay, CRISPR-Cas9 editing, ChIP-qPCR, and insulin secretion analyses). I.C. provided and analyzed the epigenomic and promoter capture Hi-C data. G.C. performed the western blot analysis. S.J. generated the CRISPR constructs for *STARD10* under the supervision of M.H. A.K., M.C., P.F., A.S., M.S., M.I., and P.M. provided human islet samples and performed the eQTL analysis. F.L.C.-D. and P.J.G. provided the β cell-specific lentiviral vector for CRISPR-Cas9 targeting. L.A.-S. and H.M. provided the human FCHSD2 antibody. S.N. and B.H. performed capacitance measurement. M.H. and G.A.R. conceived the project. G.A.R. supervised all of the studies. M.H., I.C., and G.A.R. wrote the manuscript, with input from all of the authors.

DECLARATION OF INTERESTS

G.A.R. has received grant funding from Les Laboratoires Servier and is a consultant for Sun Pharmaceuticals.

Received: June 6, 2019
Revised: December 10, 2019
Accepted: January 8, 2021
Published: February 2, 2021

REFERENCES

Almeida-Souza, L., Frank, R.A.W., García-Nafría, J., Colussi, A., Gunawardana, N., Johnson, C.M., Yu, M., Howard, G., Andrews, B., Vallis, Y., and McMahon, H.T. (2018). A Flat BAR Protein Promotes Actin Polymerization at the Base of Clathrin-Coated Pits. Cell *174*, 325–337.e14.

Bell, A.C., West, A.G., and Felsenfeld, G. (1999). The protein CTCF is required for the enhancer blocking activity of vertebrate insulators. Cell *98*, 387–396.

Boj, S.F., Parrizas, M., Maestro, M.A., and Ferrer, J. (2001). A transcription factor regulatory circuit in differentiated pancreatic cells. Proc. Natl. Acad. Sci. USA *98*, 14481–14486.

Bonàs-Guarch, S., Guindo-Martínez, M., Miguel-Escalada, I., Grarup, N., Sebastian, D., Rodriguez-Fos, E., Sánchez, F., Planas-Fèlix, M., Cortes-Sánchez, P., González, S., et al. (2018). Re-analysis of public genetic data reveals a rare X-chromosomal variant associated with type 2 diabetes. Nat. Commun. *9*, 321.

Bonev, B., and Cavalli, G. (2016). Organization and function of the 3D genome. Nat. Rev. Genet. *17*, 661–678.

Cao, H., Yin, X., Cao, Y., Jin, Y., Wang, S., Kong, Y., Chen, Y., Gao, J., Heller, S., and Xu, Z. (2013). FCHSD1 and FCHSD2 are expressed in hair cell stereocilia and cuticular plate and regulate actin polymerization in vitro. PLoS ONE *8*, e56516.

Carrat, G.R., Hu, M., Nguyen-Tu, M.-S., Chabosseau, P., Gaulton, K.J., van de Bunt, M., Siddiq, A., Falchi, M., Thurner, M., Canouil, M., et al. (2017). Decreased STARD10 Expression Is Associated with Defective Insulin Secretion in Humans and Mice. Am. J. Hum. Genet. *100*, 238–256.

Carrat, G.R., Haythorne, E., Tomas, A., Haataja, L., Müller, A., Arvan, P., Piunti, A., Cheng, K., Huang, M., Pullen, T.J., et al. (2020). The type 2 diabetes gene product STARD10 is a phosphoinositide-binding protein that controls insulin secretory granule biogenesis. Mol. Metab. *40*, 101015.

de Wit, E., Vos, E.S.M., Holwerda, S.J.B., Valdes-Quezada, C., Verstegen, M.J.A.M., Teunissen, H., Splinter, E., Wijchers, P.J., Krijger, P.H.L., and de Laat, W. (2015). CTCF Binding Polarity Determines Chromatin Looping. Mol. Cell *60*, 676–684.

Doench, J.G., Fusi, N., Sullender, M., Hegde, M., Vaimberg, E.W., Donovan, K.F., Smith, I., Tothova, Z., Wilen, C., Orchard, R., et al. (2016). Optimized sgRNA design to maximize activity and minimize off-target effects of CRISPR-Cas9. Nat. Biotechnol. *34*, 184–191.

Fadista, J., Vikman, P., Laakso, E.O., Mollet, I.G., Esguerra, J.L., Taneera, J., Storm, P., Osmark, P., Ladenvall, C., Prasad, R.B., et al. (2014). Global genomic and transcriptomic analysis of human pancreatic islets reveals novel genes influencing glucose metabolism. Proc. Natl. Acad. Sci. USA *111*, 13924–13929.

Fan, F., Ji, C., Wu, Y., Ferguson, S.M., Tamarina, N., Philipson, L.H., and Lou, X. (2015). Dynamin 2 regulates biphasic insulin secretion and plasma glucose homeostasis. J. Clin. Invest. *125*, 4026–4041.

Fuchsberger, C., Flannick, J., Teslovich, T.M., Mahajan, A., Agarwala, V., Gaulton, K.J., Ma, C., Fontanillas, P., Moutsianas, L., McCarthy, D.J., et al. (2016). The genetic architecture of type 2 diabetes. Nature *536*, 41–47.

Gaulton, K.J., Nammo, T., Pasquali, L., Simon, J.M., Giresi, P.G., Fogarty, M.P., Panhuis, T.M., Mieczkowski, P., Secchi, A., Bosco, D., et al. (2010). A map of open chromatin in human pancreatic islets. Nat. Genet. *42*, 255–259.

Gaulton, K.J., Ferreira, T., Lee, Y., Raimondo, A., Mägi, R., Reschen, M.E., Mahajan, A., Locke, A., Rayner, N.W., Robertson, N., et al.; DIAbetes Genetics Replication And Meta-analysis (DIAGRAM) Consortium (2015). Genetic fine mapping and genomic annotation defines causal mechanisms at type 2 diabetes susceptibility loci. Nat. Genet. *47*, 1415–1425.

Göndör, A., Rougier, C., and Ohlsson, R. (2008). High-resolution circular chromosome conformation capture assay. Nat. Protoc. *3*, 303–313.

Gosselin, D., Link, V.M., Romanoski, C.E., Fonseca, G.J., Eichenfield, D.Z., Spann, N.J., Stender, J.D., Chun, H.B., Garner, H., Geissmann, F., and Glass, C.K. (2014). Environment drives selection and function of enhancers controlling tissue-specific macrophage identities. Cell *159*, 1327–1340.

Graham, F.L., and van der Eb, A.J. (1973). A new technique for the assay of infectivity of human adenovirus 5 DNA. Virology *52*, 456–467.

Guo, Y., Xu, Q., Canzio, D., Shou, J., Li, J., Gorkin, D.U., Jung, I., Wu, H., Zhai, Y., Tang, Y., et al. (2015). CRISPR Inversion of CTCF Sites Alters Genome Topology and Enhancer/Promoter Function. Cell *162*, 900–910.

Hagège, H., Klous, P., Braem, C., Splinter, E., Dekker, J., Cathala, G., de Laat, W., and Forné, T. (2007). Quantitative analysis of chromosome conformation capture assays (3C-qPCR). Nat. Protoc. *2*, 1722–1733.

Hastoy, B., Godazgar, M., Clark, A., Nylander, V., Spiliotis, I., van de Bunt, M., Chibalina, M.V., Barrett, A., Burrows, C., Tarasov, A.I., et al. (2018). Electrophysiological properties of human beta-cell lines EndoC-βH1 and -βH2 conform with human beta-cells. Sci. Rep. *8*, 16994.

Huang, J., Liu, X., Li, D., Shao, Z., Cao, H., Zhang, Y., Trompouki, E., Bowman, T.V., Zon, L.I., Yuan, G.-C., et al. (2016). Dynamic Control of Enhancer Repertoires Drives Lineage and Stage-Specific Transcription during Hematopoiesis. Dev. Cell *36*, 9–23.

Jewell, J.L., Luo, W., Oh, E., Wang, Z., and Thurmond, D.C. (2008). Filamentous actin regulates insulin exocytosis through direct interaction with Syntaxin 4. J. Biol. Chem. *283*, 10716–10726.

Jones, B., Bloom, S.R., Buenaventura, T., Tomas, A., and Rutter, G.A. (2018). Control of insulin secretion by GLP-1. Peptides *100*, 75–84.

Kalwat, M.A., and Thurmond, D.C. (2013). Signaling mechanisms of glucose-induced F-actin remodeling in pancreatic islet β cells. Exp. Mol. Med. *45*, e37.

Khamis, A., Canouil, M., Siddiq, A., Crouch, H., Falchi, M., Bulow, M.V., Ehehalt, F., Marselli, L., Distler, M., Richter, D., et al. (2019). Laser capture microdissection of human pancreatic islets reveals novel eQTLs associated with type 2 diabetes. Mol. Metab. *24*, 98–107.

Khan, A., Fornes, O., Stigliani, A., Gheorghe, M., Castro-Mondragon, J.A., van der Lee, R., Bessy, A., Chèneby, J., Kulkarni, S.R., Tan, G., et al. (2018). JASPAR 2018: update of the open-access database of transcription factor binding profiles and its web framework. Nucleic Acids Res. *46* (*D1*), D1284.

Lupiáñez, D.G., Kraft, K., Heinrich, V., Krawitz, P., Brancati, F., Klopocki, E., Horn, D., Kayserili, H., Opitz, J.M., Laxova, R., et al. (2015). Disruptions of topological chromatin domains cause pathogenic rewiring of gene-enhancer interactions. Cell *161*, 1012–1025.

Mahajan, A., Go, M.J., Zhang, W., Below, J.E., Gaulton, K.J., Ferreira, T., Horikoshi, M., Johnson, A.D., Ng, M.C., Prokopenko, I., et al.; DIAbetes Genetics Replication And Meta-analysis (DIAGRAM) Consortium; Asian Genetic Epidemiology Network Type 2 Diabetes (AGEN-T2D) Consortium; South Asian Type 2 Diabetes (SAT2D) Consortium; Mexican American Type 2 Diabetes (MAT2D) Consortium; Type 2 Diabetes Genetic Exploration by Nex-generation sequencing in muylti-Ethnic Samples (T2D-GENES) Consortium (2014). Genome-wide trans-ancestry meta-analysis provides insight into the genetic architecture of type 2 diabetes susceptibility. Nat. Genet. *46*, 234–244.

Mahajan, A., Taliun, D., Thurner, M., Robertson, N.R., Torres, J.M., Rayner, N.W., Payne, A.J., Steinthorsdottir, V., Scott, R.A., Grarup, N., et al. (2018). Fine-mapping type 2 diabetes loci to single-variant resolution using high-density imputation and islet-specific epigenome maps. Nat. Genet. *50*, 1505–1513.

Mandegar, M.A., Huebsch, N., Frolov, E.B., Shin, E., Truong, A., Olvera, M.P., Chan, A.H., Miyaoka, Y., Holmes, K., Spencer, C.I., et al. (2016). CRISPR Interference Efficiently Induces Specific and Reversible Gene Silencing in Human iPSCs. Cell Stem Cell *18*, 541–553.

Marchetti, P., Suleiman, M., and Marselli, L. (2018). Organ donor pancreases for the study of human islet cell histology and pathophysiology: a precious and valuable resource. Diabetologia *61*, 770–774.

Merkenschlager, M., and Odom, D.T. (2013). CTCF and cohesin: linking gene regulatory elements with their targets. Cell *152*, 1285–1297.

Miguel-Escalada, I., Bonàs-Guarch, S., Cebola, I., Ponsa-Cobas, J., Mendieta-Esteban, J., Atla, G., Javierre, B.M., Rolando, D.M.Y., Farabella, I., Morgan, C.C., et al. (2019). Human pancreatic islet three-dimensional chromatin architecture provides insights into the genetics of type 2 diabetes. Nat. Genet. *51*, 1137–1148.

Morán, I., Akerman, I., van de Bunt, M., Xie, R., Benazra, M., Nammo, T., Arnes, L., Nakić, N., García-Hurtado, J., Rodríguez-Seguí, S., et al. (2012). Human β cell transcriptome analysis uncovers lncRNAs that are tissue-specific, dynamically regulated, and abnormally expressed in type 2 diabetes. Cell Metab. *16*, 435–448.

Morris, A.P., Voight, B.F., Teslovich, T.M., Ferreira, T., Segrè, A.V., Steinthorsdottir, V., Strawbridge, R.J., Khan, H., Grallert, H., Mahajan, A., et al.; Wellcome Trust Case Control Consortium; Meta-Analyses of Glucose and Insulin-related traits Consortium (MAGIC) Investigators; Genetic Investigation of ANthropometric Traits (GIANT) Consortium; Asian Genetic Epidemiology Network–Type 2 Diabetes (AGEN-T2D) Consortium; South Asian Type 2 Diabetes (SAT2D) Consortium; DIAbetes Genetics Replication And Meta-analysis (DIAGRAM) Consortium (2012). Large-scale association analysis provides insights into the genetic architecture and pathophysiology of type 2 diabetes. Nat. Genet. *44*, 981–990.

Nair, G.G., Liu, J.S., Russ, H.A., Tran, S., Saxton, M.S., Chen, R., Juang, C., Li, M.-L., Nguyen, V.Q., Giacometti, S., et al. (2019). Recapitulating endocrine cell clustering in culture promotes maturation of human stem-cell-derived β cells. Nat. Cell Biol. *21*, 263–274.

Ongen, H., Buil, A., Brown, A.A., Dermitzakis, E.T., and Delaneau, O. (2016). Fast and efficient QTL mapper for thousands of molecular phenotypes. Bioinformatics *32*, 1479–1485.

Oudelaar, A.M., Davies, J.O.J., Hanssen, L.L.P., Telenius, J.M., Schwessinger, R., Liu, Y., Brown, J.M., Downes, D.J., Chiariello, A.M., Bianco, S., et al. (2018). Single-allele chromatin interactions identify regulatory hubs in dynamic compartmentalized domains. Nat. Genet. *50*, 1744–1751.

Pagliuca, F.W., Millman, J.R., Gürtler, M., Segel, M., Van Dervort, A., Ryu, J.H., Peterson, Q.P., Greiner, D., and Melton, D.A. (2014). Generation of functional human pancreatic β cells in vitro. Cell *159*, 428–439.

Parker, S.C.J., Stitzel, M.L., Taylor, D.L., Orozco, J.M., Erdos, M.R., Akiyama, J.A., van Bueren, K.L., Chines, P.S., Narisu, N., Black, B.L., et al.; NISC Comparative Sequencing Program; National Institutes of Health Intramural Sequencing Center Comparative Sequencing Program Authors; NISC Comparative Sequencing Program Authors (2013). Chromatin stretch enhancer states drive cell-specific gene regulation and harbor human disease risk variants. Proc. Natl. Acad. Sci. USA *110*, 17921–17926.

Pasquali, L., Gaulton, K.J., Rodríguez-Seguí, S.A., Mularoni, L., Miguel-Escalada, I., Akerman, İ., Tena, J.J., Morán, I., Gómez-Marín, C., van de Bunt, M., et al. (2014). Pancreatic islet enhancer clusters enriched in type 2 diabetes risk-associated variants. Nat. Genet. *46*, 136–143.

Pickrell, J.K. (2014). Joint analysis of functional genomic data and genome-wide association studies of 18 human traits. Am. J. Hum. Genet. *94*, 559–573.

Purcell, S., Neale, B., Todd-Brown, K., Thomas, L., Ferreira, M.A.R., Bender, D., Maller, J., Sklar, P., de Bakker, P.I.W., Daly, M.J., and Sham, P.C. (2007). PLINK: a tool set for whole-genome association and population-based linkage analyses. Am. J. Hum. Genet. *81*, 559–575.

Ravassard, P., Hazhouz, Y., Pechberty, S., Bricout-Neveu, E., Armanet, M., Czernichow, P., and Scharfmann, R. (2011). A genetically engineered human pancreatic β cell line exhibiting glucose-inducible insulin secretion. J. Clin. Invest. *121*, 3589–3597.

Rezania, A., Bruin, J.E., Arora, P., Rubin, A., Batushansky, I., Asadi, A., O'Dwyer, S., Quiskamp, N., Mojibian, M., Albrecht, T., et al. (2014). Reversal of diabetes with insulin-producing cells derived in vitro from human pluripotent stem cells. Nat. Biotechnol. *32*, 1121–1133.

Rorsman, P., and Renström, E. (2003). Insulin granule dynamics in pancreatic beta cells. Diabetologia *46*, 1029–1045.

Scott, R.A., Scott, L.J., Mägi, R., Marullo, L., Gaulton, K.J., Kaakinen, M., Pervjakova, N., Pers, T.H., Johnson, A.D., Eicher, J.D., et al.; DIAbetes Genetics Replication And Meta-analysis (DIAGRAM) Consortium (2017). An Expanded Genome-Wide Association Study of Type 2 Diabetes in Europeans. Diabetes *66*, 2888–2902.

Solimena, M., Schulte, A.M., Marselli, L., Ehehalt, F., Richter, D., Kleeberg, M., Mziaut, H., Knoch, K.-P., Parnis, J., Bugliani, M., et al. (2018). Systems biology of the IMIDIA biobank from organ donors and pancreatectomised patients defines a novel transcriptomic signature of islets from individuals with type 2 diabetes. Diabetologia *61*, 641–657.

Spracklen, C.N., Horikoshi, M., Kim, Y.J., Lin, K., Bragg, F., Moon, S., Suzuki, K., Tam, C.H.T., Tabara, Y., Kwak, S.-H., et al. (2020). Identification of type 2 diabetes loci in 433,540 East Asian individuals. Nature *582*, 240–245.

Stewart, M., Terry, A., Hu, M., O'Hara, M., Blyth, K., Baxter, E., Cameron, E., Onions, D.E., and Neil, J.C. (1997). Proviral insertions induce the expression

CellPress
OPEN ACCESS

of bone-specific isoforms of PEBP2alphaA (CBFA1): evidence for a new myc collaborating oncogene. Proc. Natl. Acad. Sci. USA *94*, 8646–8651.

Varshney, A., Scott, L.J., Welch, R.P., Erdos, M.R., Chines, P.S., Narisu, N., Albanus, R.D., Orchard, P., Wolford, B.N., Kursawe, R., et al.; NISC Comparative Sequencing Program (2017). Genetic regulatory signatures underlying islet gene expression and type 2 diabetes. Proc. Natl. Acad. Sci. USA *114*, 2301–2306.

Vernimmen, D., and Bickmore, W.A. (2015). The Hierarchy of Transcriptional Activation: From Enhancer to Promoter. Trends Genet. *31*, 696–708.

Viñuela, A., Varshney, A., van de Bunt, M., Prasad, R.B., Asplund, O., Bennett, A., Boehnke, M., Brown, A.A., Erdos, M.R., Fadista, J., et al. (2020). Genetic variant effects on gene expression in human pancreatic islets and their implications for T2D. Nat. Commun. *11*, 4912.

Voight, B.F., Scott, L.J., Steinthorsdottir, V., Morris, A.P., Dina, C., Welch, R.P., Zeggini, E., Huth, C., Aulchenko, Y.S., Thorleifsson, G., et al.; MAGIC investigators; GIANT Consortium (2010). Twelve type 2 diabetes susceptibility loci identified through large-scale association analysis. Nat. Genet. *42*, 579–589.

Whyte, W.A., Orlando, D.A., Hnisz, D., Abraham, B.J., Lin, C.Y., Kagey, M.H., Rahl, P.B., Lee, T.I., and Young, R.A. (2013). Master transcription factors and mediator establish super-enhancers at key cell identity genes. Cell *153*, 307–319.

Williams, A., and Flavell, R.A. (2008). The role of CTCF in regulating nuclear organization. J. Exp. Med. *205*, 747–750.

Xiao, G.-Y., Mohanakrishnan, A., and Schmid, S.L. (2018). Role for ERK1/2-dependent activation of FCHSD2 in cancer cell-selective regulation of clathrin-mediated endocytosis. Proc. Natl. Acad. Sci. USA *115*, E9570–E9579.

STAR★METHODS

KEY RESOURCES TABLE

REAGENT or RESOURCE	SOURCE	IDENTIFIER
Antibodies		
CTCF	EMD Millipore	Cat# 07-729; RRID: AB_441965
Rabbit IgG, plasma	EMD Millipore	Cat# 401590; RRID: N/A
STARD10	Santa Cruz Biotechnology	Cat# sc-54336; RRID: AB_2197780
FCHSD2	Almeida-Souza et al., 2018	N/A
ARAP1	Abcam	Cat# ab99382; RRID: AB_10675661
α-TUBULIN	Sigma-Aldrich	Cat# T5168; RRID: AB_477579)
Bacteria and virus strain		
Stbl3 competent cells	Thermo Fisher Scientific	Cat# C737303
10-beta competent cells	New England Biolabs	Cat# C30191
Biological samples		
Human islet samples	Carrat et al., 2017	N/A
Chemicals, peptides, and recombinant proteins		
3-Isobutyl-1-methylxanthine (IBMX)	Sigma-Aldrich	Cat# I5879
Foskolin	Sigma-Aldrich	Cat# F3917
Sodium Selenite	Sigma-Aldrich	Cat# S1382
Nicotinamide	Sigma-Aldrich	Cat# 481907
Paraformaldehyde	Agar Scientific	Cat# AGR1026
TRIzol	Invitrogen	Cat# 15596018
NP40	Sigma-Aldrich	Cat# 13021
Proteinase inhibitor cocktail	Sigma-Aldrich	Cat# 04693132001
Albumin from Bovine Serum Fraction V	Roche Diagnostics	Cat# 10775835001
Human Transferrin	Sigma-Aldrich	Cat# T8158
ECM	Sigma-Aldrich	Cat# E1270
RNase A	Thermo Fisher Scientific	Cat# EN0531
Proteinase K	Sigma-Aldrich	Cat# AM2546
Dynabeads-Protein G	Thermo Fisher Scientific	Cat# 10003D
Dynabeads-Protein A	Thermo Fisher Scientific	Cat# 10008D
ATP, [γ-^{32}P]	Perkin Elmer	Cat# NEG002A
Critical commercial assay		
Dual-Luciferase Assay Reporter Assay System	Promega	Cat# E1910
Lipofectamine 2000	Thermos Fisher Scientific	Cat# 11668025
Insulin Ultra-sensitive Assay Kit	Cisbio Bioassays	Cat# 62IN2PEH
High-Capacity cDNA Reverse Transcription Kit	Thermos Fisher Scientific	Cat# 4368814
NE-PER^{TM} Nuclear and Cytoplasmic extraction reagents	Thermos Fisher Scientific	Cat# 78833
Taqman^{TM} Fast advanced master mix	Thermos Fisher Scientific	Cat# 4444553
Fast SYBR^{TM} Green Master Mix	Thermos Fisher Scientific	Cat# 4385612
Phusion High fidelity DNA polymerase	Thermos Fisher Scientific	Cat# F530
T4 polynucleotide kinase	New England BioLabs	Cat# M0201S
Experimental model: cell line		
HEK293T	ATCC	Cat# CRL_3216; RRID: CVCL_0063
EndoC-βH1	Univercell-Biosolutions	Cat# N/A; RRID: CVCL_L909

(*Continued on next page*)

Continued

REAGENT or RESOURCE	SOURCE	IDENTIFIER
Oligonucleotides		
VR_KO1: GACCCCTGTGAGCTCCTCGT	This paper	N/A
VR_KO2: AGCGACCACCAGCTAGGTTT	This paper	N/A
CBS1: GCTTGGGTGGGGGTGCAGCC	This paper	N/A
CBS2: GGGCAGTCAAGGGCACAGGA	This paper	N/A
CBS5: TGCAGAAGAATGGTCACTAG	This paper	N/A
CBS7: AGTCTCGGGATCGACACGTG	This paper	N/A
CBS8: CGGCCACAACCACTAGGGGG	This paper	N/A
R2_KO1: GTGTCCTTCACAGTGGCGAT	This paper	N/A
R2_KO2: ACTCCACCTTGGTATTACAC	This paper	N/A
STARD10-1: AGAGGCCGCCAGCTTCTCCA	This paper	N/A
STARD10-2: AGTCTTGGTCATCGGGCACC	This paper	N/A
FCHSD2-1: GCATCATGCAGCCGCCGCCG	This paper	N/A
FCHSD2-2: GCCCTTACCTTCCTCGGCGG	This paper	N/A
ARAP1-1: TGGGGATGCTGCGCTATCGG	This paper	N/A
ARAP1-2: TGCACCTGGAGCAGTACACG	This paper	N/A
HBB_KO1: GCACCATAAGGGACATGATA	This paper	N/A
HBB_KO2: TTTCCTTACTAAACCGACAT	This paper	N/A
Inter_KO1: TTGAGGTTACATCATTCTAC	This paper	N/A
Inter_KO2: AGTACCTAAAGAGACCCACA	This paper	N/A
Scrabmble_1: GAACTCAACCAGAGGGCCAA	This paper	N/A
Scrabmble_2: GGGAGGTGGCTTTAGGTTTT	This paper	N/A
Recombinant DNA		
pMD2.G	Didier Trono (https://www.epfl.ch/labs/tronolab/)	Cat# 12259; RRID: Addgene_12259
pSPAX2	Didier Trono (https://www.epfl.ch/labs/tronolab/)	Cat# 12260; RRID: Addgene_12260
BAC DNA	bacpacresources.org	RP11-101P7
pLenti-CRISPR-RIP-Cas9	Paul Gadue	N/A
pGL3-promoter	Promega	E1761
pRL-Renilla	Promega	E2231
pBlueScript II KS+	Stratagene/Agilent	212207
Software		
GraphPad Prism 7	GraphPad Software	RRID: SCR_002798
Illustrator	Adobe	RRID:SCR_010279
Photoshop	Adobe	RRID: SCR_014199

RESOURCE AVAILABILITY

Lead contact

Further information and requests for resources and regents should be directed to and will be fulfilled by the lead Contact, Guy A Rutter (g.rutter@imperial.ac.uk)

Materials availability

All unique/stable reagents generated in this study are available from the Lead Contact without restriction

Data and code availability

This study did not generate any unique datasets or code.

EXPERIMENTAL MODEL AND SUBJECT DETAILS

The human-derived β cell line EndoC-βH1 was grown on ECM (1% v/v) and Fibronectin (2 μg/ml)-coated plates or Petri dishes in serum-free DMEM containing low glucose (1 g/L), 2% (w/v) albumin from bovine serum fraction V, 50 μM β-Mercaptoethanol,

10 mM nicotinamide, 5.5 μg/mL human transferrin, 6.7 ng/mL sodium selenite, penicillin (100 units/mL), and streptomycin (100 μg/mL) (Ravassard et al., 2011). HEK293T cell was cultured in DMEM high glucose medium supplemented with 10% fetal bovine serum, 6 mM L-glutamine, penicillin (100 μg/mL) and streptomycin (100 μg/mL).

METHOD DETAILS

Electrophoretic mobility shift assay (EMSA)

Electrophoretic mobility shift assay (EMSA) was carried out as previously described (Stewart et al., 1997). In brief, complementary oligonucleotides were designed to contain either risk or protective variants (Table S3). EndoC-βH1 nuclear extract was prepared using NE-PER nuclear and cytoplasmic extraction kit according to manufacturer's instruction (Thermo Scientific). Oligonucleotides bearing either risk or protective variants were synthesized (Sigma) and end-labeled with γ-^{32}P-ATP using T4 polynucleotide kinase (New England BioLabs). ^{32}P-labeled oligoes were incubated for 20 min at room temperature with 5 μg of nuclear extract in binding reactions consisted of 1 x binding buffer (20 mM HEPES pH 7.9, 90 mM KCl, 5 mM $MgCl_2$, and 0.05% Nonidet P-40) and 1 μg poly(dI-dC). Samples were electrophoresed on a 5% acrylamide gel in 0.5 x TBE buffer (90 mM Tris, 64.6 mM Boric acid and 2.5 mM EDTA, pH 8.3). The acrylamide gel was then vacuum-dried and autoradiographed.

Chromatin conformation capture (3C and 4C)

3C was performed as described (Hagège et al., 2007). In brief, a suspension of 1 × 10^7 EndoC-βH1 cells was cross-linked with 4% (v/v) formaldehyde at room temperature for 10 min. The cross-linked DNA was digested overnight with restriction enzyme *Nco*I and then ligated with T4 DNA ligase at 16°C overnight. The ligated 3C DNA was purified by extraction with phenol/chloroform and precipitation with ethanol. The ligation products were quantitated by Taqman™ qPCR and normalized to the human *CXCL12* gene. The standard curve for each primer pair was generated using *Nco*I-digested and ligated BAC DNA (RP11-101P7) encompassing the human *ARAP1*, *STARD10*, and *ATG16L2* loci. The Taqman™ probes and primers used for the 3C experiments presented in this study are listed in Table S3. Taqman™ qPCR were carried out on a 7500 Fast Real-Time PCR System (Applied Biosystems). PCR reactions were set as follows: 95°C for 10 min., then with 45 cycles at 95°C 30 s and 58°C 45 s. Crosslinking frequencies were plotted as percentage of that of the human *CXCL12* gene. BamHI-digested and ligated 3C sample was used as a negative control.

For 4C, 1 × 10^7 EndoC-βH1 cells were fixed with 4% paraformaldehyde, digested with restriction enzyme PstI, ligated with T4 DNA ligase and then digested with second restriction enzyme *Msp*I. After the second round of DNA ligation with T4 DNA ligase, DNAs were purified with phenol/chloroform extraction and ethanol precipitation. PCR reactions were carried out to amplify ligation products using nested PCR primer sets (Table S3). PCR products were then digested with restriction enzymes *Xho*I and *Not*I and sub-cloned into pBluescript II KS+ (pBSKS) (Stratagene/Agilent) for Sanger sequencing analysis.

Chromatin immunoprecipitation

Immunoprecipitation was carried out according to a standard protocol (Boj et al., 2001). In brief, 1 × 10^6 EndoC-βH1 cells were fixed with 1% (v/v) formaldehyde for 10 min. and quenched with 1.25 mM glycine. Cells were then scraped and resuspended in lysis buffer (2% Triton-100, 1% SDS, 100 mM NaCl, 10 mM Tris-HCl, 1 mM EDTA). After 20 stokes of homogenization with a disposable pestle, cells were sonicated for 10 min. using Covaris™ S220 to breakdown genomic DNA to 200-500 bp fragments. DNA/protein complexes were then precipitated with anti-CTCF antibody or rabbit IgG (EMD Millipore) conjugated with protein A and G beads. DNAs were purified through Phenol/Chloroform extraction and Ethanol precipitation.

PCR and qPCR

Fusion high fidelity Taq polymerase (Thermo Fisher Scientific) was used in all routine PCR reactions to avoid PCR errors. A typical PCR reaction was set as follow: 98°C for 30 s, then with 35 cycles at 98°C 10 s, 60°C 10 s and 72°C 15 s. The primer sets for genomic DNA amplification are listed in Table S3.

Total RNA from EndoC-βH1 cells was obtained using TRIzol reagent (Invitrogen). Total RNAs (2 μg) were then reverse-transcribed into first strand cDNAs using High-Capacity cDNA Reverse Transcription Kit (Thermo Fisher Scientific) according to the manufacturer's instructions. Real-time PCR was performed on a 7500 Fast Real-Time PCR System using the Fast SYBR™ Green master mix or Fast Taqman™ master mix. The SYBR™ Green PCR primer sets for variant region and CTCF binding site (CBS) are listed in Table S2. The experiment was performed in duplicate and repeated three times.

Molecular cloning

Active enhancer regions, identified by integration of previously published human islet ATAC-seq and H3K27ac ChIP-seq datasets (Miguel-Escalada et al., 2019), were PCR-amplified from BAC DNA (RP11-101P7) with primer sets (Table S3) designed by Primer3-based software and cloned into pGL3-promoter vector between NheI and XhoI restriction enzyme sites. Plasmid DNA was extracted using mini-prep plasmid extraction kit and/or Maxi-prep plasmid extraction kit (QIAGEN). Correct cloning was confirmed by Sanger sequencing.

Transfection and luciferase assay

EndoC-βH1 cells were seeded at a density of 50,000 per well in 48-well plates. After 48 hours, 0.4 μg of luciferase constructs containing putative regulatory sequences were co-transfected with 1 ng of pRL-Renilla construct as internal control into EndoC-βH1 cells, using Lipofectamine 2000, according to manufacturer's instruction. pGL3-promoter vector was served as a negative control. 48 h later, transfected cells were washed once with PBS and lysed directly in passive cell lysis buffer (Promega). Cells were incubated on a rotating platform at room temperature for 10 min. to ensure complete lysis of cells, and then spun at 10,000 rpm for 10 min to remove cell debris. Supernatant was transferred into a fresh tube and used to measure luciferase activity with Dual-Luciferase Reporter Assay kit (Promega) on a Lumat LB9507 luminometer (Berthold Technologies). Firefly luciferase measurements were normalized to *Renilla* luciferase.

CRISPR-Cas9-mediated genome editing

gRNA sequences were designed using the software provided by Broad Institute (https://portals.broadinstitute.org/gpp/public/analysis-tools/sgrna-design) (Doench et al., 2016). To generate mutations or deletions in EndoC-βH1 cells, lentiviral constructs carrying both gRNA and humanized *S. pyogenes* Cas9 (*hsp*Cas9) were transfected into HEK293T cells together with packaging plasmids PMD2.G and psPAX2 using $CaCl_2$ transfection protocol (Graham and van der Eb, 1973). The lentiviral vector containing RIP-Cas9 gene cassette but without gRNA was served as a SHAM control. Next day, cells were treated with sodium butyrate (10 mM) for 8 hours before changing to fresh medium. The medium was collected twice in the next two days and subjected to ultracentrifugation (Optima XPN-100 Ultracentrifuge, Beckman Coulter) at 26,000 rpm for 2 hours at 4°C. The lentiviruses were collected from the bottom of the tube and titrated. Same amount of viruses was used to transduce to EndoC-βH1 cells (MOI = 10). Puromycin (4 μg/ml) was added 72 h after infection to select lentivirus-infected cells. For deletion of genomic regions, two plasmids carrying two different gRNAs flanking target regions were co-transfected into HEK293T cells with packaging plasmids. All sequences of gRNAs and primers used for genotyping of genome editing experiments in this study are listed in STAR methods and Table S3, respectively.

To measure deletion efficiency after CRISPR/Cas9 mediated genome editing, Cyber Green qPCR was deployed to detect wild-type allele using primers 1 and 2 from genomic DNA extracted from control and DNA deletedcells. *CXCL12* was served as an internal DNA copy number control (Key resources table). The deletion efficiency was calculated as: $[1\text{-}2^{\Delta\Delta Ct(\text{del-}CXCL12)}/2^{\Delta\Delta Ct(\text{WT-}CXCL12)}]$ x 100%. In addition, the relative values of DNA deletion or inversion was also measured using primer set 1+4 or 1+3 respectively. The primers were listed in Table S3.

Electrophysiology

Membrane capacitance measurements were performed at 32°C in standard whole cell configuration. The recordings were performed using an EPC-10 amplifier and Pulse software. In brief, the extracellular medium was composed of: 118 mM NaCl, 5.6 mM KCl, 2.6 mM $CaCl_2$, 1.2 mM $MgCl_2$, 5 mM HEPES, and 20 mM tetraethylammonium (TEA) (pH 7.4 with NaOH). The intracellular medium comprised: 129 mM CsOH, 125 mM Glutamic acid, 20 mM CsCl, 15 mM NaCl, 1 mM $MgCl_2$, 0.05 mM EGTA, 3 mM ATP, 0.1 mM cAMP, 5 mM HEPES (pH7.2 with CsOH). Exocytosis was detected as changes in cell membrane capacitance while the cells were subjected to ten depolarisations (pulse) of 500ms from −70mV to 0mV at 1Hz. For each cell, the size is determined by the membrane capacitance value measured before the first pulse and the calcium current measured from −40mV to +40mV is triggered by a 100ms depolarisation from the resting potential (−70mV). For each recording, the amplitude of the calcium current corresponds to the average of the second component of the inward current in order to avoid the impact of the sodium current. Data were analyzed using R software with ggstatsplot (https://CRAN.R-project.org/package=ggstatsplot) and plotted in Origin Pro 2020 software. The Analysis of Variance was performed using Welch's ANOVA followed by Games-Howell post hoc test and p values adjusted by the Benjamini & Hochberg procedure.

Insulin secretion

EndoC-βH1 cells were seeded onto ECM/Fibronectin-coated 48-well plates at 2.5 x 10^5 cells per well. Four days after seeding, cells were incubated overnight in a glucose starving medium (glucose-free DMEM supplemented with 2% Albumin from bovine serum fraction V, 50 μL 2-mercaptoethanol, 10 mM nicotinamide, 5.5 μg/ml transferrin, 6.7 ng/ml sodium selenite, 100 units/ml, penicillin, 100 μg/ml streptomycin and 2.8 mM glucose).

The next morning cells were incubated for 1 h in Krebs-Ringer solution [0.2% BSA, 25% solution 1 (460 mM NaCl), 25% solution II (96 mM $NaHCO_3$, 20 mM KCl and 4 mM $MgCl_2$), 25% solution III (4 mM $CaCl_2$), 10 mM HEPES] supplemented with 0.5 mM glucose. EndoC-βH1 cells were then incubated in the presence of low (0.5 mM) or high glucose (15 mM) or other stimuli [0.5 mM IBMX or 20 nM Forskolin or 20 mM KCl]. After incubation for 1 h, the supernatant was collected, placed onto ice and centrifuged for 5 min. at 3,000 rpm at 4°C. The supernatant was then transferred into a fresh tube. Cells were lysed in 50 μL of cell lysis solution (TETG: 20 mM Tris pH 8.0, 1% Triton X-100, 10% glycerol, 137 mM NaCl, 2 mM EGTA). The lysate was then removed to a fresh tube and centrifuged at 3,000 rpm for 5 min at 4°C. Insulin content was measured using an insulin ultra-sensitive assay kit. Secreted insulin was normalized as percentage of total insulin content. Fold increase in glucose- or other stimuli-stimulated insulin secretion is expressed as a ratio in comparison with secretion at basal level (0.5 mM glucose). Insulin secretion assays were performed in either duplicate or triplicate with insulin measurement in duplicates, as indicated.

Human islet regulome and interactome analysis

Human islet regulome maps, including accessible chromatin regions (ATAC-seq) and ChIP-seq for H3K27ac, CTCF and different islet-enriched TFs (NKX2.2, FOXA2, PDX1 and MAFB) were obtained from previously published datasets (Pasquali et al., 2014; Miguel-Escalada et al., 2019) and visualized using the UCSC Genome Browser (http://genome.ucsc.edu/) with GRCh37/hg19 assembly. Represented data corresponds to consolidated tracks released by Miguel-Escalada et al. (2019). Details on number of samples per track are available (Miguel-Escalada et al., 2019). Previously published human islet RNA-seq (Morán et al., 2012), chromatin interaction maps (Miguel-Escalada et al., 2019) were visualized on the WashU Epigenome browser using this session link: http://epigenomegateway.wustl.edu/browser/?genome=hg19&session=62hGf7nfcS&statusId=140947077. For pcHi-C interactions, only interactions with a CHiCAGO score > 5 were taken as high-confidence interactions, as previously described (Miguel-Escalada et al., 2019). For visualization of purposes only, pcHi-C interactions mediated by the CTCF sites R1 and R13 were colored in orange and all other high-confidence interactions in gray (Figure 5A).

Transcription factor binding motif analysis

TF binding profile on genetic variants was carried out using JASPAR CORE program (http://jaspar.genereg.net) (Khan et al., 2018). The threshold of relative profile score was set up at 80%. The scores of potential transcription factors were compared between risk and protective variants and listed in Table S1.

Expression quantitative trait loci (eQTL) analysis

Pancreatic tissues and blood samples were collected from 103 patients that have undergone partial pancreatectomy from the IMIDIA consortium (Khamis et al., 2019; Solimena et al., 2018), with appropriate permissions from donors and/or families. Briefly, expression data was acquired from islets isolated by laser capture microdissection from surgical specimens using Human Genome U133 Plus2.0 Array (Affymetrix). DNA was genotyped using the 2.5 M Omniarray beadchip (Illumina) and imputed with 1000 Genomes reference panel (phase 3), resulting in 7.5 M SNPs. Standard quality control assessment was carried out on the genotyping data using PLINK (Purcell et al., 2007). Expression and genotype analysis was combined to generate eQTLs, performed with FastQTL (Ongen et al., 2016) with gender and age as covariates. A cis-window of 500 kb was used, i.e., the maximum distance at which a gene-SNP is considered local.

QUANTIFICATION AND STATISTICAL ANALYSIS

Data are expressed as means ± SEM. Significance was tested by Student's two-tailed t test, Mann-Whitney test for non-parametric data, and one- or two-way ANOVA with SIDAK multiple comparison test, as appropriate, using Graphpad Prism 7.0 software. $p < 0.05$ was considered significant. The statistical details can be found in Method details and in the figure legends (indicated as *n* number).a

Cell Reports, Volume *34*

Supplemental Information

Chromatin 3D interaction analysis of the *STARD10* locus unveils *FCHSD2* as a regulator of insulin secretion

Ming Hu, Inês Cebola, Gaelle Carrat, Shuying Jiang, Sameena Nawaz, Amna Khamis, Mickaël Canouil, Philippe Froguel, Anke Schulte, Michele Solimena, Mark Ibberson, Piero Marchetti, Fabian L. Cardenas-Diaz, Paul J. Gadue, Benoit Hastoy, Leonardo Alemeida-Souza, Harvey McMahon, and Guy A. Rutter

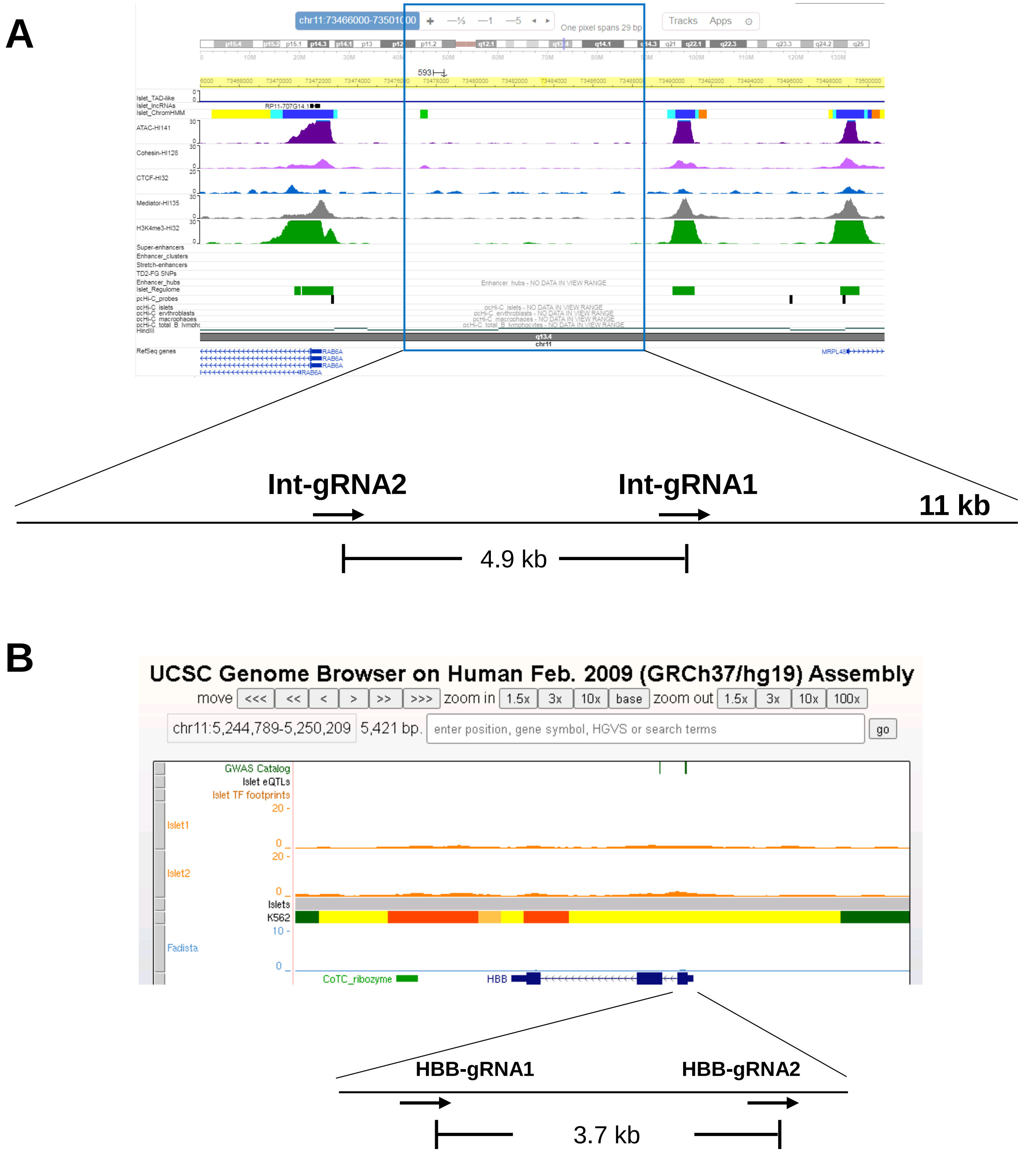

Figure S1. Diagrams of intergenic region and hemoglobin (HBB) gene locus, related to Figure 1D.

(A) Diagram of an intergenic region between *RAB6A* and *MRPL48* genes, 500 Kb downstream of *FCHSD2* gene. The region is epigenetically silent in human islets as demonstrated by the ATAC-seq and epigenomic data. Two gRNAs (gRNA1 and gRNA2) were designed to delete a 4.9 kb genomic DNA fragment.

(B) Diagram of β-globin (*HBB*) gene region. *HBB* gene is not expressed in the human islets. Two gRNAs were designed to delete a 3.7 kb genomic DNA fragment.

Suppl. Fig.1

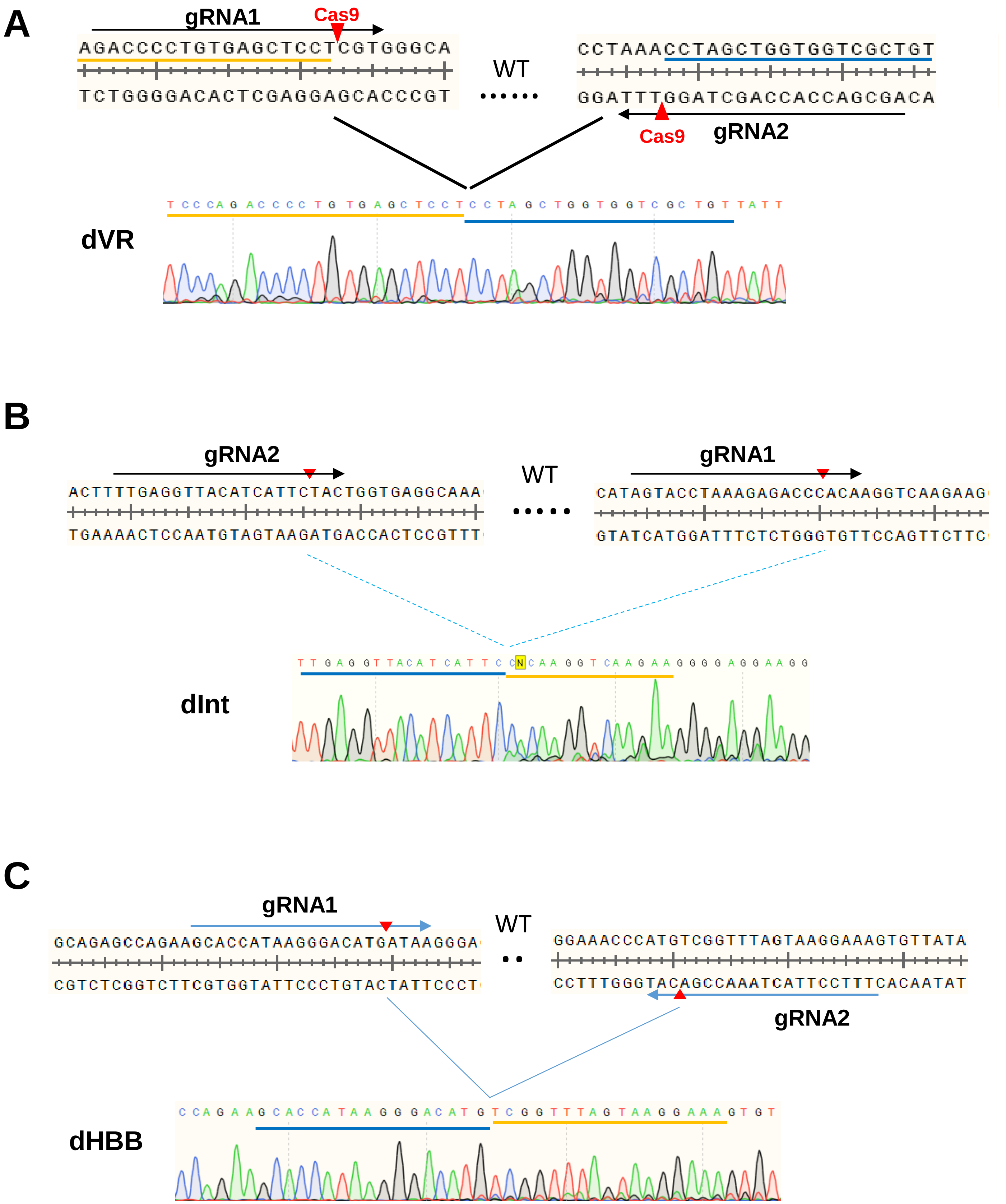
A
gRNA1
Cas9
AGACCCCTGTGAGCTCCTCGTGGGCA
TCTGGGGACACTCGAGGAGCACCCGT
WT
CCTAAACCTAGCTGGTGGTCGCTGT
GGATTTGGATCGACCACCAGCGACA
Cas9
gRNA2
dVR
B
gRNA2
ACTTTTGAGGTTACATCATTCTACTGGTGAGGCAAA
TGAAAACTCCAATGTAGTAAGATGACCACTCCGTTT
WT
gRNA1
CATAGTACCTAAAGAGACCCACAAGGTCAAGAAG
GTATCATGGATTTCTCTGGGTGTTCCAGTTCTTC
dInt
C
gRNA1
GCAGAGCCAGAAGCACCATAAGGGACATGATAAGGGA
CGTCTCGGTCTTCGTGGTATTCCCTGTACTATTCCCT
WT
GGAAACCCATGTCGGTTTAGTAAGGAAAGTGTTATA
CCTTTGGGTACAGCCAAATCATTCCTTTCACAATAT
gRNA2
dHBB

Suppl. Fig.2

Figure S2. Deletion of genomic DNA in EndoC-βH1 cells, related to Figure 1D.

(A) Sanger sequencing of PCR product amplified from wild-type and dVR cell. Orange and blue bars: 5' and 3' end of DNA sequences flanking VR region, respectively.

(B) Sanger sequencing of PCR product amplified from wild-type and dInt cell. Blue and orange bars: 5' and 3' end of DNA sequences flanking intergenic region, respectively.

(C) Sanger sequencing of PCR product amplified from wild-type and dHBB cell. Blue and orange bars: 5' and 3' end of DNA sequences flanking HBB region, respectively.

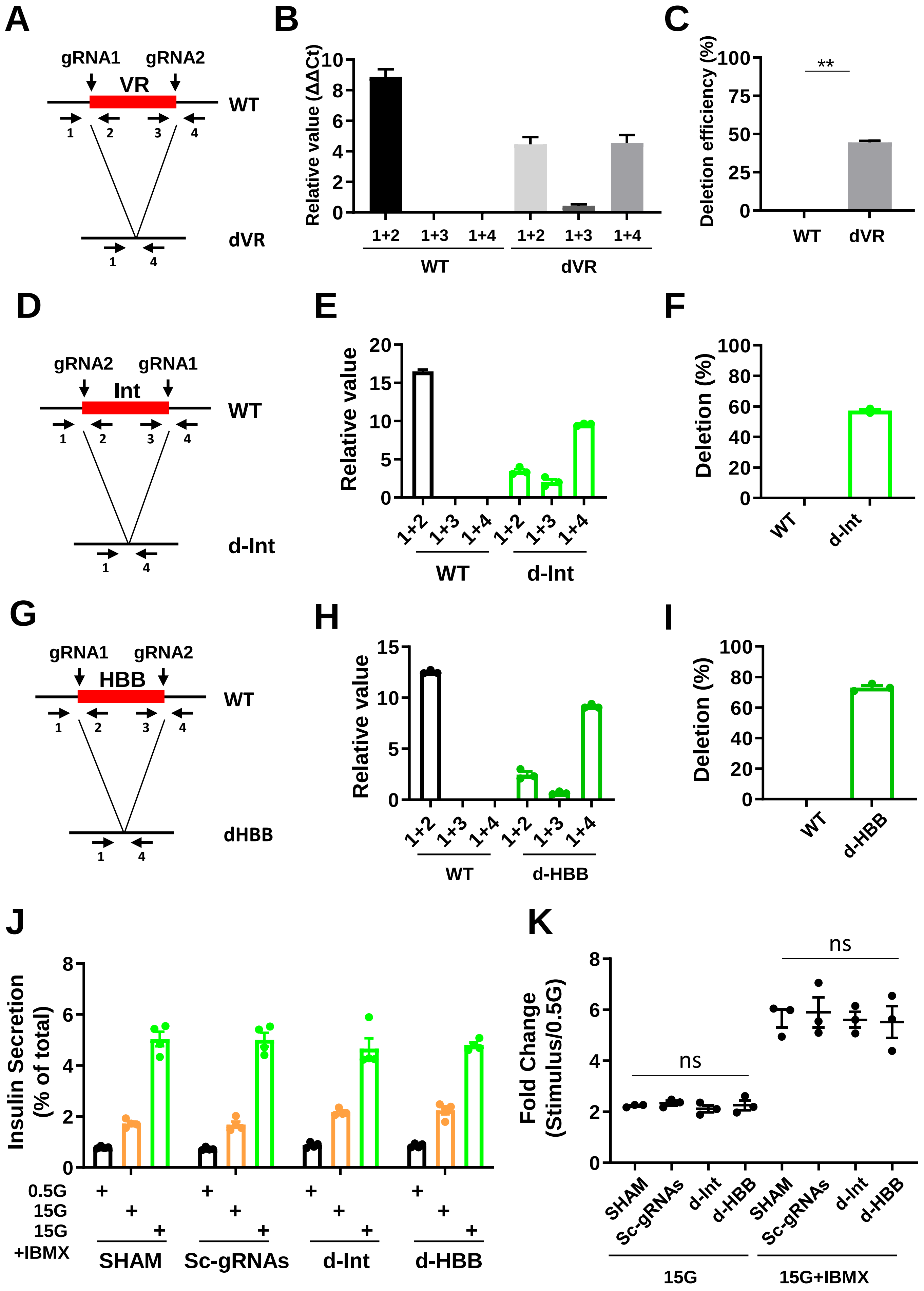

A
gRNA1
gRNA2
VR
WT
1
2
3
4
dVR
1
4
B
Relative value (ΔΔCt)
10
8
6
4
2
0
1+2
1+3
1+4
1+2
1+3
1+4
WT
dVR
C
Deletion efficiency (%)
100
75
50
25
0
**
WT
dVR
D
gRNA2
gRNA1
Int
WT
1
2
3
4
d-Int
1
4
E
Relative value
20
15
10
5
0
1+2
1+3
1+4
1+2
1+3
1+4
WT
d-Int
F
Deletion (%)
100
80
60
40
20
0
WT
d-Int
G
gRNA1
gRNA2
HBB
WT
1
2
3
4
dHBB
1
4
H
Relative value
15
10
5
0
1+2
1+3
1+4
1+2
1+3
1+4
WT
d-HBB
I
Deletion (%)
100
80
60
40
20
0
WT
d-HBB
J
Insulin Secretion (% of total)
8
6
4
2
0
0.5G
15G
15G +IBMX
SHAM
Sc-gRNAs
d-Int
d-HBB
K
Fold Change (Stimulus/0.5G)
8
6
4
2
0
ns
ns
SHAM
Sc-gRNAs
d-Int
d-HBB
SHAM
Sc-gRNAs
d-Int
d-HBB
15G
15G+IBMX

Suppl. Fig.3

Figure S3. Effect of genome deletion in β cell function, related to Figure 1D-1G.

(A) Diagram of SYBR™ Green qPCR analysis at variant region (VR). Primers 1 and 2 were designed to amplify wildtype genomic DNA; primers 1 and 3 detect inversion of DNA after editing and primers 1 and 4 amplify DNA fragment after deletion. The deletion efficiency rate (%) was determined by: 1. calculating the remaining wildtype allele (primer 1+2) in dVR genomic DNA and compared with wildtype allele in SHAM control and 2. taking away the rate of inversion (primer 1+3) in dVR cells. *CXCL12* gene was served as an internal DNA copy number control.

(B) Representative data of SYBR™ Green qPCR analysis on wildtype and dVR genomic DNAs.

(C) Deletion efficiency. Data are mean ± SEM. *, $P < 0.05$; **, $P < 0.01$; ***, $P < 0.005$. $n = 3$.

(D) Diagram of SYBR™ Green qPCR analysis at intergenic region (Int). Primers 1 and 2 were designed to amplify wildtype genomic DNA; primers 1 and 3 detect inversion of DNA after editing and primers 1 and 4 amplify DNA fragment after deletion. *CXCL12* gene was served as an internal DNA copy number control.

(E) Representative data of SYBR™ Green qPCR analysis on wildtype and dInt genomic DNAs.

(F) Deletion efficiency of the intergenic region. Data are mean ± SEM. *, $P < 0.05$; **, $P < 0.01$; ***, $P < 0.005$. $n = 3$.

(G) Diagram of SYBR™ Green qPCR analysis at *HBB* region. Primers 1 and 2 were designed to amplify wildtype genomic DNA; primers 1 and 3 detect inversion of DNA after editing and primers 1 and 4 amplify DNA fragment after deletion. *CXCL12* gene was served as an internal DNA copy number control.

(H) Representative data of SYBR™ Green qPCR analysis on wildtype and dHBB genomic DNAs.

(I) Deletion efficiency of *HBB* gene region. Data are mean ± SEM. *, $P < 0.05$; **, $P < 0.01$; ***, $P < 0.005$. $n = 3$.

(J-K) GSIS assay of CRISPR-Cas9 control cell lines. (J) Representative data of insulin secretion stimulated by 15 mM glucose or 15 mM glucose plus IBMX. (K) Fold change of secreted insulin. Data are normalized to insulin secretion at basal level (0.5 mM glucose) and drawn from 3 independent experiments ($n = 3$). Data are mean ± SEM. *, $P < 0.05$; **, $P < 0.01$; ***, $P < 0.005$.

.

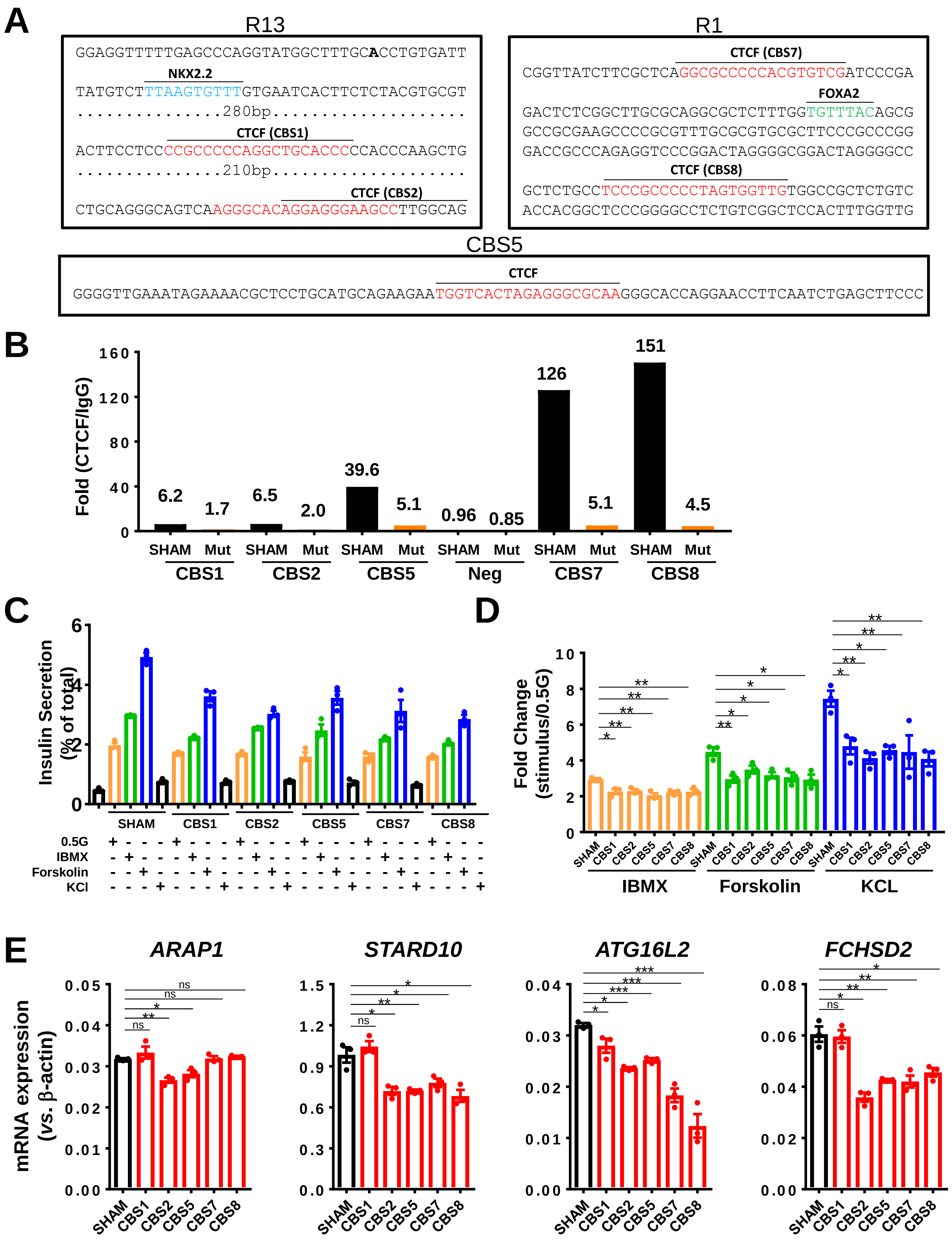
A
R13
GGAGGTTTTTGAGCCCAGGTATGGCTTTGCACCTGTGATT
NKX2.2
TATGTCTTTAAGTGTTTGTGAATCACTTCTCTACGTGCGT
...............280bp...................
CTCF (CBS1)
ACTTCCTCCCCGCCCCCAGGCTGCACCCCCACCCAAGCTG
...............210bp...................
CTCF (CBS2)
CTGCAGGGCAGTCAAGGGCACAGGAGGGAAGCCTTGGCAG
R1
CTCF (CBS7)
CGGTTATCTTCGCTCAGGCGCCCCCACGTGTCGATCCCGA
FOXA2
GACTCTCGGCTTGCGCAGGCGCTCTTTGGTGTTTACAGCG
GCCGCGAAGCCCCGCGTTTGCGCGTGCGCTTCCCGCCCGG
GACCGCCCAGAGGTCCCGGACTAGGGGCGGACTAGGGGCC
CTCF (CBS8)
GCTCTGCCTCCCGCCCCCTAGTGGTTGTGGCCGCTCTGTC
ACCACGGCTCCCGGGGCCTCTGTCGGCTCCACTTTGGTTG
CBS5
CTCF
GGGGTTGAAATAGAAAACGCTCCTGCATGCAGAAGAATGGTCACTAGAGGGCGCAAGGGCACCAGGAACCTTCAATCTGAGCTTCCC
B
Fold (CTCF/IgG)
6.2
1.7
6.5
2.0
39.6
5.1
0.96
0.85
126
5.1
151
4.5
SHAM Mut SHAM Mut SHAM Mut SHAM Mut SHAM Mut SHAM Mut
CBS1 CBS2 CBS5 Neg CBS7 CBS8
C
Insulin Secretion (% of total)
SHAM CBS1 CBS2 CBS5 CBS7 CBS8
0.5G
IBMX
Forskolin
KCl
D
Fold Change (stimulus/0.5G)
SHAM CBS1 CBS2 CBS5 CBS7 CBS8
IBMX
Forskolin
KCL
E
mRNA expression (vs. β-actin)
ARAP1
STARD10
ATG16L2
FCHSD2
SHAM CBS1 CBS2 CBS5 CBS7 CBS8

Suppl. Fig.4

Figure S4. CTCF-binding site (CBS) at *STARD10* locus, related to Figure 3.

(A) DNA sequencing diagrams of ChIP-qPCR identified CBSs at R13 and R1 regions. CBS1 and 2 are within R13, CBS5 is 2.7 kb downstream of R13, and CBS7 and 8 are within R1 region.

(B) Comparison of CTCF binding affinity before and after CRISPR-Cas9 editing at CBSs. CTCF binding affinity was normalized to IgG control in wild-type and CBS mutate cells. The numbers above each bar represent the fold change before and after genome editing. The data are pulled from two independent experiments.

(C) Representative data of Insulin secretion stimulated by multiple stimuli. Cells were treated with 0.5 mM glucose for an hour and then stimulated with either IBMX (0.5 μM) or Forskolin (20 nM) or KCl (20 μM). Data are mean ± SEM. *, $P < 0.05$; **, $P < 0.01$; ***, $P < 0.005$.

(D) Fold change of secreted insulin. Data are normalized to insulin secretion at basal level (0.5 mM glucose) and drawn from 3 independent experiments ($n = 3$). *, $P < 0.05$; **, $P < 0.01$; ***, $P < 0.005$.

(E) Representative data of Taqman^{TM} qRT-PCR analysis in CBS mutate cells. G. *ARAP1*; H. *STARD10*; I. *ATG16L2* and J. *FCHSD2*. *, $P < 0.05$; **, $P < 0.01$; ***, $P < 0.005$. $n = 3$.

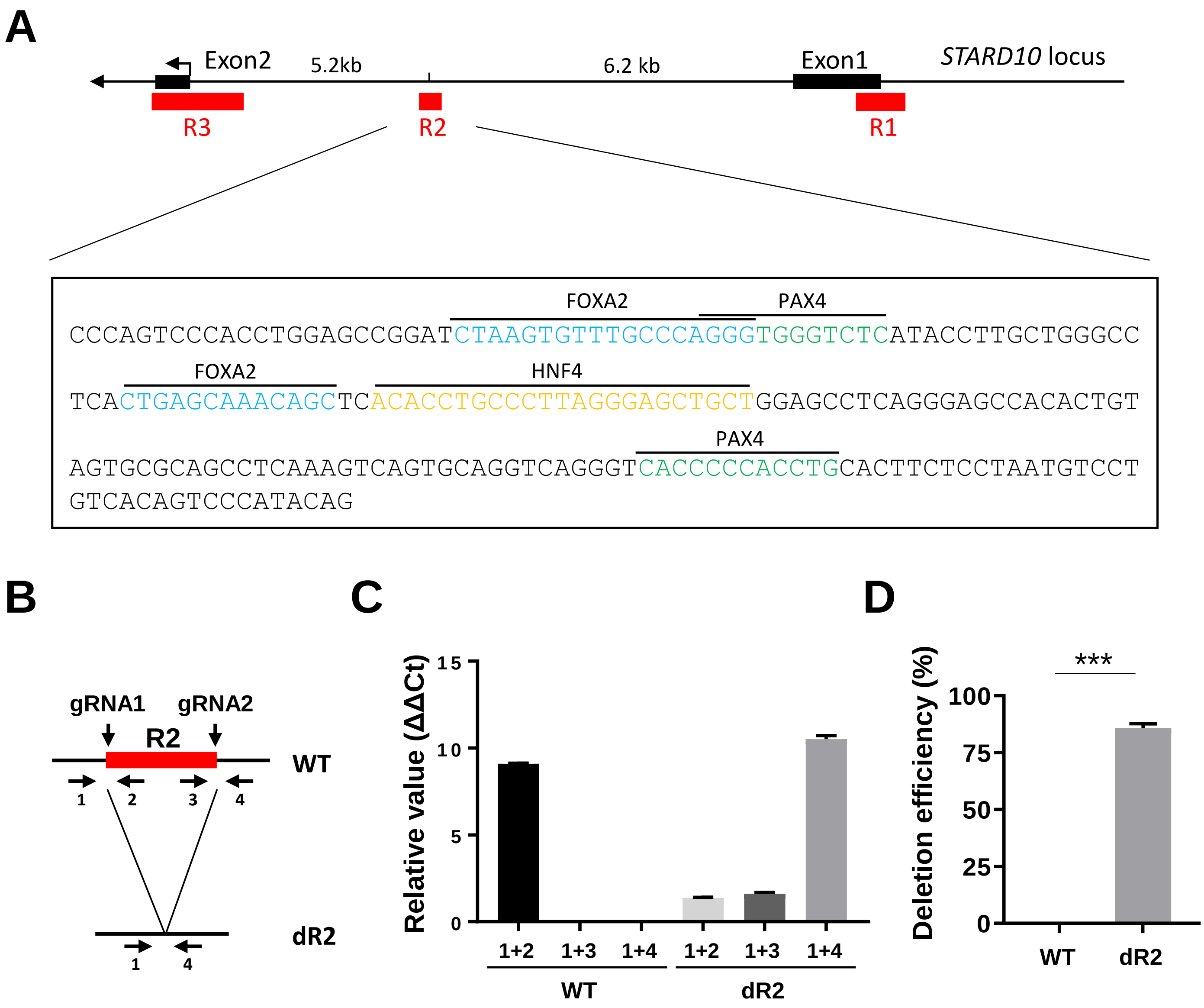

Figure S5. Deletion of R2 enhancer in EndoC-βH1 cells, related to Figure 4.

(A) Diagram of genomic DNA surrounding R2 region. R2 enhancer is located between two promoters of *STARD10* gene (R3 and R1) with multiple binding sites for islet-associated transcription factors.

(B) Diagram of primers designed for SYBR^{TM} Green PCR analysis at R2 region. Primers 1 and 2 were designed to amplify wildtype genomic DNA; primers 1 and 3 were to detect DNA inversion after genome editing and primers 1 and 4 were to amplify DNA fragment after deletion. CXCL12 gene was served as an internal DNA copy number control.

(C) Representative data of SYBR^{TM} Green qPCR analysis in SHAM and dR2 cells.

(D) Deletion efficiency. Data are mean ± SEM. *, $P < 0.05$; **, $P < 0.01$; ***, $P < 0.005$. $n = 2$.

Suppl. Fig.5

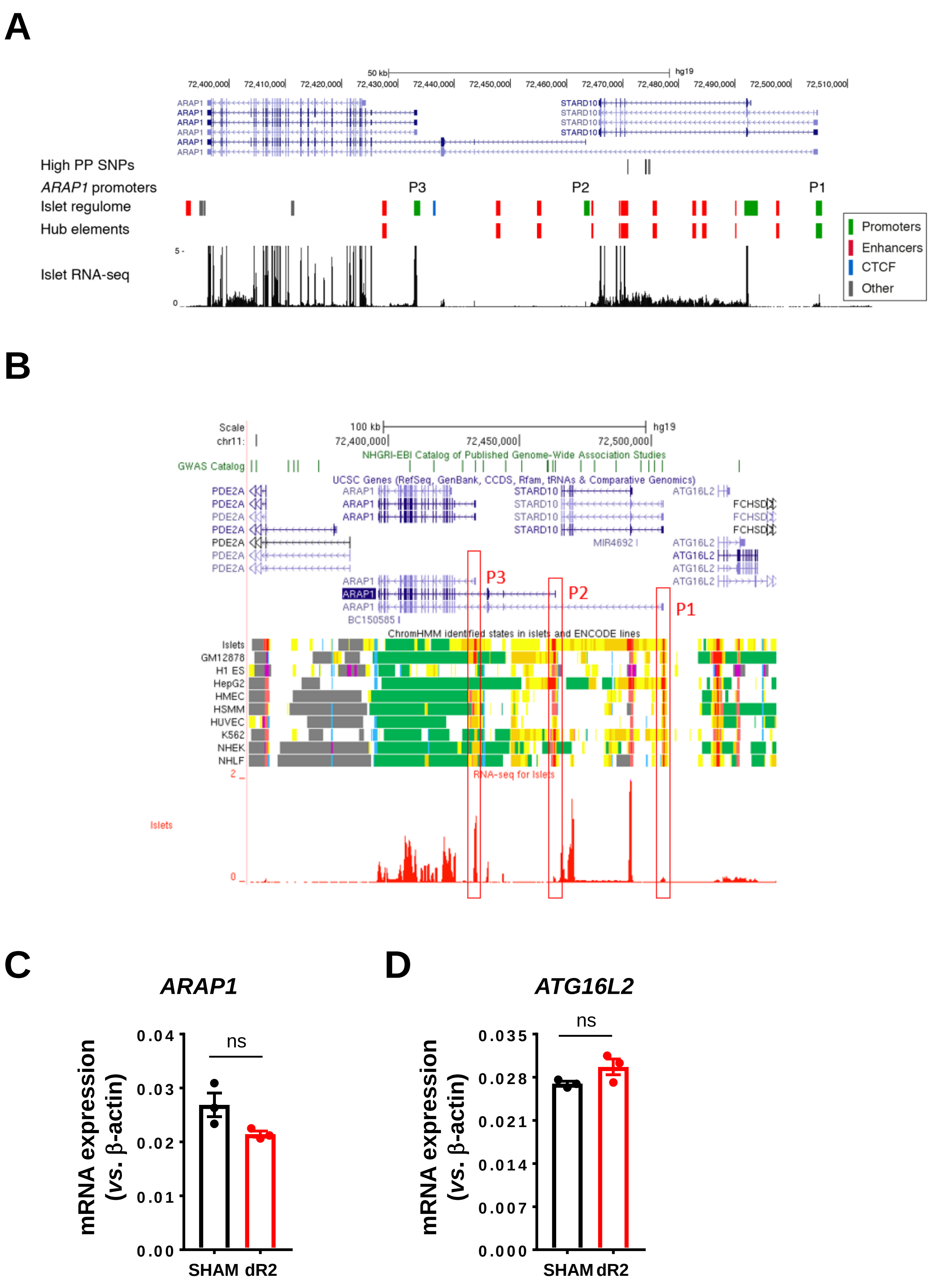
A
50 kb
hg19
72,400,000
72,410,000
72,420,000
72,430,000
72,440,000
72,450,000
72,460,000
72,470,000
72,480,000
72,490,000
72,500,000
72,510,000
ARAP1
STARD10
High PP SNPs
ARAP1 promoters
P3
P2
P1
Islet regulome
Hub elements
Islet RNA-seq
Promoters
Enhancers
CTCF
Other
B
Scale
chr11:
100 kb
hg19
72,400,000
72,450,000
72,500,000
NHGRI-EBI Catalog of Published Genome-Wide Association Studies
GWAS Catalog
UCSC Genes (RefSeq, GenBank, CCDS, Rfam, tRNAs & Comparative Genomics)
PDE2A
ARAP1
STARD10
ATG16L2
FCHSD2
MIR4692
BC150585
P3
P2
P1
ChromHMM identified states in islets and ENCODE lines
Islets
GM12878
H1 ES
HepG2
HMEC
HSMM
HUVEC
K562
NHEK
NHLF
RNA-seq for Islets
Islets
C
ARAP1
mRNA expression (vs. β-actin)
0.04
0.03
0.02
0.01
0.00
ns
SHAM
dR2
D
ATG16L2
mRNA expression (vs. β-actin)
0.035
0.028
0.021
0.014
0.007
0.000
ns
SHAM
dR2

Suppl. Fig.6

Figure S6. Expression of local genes at *ARAP1*/*STARD10* locus, related to Figure 5.
(A) Genome browser view of the *ARAP1/STARD10* locus displaying the three putative promotors for *ARAP1* (P1-3). Human islet RNA-seq track shows that exons belonging to *ARAP1* isoforms expressed from P1 and P2 are very lowly expressed in comparison to the isoform driven by the P3 promoter. We have modified the scale range to highlight this better. Please note that only P1 belongs to the islet enhancer hub (hub elements track). High PP SNPs track shows all T2D credible set SNPs with PP > 0.05 (Carrat et al., 2017).
(B) Genome browser view of the *ARPA1/STARD10* locus with RNA-seq data. Red box: correlation between promoter region and RNA-seq peak (Parker et al., 2013).
(C) Representative data of *ARAP1* gene expression in dR2 cells. *, $P < 0.05$; **, $P < 0.01$; ***, $P < 0.005$. $n = 3$.
(D) Representative data of *ATG16L2* gene expression in dR2 cells. *, $P < 0.05$; **, $P < 0.01$; ***, $P < 0.005$. $n = 3$.

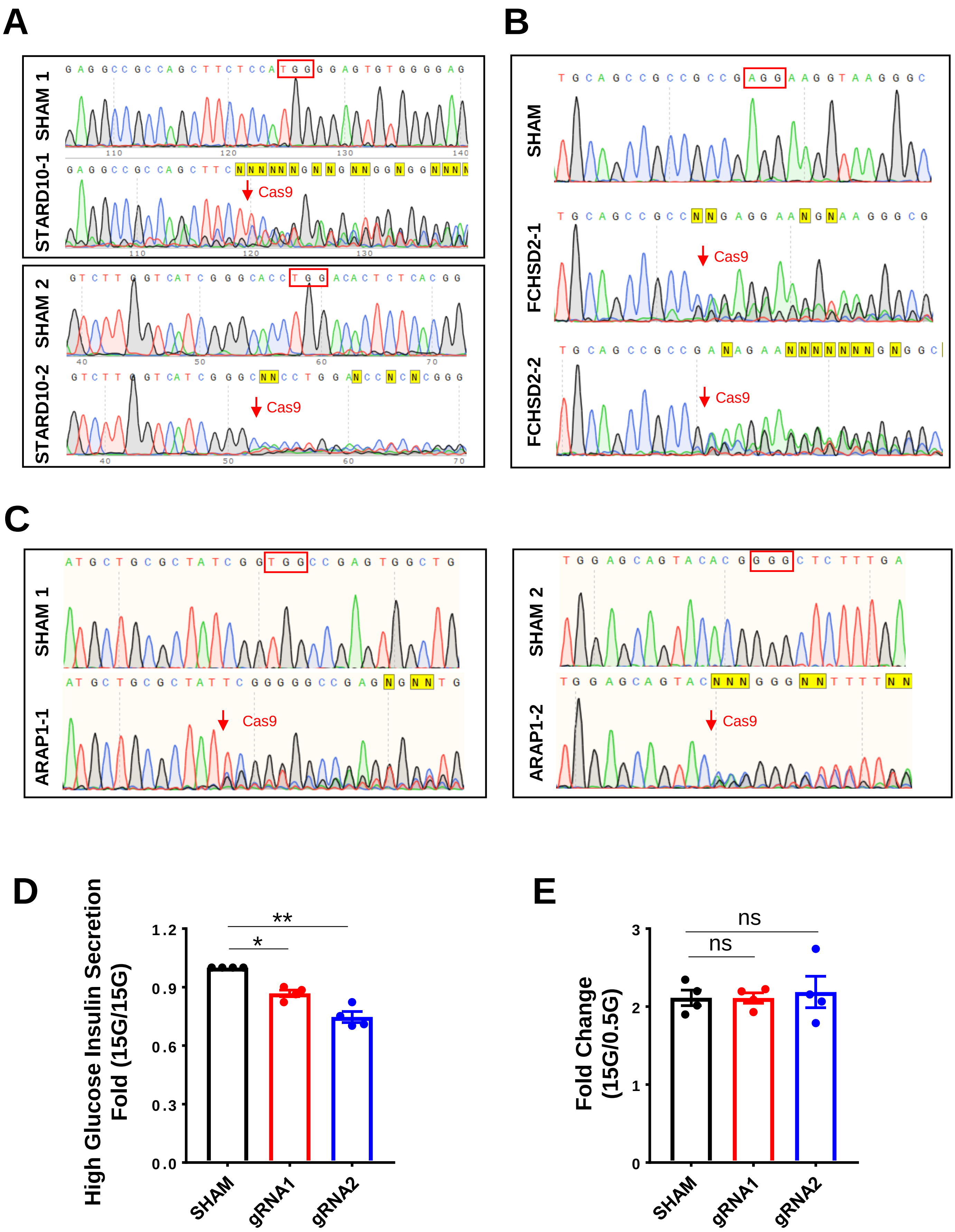
A
SHAM 1
STARD10-1
Cas9
SHAM 2
STARD10-2
Cas9
B
SHAM
FCHSD2-1
Cas9
FCHSD2-2
Cas9
C
SHAM 1
ARAP1-1
Cas9
SHAM 2
ARAP1-2
Cas9
D
High Glucose Insulin Secretion Fold (15G/15G)
1.2
0.9
0.6
0.3
0.0
**
*
SHAM
gRNA1
gRNA2
E
Fold Change (15G/0.5G)
3
2
1
0
ns
ns
SHAM
gRNA1
gRNA2

Suppl. Fig.7

Figure S7. Knockout effects of *STARD10*, *FCHSD2* or *ARAP1* in EndoC-βH1 cells, related to Figure 6.

(A) Saner sequencing of PCR products amplified from wild-type and *STARD10*-KO cells. Red box: PAM sequence; red arrow: Cas9 cutting site.

(B) Sanger sequencings of PCR products amplified from wild-type and *FCHSD2*-KO cells. Red box: PAM sequence; red arrow: Cas9 cutting site.

(C) Sanger sequencings of PCR products amplified from wild-type and *ARAP1*-KO cells. Red box: PAM sequence; red arrow: Cas9 cutting site.

(D-E) GSIS assay of *FCHSD2*-KO cells. (D) Fold change at high glucose level (15 mM glucose vs. 15 mM glucose) (E) Fold change of secreted insulin at 15 mM glucose vs. 0.5 mM glucose. Data are drawn from 4 independent experiments (n = 4). Data are mean ± SEM. *, P < 0.05; **, P < 0.01; ***, P < 0.005.

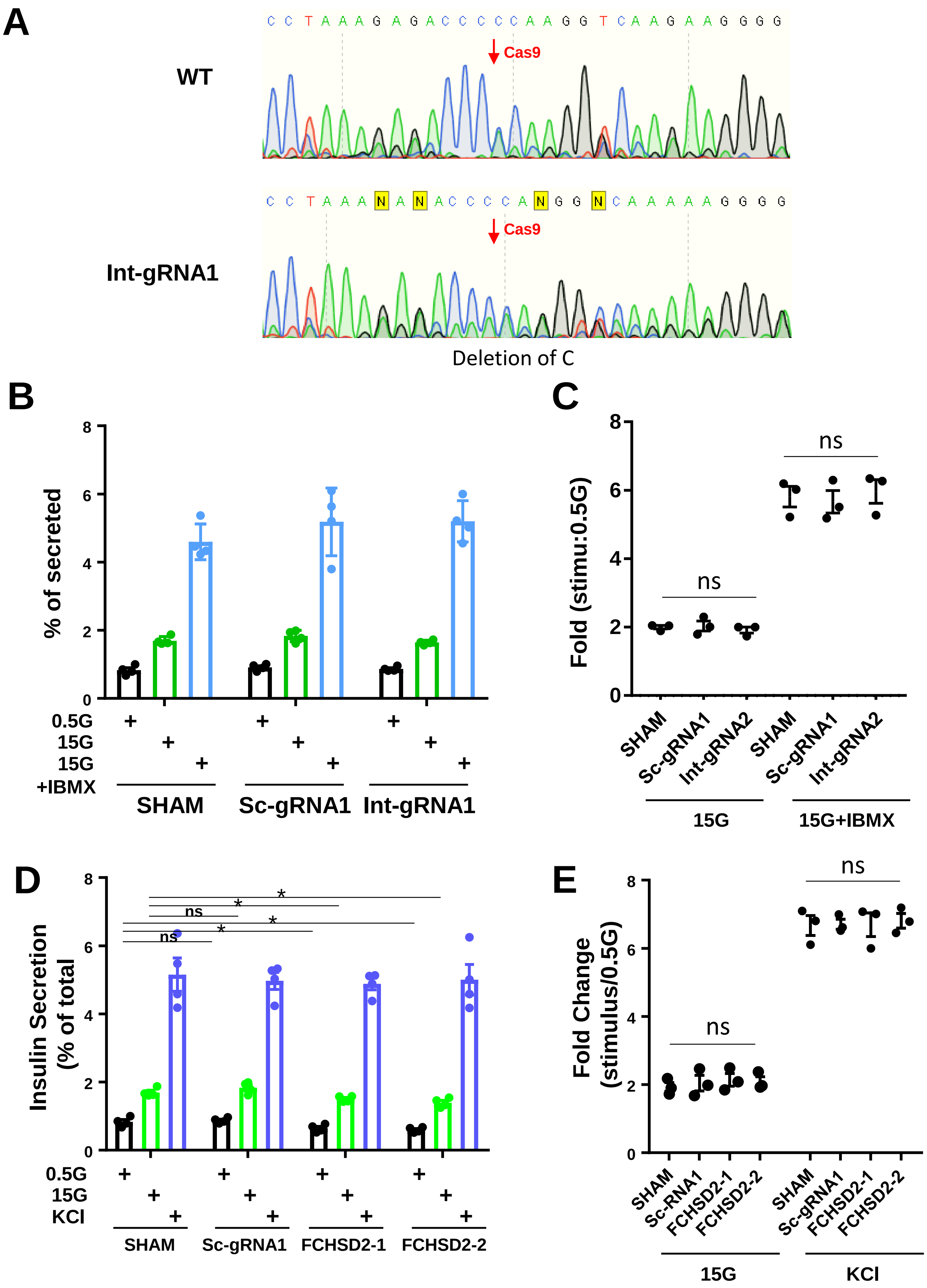
A
WT
C C T A A A G A G A C C C C C A A G G T C A A G A A G G G G
Cas9
Int-gRNA1
C C T A A A N A N A C C C C A N G G N C A A A A A G G G G
Cas9
Deletion of C
B
% of secreted
0.5G
15G
15G
+IBMX
SHAM
Sc-gRNA1
Int-gRNA1
C
Fold (stimu:0.5G)
ns
ns
SHAM
Sc-gRNA1
Int-gRNA2
SHAM
Sc-gRNA1
Int-gRNA2
15G
15G+IBMX
D
Insulin Secretion
(% of total
ns
ns
*
*
*
*
0.5G
15G
KCl
SHAM
Sc-gRNA1
FCHSD2-1
FCHSD2-2
E
Fold Change
(stimulus/0.5G)
ns
ns
SHAM
Sc-RNA1
FCHSD2-1
FCHSD2-2
SHAM
Sc-gRNA1
FCHSD2-1
FCHSD2-2
15G
KCl

Suppl. Fig.8

Figure S8. Effects of DNA mutation in β cell function, related to Figure 6.

(A) Sanger sequencing of PCR products amplified from wild-type and intergenic region gRNA1-cutting (int-gRNA1) cells. Red box: PAM sequence; red arrow: Cas9 cutting site.

(B-C) GSIS assay of CRISPR-Cas9 control cells. (B) Representative data of insulin secretion. (C) Fold change of secreted insulin. Data are normalized to insulin secretion at basal level (0.5 mM glucose) and drawn from 3 independent experiments (n = 3). Data are mean ± SEM. *, $P < 0.05$; **, $P < 0.01$; ***, $P < 0.005$.

(D-E) GSIS assay of *FCHSD2*-KO cells stimulated by KCl. (D) Representative data of insulin secretion. (E) Fold change of secreted insulin. Data are normalized to insulin secretion at basal level (0.5 mM glucose) and drawn from 3 independent experiments (n = 3). Data are mean ± SEM. *, $P < 0.05$; **, $P < 0.01$; ***, $P < 0.005$.

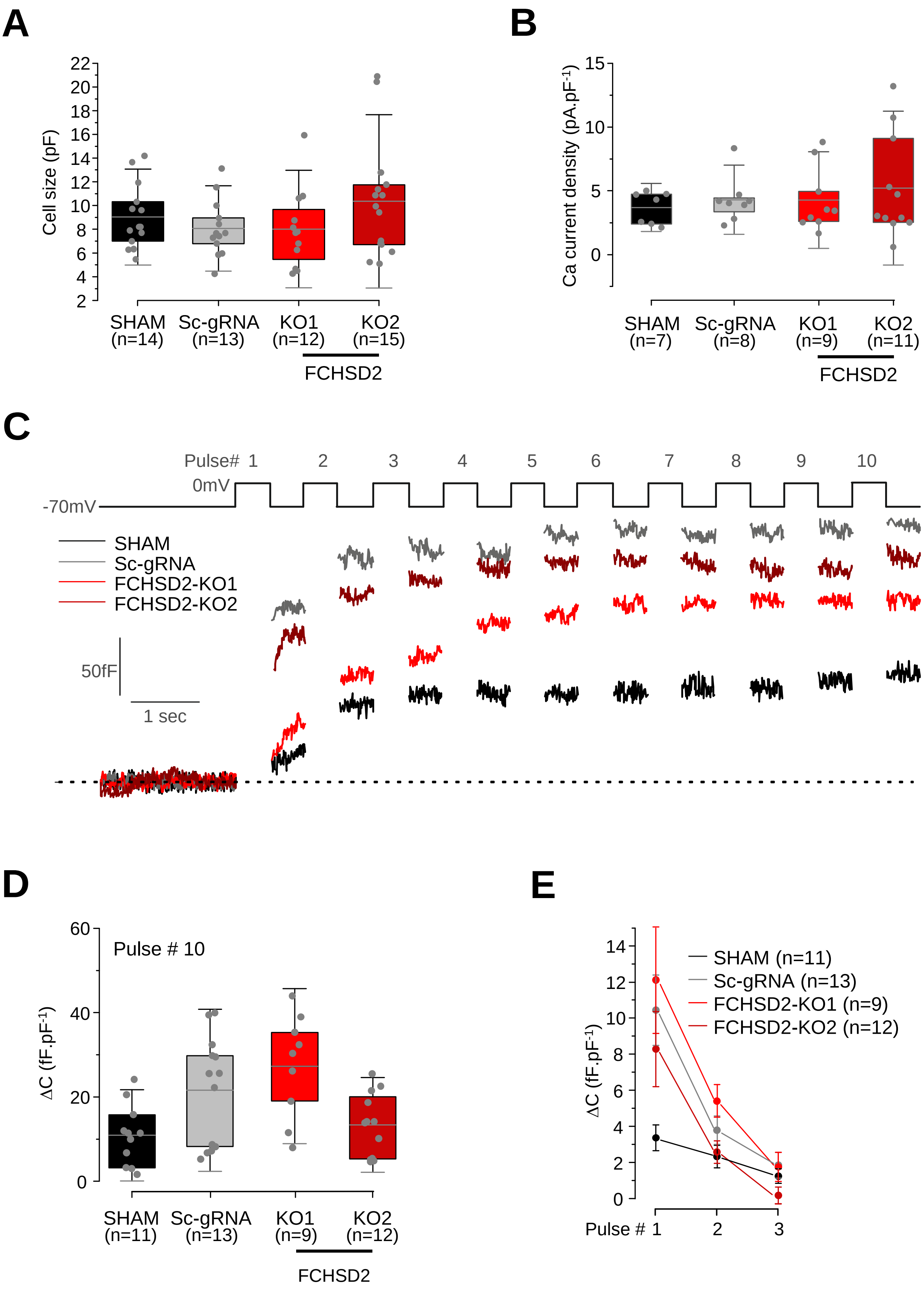
A
Cell size (pF)
22
20
18
16
14
12
10
8
6
4
2
SHAM (n=14)
Sc-gRNA (n=13)
KO1 (n=12)
KO2 (n=15)
FCHSD2
B
Ca current density (pA.pF-1)
15
10
5
0
SHAM (n=7)
Sc-gRNA (n=8)
KO1 (n=9)
KO2 (n=11)
FCHSD2
C
Pulse# 1 2 3 4 5 6 7 8 9 10
0mV
-70mV
SHAM
Sc-gRNA
FCHSD2-KO1
FCHSD2-KO2
50fF
1 sec
D
ΔC (fF.pF-1)
60
40
20
0
Pulse # 10
SHAM (n=11)
Sc-gRNA (n=13)
KO1 (n=9)
KO2 (n=12)
FCHSD2
E
ΔC (fF.pF-1)
14
12
10
8
6
4
2
0
SHAM (n=11)
Sc-gRNA (n=13)
FCHSD2-KO1 (n=9)
FCHSD2-KO2 (n=12)
Pulse # 1 2 3

Suppl. Fig.9

Figure S9. Impact of *FCHSD2*-KO on exocytosis properties, related to Figure 6D-6F.
(A) Comparison of the single cell sizes measured in each cell line (pF).
(B) Calcium current density measured in response to a 100ms depolarisation from -70mV to 0mV. The current density was determined by normalising the amplitude of calcium current (pA) by the cell size (pF).
(C) Representative traces in each cell line of exocytosis elicited by 10 stimulations from -70mV to 0mV.
(D) Amplitude of exocytosis normalised by cell size ($fF.pF^{-1}$) measured at the 10th pulse (Pulse#10).
(E) Increment in exocytosis triggered at each of the three firsts pulses. All box plots (A, B, and C) display the interquartile range (IQR) with means, whiskers indicating 1.5x the SD, and all data points. For clarity, line plots (E) display means ± sem. Ns represents the number of cells measured per cell line and are indicated in each panels. Data were compared using Welch's ANOVA, which does not make the assumption of equal variance in each population, followed by Games-Howell post-hoc test and p-values adjusted by Benjamini & Hochberg procedure.

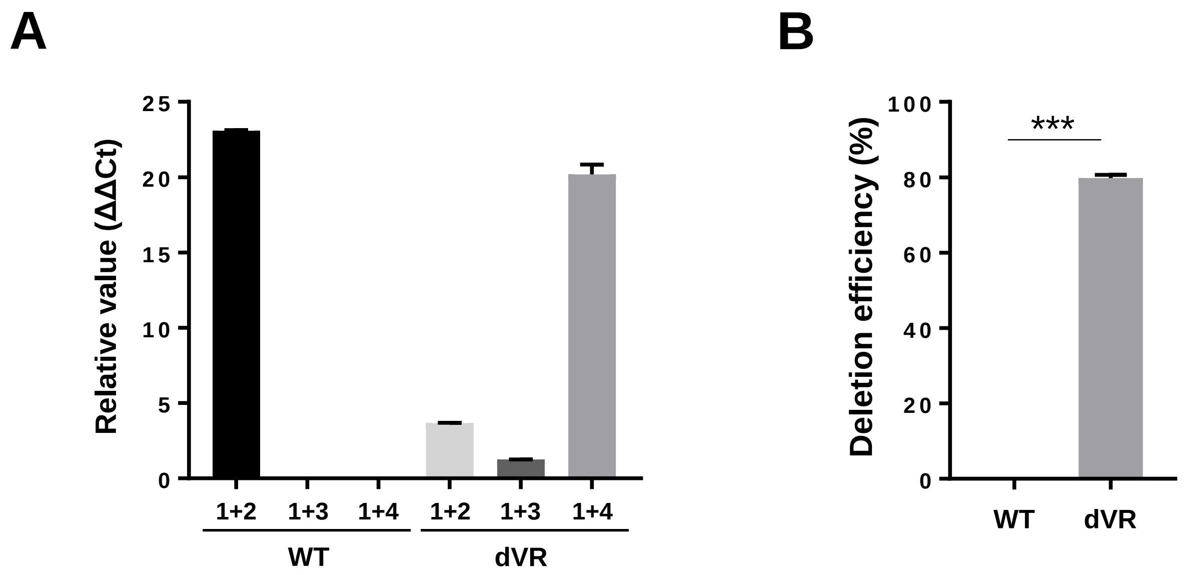

Figure S10. Deletion efficiency of variant region (VR) by CRIPSR-Cas9 genome editing, related to Figure 7.

(A) Representative data of SYBR^{TM} Green qPCR analysis on wildtype and dVR genomic DNAs.

(B) Deletion efficiency. Data are mean ± SEM. *, *P* < 0.05; **, *P* < 0.01; ***, *P* < 0.005. *n* = 1.

Table S1 Transcription factor binding affinity at genetic variants
Related to Fig 1B

variant	gene	Score	
		Risk allele	Protective allele
rs79430446	GATA3	4.6395	6.22367
	GATA5	4.36019	8.01532
	GATA6	6.53008	8.17938
	MAFK	6.15646	-
	NRL	5.70744	-
	MAFF	7.22087	-
	TBP	6.96517	-
	GSC	-	8.9786
	GSC2	-	8.55269
	BARX1	-	6.55349
	BSX	-	5.48796
	EVX1	-	6.53802
	FOXP3	-	5.80405
	OTX2	-	9.73373
	OTX1	-	8.01065
	HOXB2	-	5.44904
	HOXB3	-	4.68114
	MEF2C	-	8.10057
	MEF2A	-	7.64429
	RHOXF1	-	6.92681
	VENTX	-	6.05976
	POU3F4	5.81505	10.7694
	POU5F1B	5.84768	11.6522
	POU5F1	-	8.37144
	POU2F1	-	9.09201
	POU3F1	-	7.69322
	POU3F2	-	7.49311
	POU3F3	-	7.84807
	RFX5	7.526	-
rs140735484	SP1	7.6665	10.0444
	HIC2	-	5.55379
	SP3	-	5.90913
	CDX2	5.8768	-
rs7103836	HLF	7.50884	-
	GATA6	5.78747	-
	SOX10	10.8334	5.84552
	THAP1	6.55426	-
	SOX15	6.50967	-
	RUNX3	5.15967	-
	SOX13	5.39057	-
	FOXP3	5.92425	-
	BARHL2	7.58065	-
rs613937	GSC2	6.72964	-

	OTX1	7.62999	-
	NKX3-2	5.9364	-
	EBF1	4.59291	9.45164
	RFX5	-	5.60305

Table S2 eQTL analysis of *FCHSD2* and *STARD10* gene expression in human islet samples. Related to Fig. 5D

Gene	Variant ID	Position (hg19)	Ref allele	Alt allele	MAF	Risk allele	Nominal P value	rs140130268 R2 (EUR)
FCHSD2	rs11603334	Chr11:72432985	G	A	0.144	G	0.0127082	0.8934
FCHSD2	rs1552224	Chr11:72433098	A	C	0.144	A	0.0127082	0.8934
FCHSD2	rs75896506	Chr11:72429829	G	A	0.144	G	0.0127082	0.8934
STARD10	rs11603334	Chr11:72432985	G	A	0.144	G	2.98×10^{-4}	0.8934
STARD10	rs1552224	Chr11:72433098	A	C	0.144	A	2.98×10^{-4}	0.8934
STARD10	rs75896506	Chr11:72429829	G	A	0.144	G	2.98×10^{-4}	0.8934

Table S3. Primers and probe sequences, related to gRNAs in STAR Methods.

Name	Sequence (R, risk, P, protective allele)
**EMSA assay**	
rs140130268	TATTTGTGGTTTGTTTGTTTGTTTTTCCT (R)
	TATTTGTGGTTTGTTTGTTTTTCCT (P)
rs79430446	GTATAAGATGAGCATGGAAC (R)
	GTATAAGATTAGCATGGAAC (P)
rs140735484	AAATCCCCCAGTCCCAGGCA (R)
	AAATCCCCC-GTCCCAGGCA (P)
rs7103836	TCTGCCACAGAGACATAACA (R)
	TCTGCCACACAGACATAACA (P)
rs613937	CAGCCTCCTAAGCGGCCACA (R)
	CAGCCTCCTGAGCGGCCACA (P)
**genomic DNA PCR amplification**	
VRdel_F1	CCATCTCCCCCGACTCAGCCCAG
VRdel_R1	GGGAGATCCGATTTTGAGTCCCTGC
VRdel_F2	CGACTCAGCCCAGTCTCCTCC
CBS1&2_F	GCAGCCGTGGCCAACACACACACTTCC
CBS1&2_R1	CTCGTGGTGGGGTGCTTGCTGAGG
CBS1&2_R2	GGAGCCCAGAGATGCTGAGAAACTTGC
CBS5_F	CCACTTCCAACCCCAGAGAC
CBS5_R1	CATACTCAGGGGGCCTTGTG
CBS5_R2	CCTTGTGGGGAGGGTCTGGGAG
CBS7&8_F	CACCCCTGGATCTCATTTGATCCTCC
CBS7&8_R1	GCAGTCCTTGAATCCTGATCCTTCCCTGG
CBS7&8_R2	GGTCAGAAGGACGATGCCGAGCGC
R2_F1	GTCAGAAGGCTGAGGCAAGAGGATGG
R2_F2	CAGTGAGCTGAGATGTTGCCACAGC
R2_R	CCACTTTGGTGCCATGTGTGGCCTGG
STARD10_CRISPR_F	CACCCCAGCCCTGCTATAGGTCAGG
STARD10_CRISPR_R	GCCCCCAGCGCACTGATTCCCGTCC
STARD10_CRISPR_R2	GCACTGATTCCCGTCCCCAAACCG
FCHSD2_CRISPR_F1	CTCCCTCGTCTCCTCACACTCG
FCHSD2_CRISPR_F2	TGCCGCCCGCTGGCCTGCTCC
FCHSD2_CRISPR_R	CCCAAGACGAGGGCGGTCACG
ARAP1_CRISPR_F1	CTATGGCTTGAGAGAGCCATGCAGG
ARAP1_CRISPR_F2	GCTGCTGGTGAGGGGACCAATCC
ARAP1_CRISPR_R	CTTCATGGGCACAGGCCGTGG
HBB-F	GACTGGGAGAGAGGACAAGGACC
HBB-R1	GAGTGAGATTTTTTCACAAGTACCTGATGAGG
HBB-R2	GTACCTGATGAGGGTTGAGACAGG
Int-del-F1	CCGGATTTCACCACATTGGCCAGG
Int-del-F2	CTGACGTCAAGTGATGCACCTGC
Int-del-R	CACTCCAATGCCCACCTGTTGTGG
**SYBR Green qPCR assay**	
dVR_1	CCTTCTGGGCTCCCACACAATGC
dVR_2	CTGCCCCAAATGTTCAACACGC

dVR_3	TGTTCAACACGCACTCATTCTTCACC
dVR_4	GTTGCTGAATCCCCCAAGCTTCAG
dR2_1	GCTGGCTCGGCCTTGAGAGG
dR2_2	GGCAAACACTTAGATCCGGCTCC
dR2_3	TTCTCCTAATGTCCTGTCACAGTCCC
dR2_4	ACTTTGGTGCCATGTGTGGCCTGG
qHBB_1	AACACTATGCTAATAACTGCAGAGCCAG
qHBB_2	GATTAGCATTCAGGAAGAGATCAGAGG
qHBB_3	GGCCCTGTCAGTCATCCTGAATCC
qHBB_4	GCTGAAAGGAAGAAGTAGGAGAAACATGC
qInt_1	AGCATGAGTCACTGTGCCCAGC
qInt_2	GCCTGGGTGACAGAGCAAGACTCC
qInt_3	GAAAGAAAGAAATGTGGTTCTAAGGAAAGG
qInt_4	CTAAAACTAAACTTACCACCTTCCTCCCC

4C assay at VR region

4C_F1	GGTATAGCCTGCTACTCAAAAGGTCCCTGG
4C_R1	TACCTCCTGCACTGAGATTCTCCATGAAGC
4C_F2_XhoI	AATTTCTCGAGCTTCTTGTGCCAGGAAGTAAGC
4C_R2-NotI	ATGTGCGGCCGCGGACATCTCCCCATTTCCAAGG

Taqman™ primer/probe for 3C assay

Probe at VR region	AGATGTCCTAAAGTGCTCATTGGGGGCATA
Constant primer	CAGTGAAAGGAGGAGCCCACC
NcoI Fragment	
-13	ACTTCTGTGAGCTCCCTGAGG
-12	ACCTTGTCCGCTCTCAGTCC
-11	CTCTCAGAGCCTGTCTGAACATAGC
6	CCCTTGGGGCTCTGTAGAGG
7	CCAAACCCACACCTGGAACAGG
8	CCAGCTCCACCGCTCCAAGG
9	ACAGGCGTGAGCCATCATGC
Probe at R13 region	CCAGGCCTGGCCCTGTGCTGGCTCCTGAGG
Constant primer	ACTTCTGTGAGCTCCCTGAGG
NcoI Fragment	
12	CCCAACCTTTTTGGCACCAGG
13	CCGTGATGTCATCACCCTCC
14	CCTCCTGCACTGAGATTCTCC
15	GCAGCTTATCTCAGATTGAGCCC
17	CCTGGGTCCCTAGGACTTTGG
18	CTGGCAGAGGTGGTTTGAGC
19	CGGAGCCTCCGCGGAGGACC
20	CCAGAACACCAGGGACTCACG
21	ACTCCCCAGCCAGGTGAAGC
23	CAAGCGTGAGCCACTGCACC

Taqman™ primer for gene expression (Thermo Fisher Scientific)

CXCL12	Cat# 4400291; Assay ID: Hs02117611_cn
STARD10	Cat# 4331182; Assay ID: Hs00246405_ml
ARAP1	Cat# 4331182; Assay ID: Hs00362929_ml
FCHSD2	Cat# 4331182; Assay ID: HS00207952_ml

ATG16L2	Cat# 4331182; Assay ID: Hs01057324_ml
ACTB	Cat# 4331182; assay ID: Hs01060665_gl

SYBR Green qPCR assay at CTCF binding site (CBS)

CBS1_F	AAAGTCACCCAGAATCCCCC
CBS1_R	CAGGGCTTGTCAGTCAGGAC
CBS2_F	ATCAGCCTCCAGGAAGACCA
CBS2_R	GGTGTCCCCCTCTGATACCT
CBS3_F	CTCAGCCACCACATGACCTT
CBS3_R	GGGAGTCCCATCACAGTGTC
CBS4_F	CCAGTCAGGGTCCATGTTGG
CBS4_R	GGTCTTCAAAGCCCTGTGGT
CBS5_F	CCACAAGCTGATGGGGTTGA
CBS5_R	ACCTGGAGGGAAGCTCAGAT
CBS6_F	CACCAAATCTCCCTCACCCC
CBS6_R	AATCTCCTTCACAGACGCCC
Neg_F	AAAGGCCACAAACTCCCCAT
Neg_R	ATTTGGCAGAGCTGAGCGTT
CBS7_F	CAGAACGATGACTGGACCGT
CBS7_R	GGCCGCTGTAAACACCAAAG
CBS8_F	CAGAACGATGACTGGACCGT
CBS8_R	GGCCGCTGTAAACACCAAAG

Cloning of active enhancer region

R2_F	ACTGAGCTAGCGCAGTGAGCTGAGATGTTGC
R2_R	ACTGACTCGAGATCTGGCAACTCCACTTTGG
R4_F	ACTGAGCTAGCGAGTGGTCTGTTGCAGTCAGC
R4_R	ACTGACTCGAGGGTTTCACCACATTGGCCAGG
R5_F	ACTGAGCTAGCGGACAGAGTAACCTTAAGACACAGG
R5_R	ACTGACTCGAGCCAGAGAGGTGATGAGTCTTGAGG
R6_F	ACTGAGCTAGCTGGAAGGACACAGAGCACAG
R6_R	ACTGACTCGAGTCTCTGCCTCCCTTTCTCAG
R7_F	ACTGAGCTAGCGCAGGTGAAGAAACTGAGGC
R7_R	ACTGACTCGAGAACGTACTCAAGCCCAGCAT
R8_F	ACTGAGCTAGCCCTGCTCCTTACAGCCTCAC
R8_R	ACTGACTCGAGCCCTGTTCTTTGCTGTCCTC
